# Supplementary material for: Impact of Phenylketonuria on the Serum Metabolome and Plasma Lipidome: A Study in Early-Treated Patients
Source: Metabolites. 2024 Aug 30;14(9):479. doi: 10.3390/metabo14090479 (PMC11434371; doi:10.3390/metabo14090479)
Supplement: Supplementary file 1 [file metabolites-14-00479-s001.zip › metabolites-3138126-supplementary.pdf]

Impact of Phenylketonuria on the Serum Metabolome and Plasma Lipidome: A Study in Early-Treated Patients

Jorine C. van der Weerd [1], Annemiek M. J. van Wegberg [2], Theo S. Boer [1], Udo F. H. Engelke [3], Karlien L. M. Coene [3,4], Ron A. Wevers [3], Stephan J. L. Bakker [5], Pim de Blaauw [1], Joost Groen [1], Francjan J. van Spronsen [2] and M Rebecca Heiner-Fokkema [1,*]

[1] Department of Laboratory Medicine, Laboratory of Metabolic Disease, University of Groningen, University Medical Center Groningen, 9700 RB Groningen, The Netherlands; j.groen@umcg.nl (J.G.)
[2] Division of Metabolic Diseases, Beatrix Children's Hospital, University of Groningen, University Medical Center Groningen, 9700 RB Groningen, The Netherlands
[3] Department of Human Genetics, Translational Metabolic Laboratory (TML), Radboud University Medical Center, 6525 GA Nijmegen, The Netherlands
[4] Laboratory of Clinical Chemistry and Hematology, Máxima Medical Centre, 5504 DB Veldhoven, The Netherlands
[5] Division of Nephrology, Department of Internal Medicine, University Medical Center Groningen, University of Groningen, 9700 RB Groningen, The Netherlands; s.j.l.bakker@umcg.nl
* Correspondence: m.r.heiner@umcg.nl

Table S1. Overview lipid standards.

No.	Chemical name	Abbreviation	Source
1	1-(10Z-heptadecenoyl)-2-hydroxy-sn-glycero-3-phosphocholine	LPC(17:1/0:0)[I]	Avanti Polar Lipids Inc.
2	1,2-diheptadecanoyl-sn-glycero-3-phosphocholine	PC(17:0/17:0)[I]	Avanti Polar Lipids Inc.
3	1-(10Z-heptadecenoyl)-sn-glycero-3-phosphoethanolamine	LPE(17:1/0:0)[I]	Avanti Polar Lipids Inc.
4	1,2-diheptadecanoyl-sn-glycero-3-phosphoethanolamine	PE(17:0/17:0)[II]	Avanti Polar Lipids Inc.
5	1-(10Z-heptadecenoyl)-sn-glycero-3-phospho-(1′-rac-glycerol)	LPG(17:0/0:0)[I]	Avanti Polar Lipids Inc.
6	1,2-diheptadecanoyl-sn-glycero-3-phospho-(1′-rac-glycerol)	PG(17:0/17:0)[II]	Avanti Polar Lipids Inc.
7	1-(10Z-heptadecenoyl)-2-hydroxy-sn-glycero-3-phospho-(1′-myo-inositol)	LPI(17:1/0:0)[I]	Avanti Polar Lipids Inc.
8	1-heptadecanoyl-2-(5Z,8Z,11Z,14Z-eicosatetraenoyl)-sn-glycero-3-phospho-(1′-myo-inositol)	PI (17:0/20:4)[I]	Avanti Polar Lipids Inc.
9	1-(10Z-heptadecenoyl)-2-hydroxy-sn-glycero-3-[phospho-L-serine]	LPS (17:1/0:0)[I]	Avanti Polar Lipids Inc.
10	1,2-diheptadecanoyl-sn-glycero-3-phospho-L-serine	PS (17:0/17:0)[II]	Avanti Polar Lipids Inc.
11	1′,3′-bis[1,2-dimyristoyl-sn-glycero-3-phospho]-glycerol	CL(14:0/14:0) (14:0/14:0)[II]	Avanti Polar Lipids Inc.
12	1′,3′-bis[1,2-dipalmitoyl-sn-glycero-3-phospho]-glycerol	CL(16:0/16:0) (16:0/16:0)[II]	Avanti Polar Lipids Inc.
13	1,2-dipalmitoyl-sn-glycerol	DG (16:0/16:0/0:0)[I]	Avanti Polar Lipids Inc.
14	1,3(d5)-diheptadecanoyl-2-(10Z-heptadecenoyl)-glycerol	TG (17:0/17:1/17:0 d5)[II]	Avanti Polar Lipids Inc.

[I] Lipids dissolved in chloroform
[II] Lipids dissolved in $CHCl_3$/MeOH/ Milli-Q water (60:30:4.5, v/v/v)

Table S2. UPLC and MS settings in ESI(+) and ESI(-).

Parameter	ESI(+)	ESI(-)
Lock Spray Configuration		
Reference Scan Frequency (sec)	30	30
Reference Cone Voltage (V)	30	60
Reference Trap Collision Energy	4	4
Reference DRE Setting	99.9	99.9
Instrument Configuration		
Lteff	3600	1800
Veff	7217.8	7210.8
Resolution	40000	20000
Min Points in Peak	2	2
Acquisition Device	WatersADC	WatersADC
Acquisition Algorithm	ADC Mode	ADC Mode
ADC Trigger Threshold (V)	1	-1
ADC Input Offset (V)	-1.4	-1.4
Average Single Ion Intensity	21	19
ADC Amplitude Threshold	4	5
ADC Centroid Threshold	-1	-1
ADC Ion Area Threshold	3	1
ADC Ion Area Offset	10	10
ADC Pushes Per IMS Increment	1	1
EDC Delay Coefficient	1.41	1.41
EDC Delay Offset	0.4	0.4
Experimental Instrument Parameters		
Polarity	ES+	ES-
Capillary (kV)	0.5	0.7
Source Temperature (°C)	120	120
Sampling Cone	20	20
Source Offset	20	20
Source Gas Flow (mL/min)	0	0
Desolvation Temperature (°C)	600	600
Cone Gas Flow (L/Hr)	100	100
Desolvation Gas Flow (L/Hr)	1000	1000
Nebuliser Gas Flow (Bar)	6.5	6.5
LM Resolution	15	4.7
HM Resolution	15	15
Aperture 1	0	0
Pre-filter	2	2
Ion Energy	0.2	0.8
Manual Trap Collision Energy	TRUE	TRUE
Trap Collision Energy	2	2
Manual Transfer Collision Energy	TRUE	TRUE
Transfer Collision Energy	1	1

Parameter	ESI(+)	ESI(-)
Manual Gas Control	FALSE	FALSE
Trap Gas Flow (mL/min)	2	2
HeliumCellGasFlow	180	180
IMS Gas Flow (mL/min)	90	90
Detector	3150	3462
DetectorCache	2300	2200
Sample Infusion Flow Rate (µL/min)	5	5
Sample Flow State	LC	LC
Sample Fill Volume (µL)	250	250
Sample Reservoir	Wash	Wash
LockSpray Infusion Flow Rate (µL/min)	20	20
LockSpray Flow State	Infusion	Infusion
LockSpray Reservoir	B	B
LockSpray Capillary (kV)	3	2.5
Use Manual LockSpray Collision Energy	FALSE	FALSE
Collision Energy	4	4
Acceleration1	10	70
Acceleration2	0	200
Aperture2	5	70
Transport1	30	70
Transport2	20	70
Steering	0	0
Tube Lens	25	75
Pusher	1900	1900
Pusher Offset	-0.6	-0.1
Puller	1307	1310
Pusher Cycle Time (µs)	Automatic	Automatic
Pusher Width (µs)	Automatic	Automatic
Collector	60	60
Collector Pulse	10	10
Stopper	10	10
Stopper Pulse	20	20
Entrance	17	62
Static Offset	180	180
Puller Offset	0	0
Reflectron Grid (kV)	1.334	1.48
Flight Tube (kV)	10	10
Reflectron (kV)	4.14	3.78
Use Manual Trap DC	TRUE	FALSE
Trap DC Entrance	1	1
Trap DC Bias	2	2
Trap DC	-2	-2
Trap DC Exit	0	0
Use Manual IMS DC	FALSE	FALSE
IMS DC Entrance	-20	-20

Parameter	ESI(+)	ESI(-)
Helium Cell DC	1	1
Helium Exit	-20	-20
IMSBias		2
IMS DC Exit	20	20
USe Manual Transfer DC	FALSE	FALSE
Transfer DC Entrance	5	5
Transfer DC Exit	3	15
Trap Manual Control	OFF	OFF
Trap Wave Velocity (m/s)	300	300
Trap Wave Height (V)	0.5	0.5
IMS Manual Control	OFF	OFF
IMS Wave Velocity (m/s)	300	300
IMS Wave Height (V)	0	0
Transfer Manual Control	OFF	OFF
Transfer Wave Velocity (m/s)	247	247
Transfer Wave Height (V)	0.2	0.2
Step Wave 1 In Manual Control	OFF	OFF
Enable Reverse Operation	OFF	OFF
Step Wave 1 In Velocity (m/s)	300	300
Step Wave 1 In Height	15	15
Step Wave 1 Out Manual Control	OFF	OFF
Step Wave 1 Out Velocity (m/s)	300	300
Step Wave 1 Out Height	15	15
Step Wave 2 Manual Control	OFF	OFF
Step Wave 2 Velocity (m/s)	300	300
Step Wave 2 Height	1	1
Use Manual Step Wave DC	ON	OFF
Step Wave TransferOffset	5	25
Step Wave DiffAperture1	3	3
Step Wave DiffAperture2	0	0
Use Automatic RF Settings	TRUE	TRUE
StepWave1RFOffset	100	100
StepWave2RFOffset	150	150
Target Enhancement Enabled	FALSE	FALSE
Target Enhancement Mode	EDC	EDC
Target Enhancement Mass	556	556
Target Enhancement Trap Height (V)	4	4
Target Enhancement Extract Height (V)	15	15
Mobility Trapping Manual Release Enabled	FALSE	FALSE
Mobility Trapping Release Time (µs)	500	500
Mobility Trap Height (V)	15	15
Mobility Extract Height (V)	0	0
Trag Gate LUT table enabled	FALSE	FALSE
TriWave Trap Gate LookUp Table		
Using Drift Time Trimming	FALSE	FALSE

Parameter	ESI(+)	ESI(-)
Drift Time Bins	0	0
Using Mobility Delay after Trap Release	TRUE	TRUE
IMS Wave Delay (μs)	1000	1000
Variable Wave Height Enabled	FALSE	FALSE
Wave Height Ramp Type	Linear	Linear
Wave Height Start (V)	10	10
Wave Height End (V)	40	40
Wave Height Using Full IMS	TRUE	TRUE
Wave Height Ramp (%)	100	100
Wave Height Look Up Table		
Variable Wave Velocity Enabled	FALSE	FALSE
Wave Velocity Ramp Type	Linear	Linear
Wave Velocity Start (m/s)	1000	1000
Wave Velocity End (m/s)	300	300
Wave Velocity Using Full IMS	TRUE	TRUE
Wave Velocity Ramp (%)	100	100
Wave Velocity Look Up Table	-	
Backing	2.53	2.52
Source	0.00648	0.00648
Sample Plate	0.000001	0.000001
Trap	0.00872	0.00878
Helium Cell	0.000767	0.000772
IMS	0.000618	0.000603
Transfer	0.00862	0.00867
TOF	0.000000491	0.000000478
IMSRFOffset	300	300
IMSMobilityRFOffset	250	250
TrapRFOffset	300	300
Use Automatic RF Settings	TRUE	TRUE
AutoStepWave1RFOffset	300	300
AutoStepWave2RFOffset	350	350
TransferRFOffset	350	350
MS Profile Type	Auto P	Auto P
MSProfileMass1	180	180
MSProfileDwellTime1	20	20
MSProfileRampTime1	20	20
MSProfileMass2	500	500
MSProfileDwellTime2	20	20
MSProfileRampTime2	40	40
MSProfileMass3	1200	1200
PusherInterval	137	69
PusherOffset	0.25	0.25
LockMassValidSigma	5	5
Acquisition mass range		

Parameter	ESI(+)	ESI(-)
Start mass	50	50
End mass	2000	2000
Function Parameters - Function 1 - TOF PARENT FUNCTION		
Acquisition		
Survey Start Time	1	1
Survey End Time	25	25
Survey Ion Mode	ES Mode	ES Mode
Survey Polarity	Positive	Negative
Parent MS Survey		
Survey Start Mass	50	
Survey End Mass	2000	50
Parent Survey Low CE (V)	10	2000
TIC Threshold	5	10
Survey Scan Time	0.5	5
Survey Interscan Time	0	0.5
Survey Data Format	Continuum	0
Analyser	High Resolution Mode	Continuum
ADC Sample Frequency (GHz)	3	Resolution Mode
TargetEnhancementMass2	137	3
TargetEnhancementMass3	1.75	69
Survey Use Tune Page CV	YES	1.75
		YES
Product Ions		
Use High CE Product Ions Mass List File	NO	
High CE Product Ions Mass List Filename		NO
Product Ions Match Logic	NO	
Product Ions Switch Threshold (Intensity/s)	10	NO
Product Ions Switch Detection Window +/- (mDa)	100	10
Product Ions Retention Time Window +/- (sec)	10	100
		10
Neutral loss		
Use Neutral Loss Mass List File	NO	NO
Neutral Loss Mass List Filename		
Neutral Loss Match Logic	OR	OR
Neutral Loss Switch Threshold (Intensity/s)	10	10
Neutral Loss Switch Detection Window +/- (mDa)	100	100
MS/MS		
MSMS Start Mass	50	50
MSMS End Mass	2000	2000

Parameter	ESI(+)	ESI(-)
Number of components	0	0
Use MSMS to MS Switch After Time	NO	NO
MSMS Switch After Time (sec)	10	10
Absence of Neutral Loss	NO	NO
Absence of Product Ion	NO	NO
MSMS Scan Time (sec)	1	1
MSMS Interscan Time (sec)	0	0
MSMS Data Format	Continuum	Continuum
Use Tune Page Cone Voltage	YES	YES
Use MS/MS ipr File	NO	NO
Instrument Parameter Filename		
Peak Detection		
Peak Detection Window	1	1
Use Intensity based Peak Detection	YES	YES
Charge State Tolerance Window	0.2	0.2
Charge State Extraction Window	4	4
Deisotope Tolerance Window	0.2	0.2
Deisotope Extraction Window	4	4
Discard survey data	NO	NO
Collision energy		
Trap MS Collision Energy (eV)	2	2
Transfer MS Collision Energy (eV)	1	1
Include		
Precursor Selection	Everything	Everything
Exclude		
Use Exclude Masses List	NO	NO
Exclude Mass Range	-	-
Use Exclude File Masses	NO	NO
Exclude Mass Filename	-	-
Exclude Window +/- (mDa)	100	100
Exclude Retention Time Window	10	10
Reference Centroid Average	-	-
Reference Frequency	0	0
Reference Cone Voltage	0	0
Calibration	Dynamic 2	Dynamic 2
Function Parameters - Function 2 - TOF PARENT FUNCTION		
Acquisition		
Survey Start Time	1	1
Survey End Time	25	25

Parameter	ESI(+)	ESI(-)
Survey Ion Mode	ES Mode	ES Mode
Survey Polarity	Positive	Negative
Parent MS Survey		
Survey Start Mass	50	50
Survey End Mass	2000	2000
Ramp High Energy from	30.0 to 60.0	30.0 to 60.0
TIC Threshold	5	5
Survey Scan Time	0.5	0.5
Survey Interscan Time	0	0
Survey Data Format	Continuum	Continuum
Analyser	High Resolution Mode	Resolution Mode
ADC Sample Frequency (GHz)	3	3
TargetEnhancementMass2	137	69
TargetEnhancementMass3	1.75	1.75
Survey Use Tune Page CV	YES	YES
Product Ions		
Use High CE Product Ions Mass List File	NO	NO
High CE Product Ions Mass List Filename	-	-
Product Ions Match Logic	NO	NO
Product Ions Switch Threshold (Intensity/s)	10	10
Product Ions Switch Detection Window +/- (mDa)	100	100
Product Ions Retention Time Window +/- (sec)	10	10
Neutral loss		
Use Neutral Loss Mass List File	NO	NO
Neutral Loss Mass List Filename	-	-
Neutral Loss Match Logic	OR	OR
Neutral Loss Switch Threshold (Intensity/s)	10	10
Neutral Loss Switch Detection Window +/- (mDa)	100	100
MS/MS		
MSMS Start Mass	50	50
MSMS End Mass	2000	2000
Number of components	0	0
Use MSMS to MS Switch After Time	NO	NO
MSMS Switch After Time (sec)	10	10
Absence of Neutral Loss	NO	NO
Absence of Product Ion	NO	NO
MSMS Scan Time (sec)	1	1
MSMS Interscan Time (sec)	0	0
MSMS Data Format	Continuum	Continuum

Parameter	ESI(+)	ESI(-)
Use Tune Page Cone Voltage	YES	YES
Use MS/MS ipr File	NO	NO
Instrument Parameter Filename		
Peak Detection		
Peak Detection Window	1	1
Use Intensity based Peak Detection	YES	YES
Charge State Tolerance Window	0.2	0.2
Charge State Extraction Window	4	4
Deisotope Tolerance Window	0.2	0.2
Deisotope Extraction Window	4	4
Discard survey data	NO	NO
Collision energy		
Trap MS Collision Energy Low (eV)	30	30
Trap MS Collision Energy High (eV)	60	60
Using Auto Transfer MS Collision Energy (eV)	2	2
Include		
Precursor Selection	Everything	Everything
Exclude		
Use Exclude Masses List	NO	NO
Exclude Mass Range	-	-
Use Exclude File Masses	NO	NO
Exclude Mass Filename	-	-
Exclude Window +/- (mDa)	100	100
Exclude Retention Time Window	10	10
Reference Centroid Average	-	-
Reference Frequency	0	0
Reference Cone Voltage	0	0
Calibration	Dynamic 2	Dynamic 2
Function Parameters - Function 3 - TOF PARENT FUNCTION		
Acquisition		
Survey Start Time	1	1
Survey End Time	25	25
Survey Ion Mode	ES Mode	ES Mode
Survey Polarity	Positive	Negative
Parent MS Survey		
Survey Start Mass	50	50
Survey End Mass	2000	2000
Parent Survey High CE (V)	30	30

Parameter	ESI(+)	ESI(-)
TIC Threshold	5	5
Survey Scan Time	0.5	0.5
Survey Interscan Time	0.1	0.1
Survey Data Format	Continuum	Continuum
Analyser	High Resolution Mode	Resolution Mode
ADC Sample Frequency (GHz)	3	3
TargetEnhancementMass2	137	69
TargetEnhancementMass3	1.75	1.75
Survey Use Tune Page CV	YES	YES
Product Ions		
Use High CE Product Ions Mass List File	NO	NO
High CE Product Ions Mass List Filename	-	-
Product Ions Match Logic	NO	NO
Product Ions Switch Threshold (Intensity/s)	10	10
Product Ions Switch Detection Window +/- (mDa)	100	100
Product Ions Retention Time Window +/- (sec)	10	10
Neutral loss		
Use Neutral Loss Mass List File	NO	NO
Neutral Loss Mass List Filename	-	-
Neutral Loss Match Logic	OR	OR
Neutral Loss Switch Threshold (Intensity/s)	10	10
Neutral Loss Switch Detection Window +/- (mDa)	100	100
MS/MS		
MSMS Start Mass	100	100
MSMS End Mass	1500	1500
Number of components	1	1
Use MSMS to MS Switch After Time	NO	NO
MSMS Switch After Time (sec)	10	10
Absence of Neutral Loss	NO	NO
Absence of Product Ion	NO	NO
MSMS Scan Time (sec)	1	1
MSMS Interscan Time (sec)	0.1	0.1
MSMS Data Format	Continuum	Continuum
Use Tune Page Cone Voltage	YES	YES
Use MS/MS ipr File	NO	NO
Instrument Parameter Filename		
Peak Detection		
Peak Detection Window	1	1
Use Intensity based Peak Detection	YES	YES

Parameter	ESI(+)	ESI(-)
Charge State Tolerance Window	0.2	0.2
Charge State Extraction Window	4	4
Deisotope Tolerance Window	0.2	0.2
Deisotope Extraction Window	4	4
Discard survey data	NO	NO
Collision energy		
Using Auto Trap MS Collision Energy (eV)	4	4
Using Auto Transfer MS Collision Energy (eV)	2	2
Include		
Precursor Selection	Everything	Everything
Exclude		
Use Exclude Masses List	NO	NO
Exclude Mass Range	-	-
Use Exclude File Masses	NO	NO
Exclude Mass Filename	-	-
Exclude Window +/- (mDa)	100	100
Exclude Retention Time Window	10	10
Reference Centroid Average	-	-
Reference Frequency	0	0
Reference Cone Voltage	0	0
Pumps Waters Acquity SDS		
Run Time	30.00 min	30.00 min
Comment	-	-
Solvent Selection A	A1	A1
Solvent Selection B	B1	B1
Low Pressure Limit	0 psi	0 psi
High Pressure Limit	15000 psi	15000 psi
Solvent Name A	Water	Water
Solvent Name B	Methanol	Methanol
Switch 1	No Change	No Change
Switch 2	No Change	No Change
Switch 3	No Change	No Change
Seal Wash	2.0 min	2.0 min
Chart Out 1	System Pressure	System Pressure
Chart Out 2	%B	%B
System Pressure Data Channel	No	No
Flow Rate Data Channel	No	No
%A Data Channel	No	No
%B Data Channel	No	No
Primary A Pressure Data Channel	No	No
Accumulator A Pressure Data Channel	No	No

Parameter	ESI(+)	ESI(-)
Primary B Pressure Data Channel	No	No
Accumulator B Pressure Data Channel	No	No
Degasser Pressure Data Channel	No	No
Gradient settings		
Time(min)	Flow rate	%A;%B
Initial	0.5	40;60
7.5	0.5	10;90
15	0.5	0;100
25	0.5	0;100
25.1	0.5	40;60
Run Events	Yes	Yes
Gradient Start (Relative to Injection)	0 uL	0 uL
Participate in pre-analysis	No	No
Waters ACQUITY FTN AutoSampler		
Run Time	30.00 min	30.00 min
Comment		
Load Ahead	Disabled	Disabled
Loop Offline	Automatic min	Automatic min
Wash Solvent Name	Magic Mix(ACN/MEOH/IPA/MQ 1:1:1 v/v/v + 0.1%FA	
Pre-Inject Wash Time	0.0 sec	0.0 sec
Post-Inject Wash Time	15.0 sec	15.0 sec
Purge Solvent Name	50% MeOH	50% MeOH
Dilution	Disabled	Disabled
Dilution Volume	0 uL	0 uL
Delay Time	0 min	0 min
Dilution Needle Placement	Automatic mm	Automatic mm
Target Column Temperature	80.0 C	80.0 C
Column Temperature Alarm Band	Disabled	Disabled
Target Sample Temperature	15.0 C	15.0 C
Sample Temperature Alarm Band	Disabled	Disabled
Syringe Draw Rate	Automatic	Automatic
Needle Placement	3.0 mm	3.0 mm
Pre-Aspirate Air Gap	Automatic	Automatic
Post-Aspirate Air Gap	Automatic	Automatic
Column Temperature Data Channel	No	No
Room Temperature Data Channel	No	No
Sample Temperature Data Channel	No	No
Sample Organizer Temperature Data Channel	No	No
Sample Pressure Data Channel	No	No
Preheater Temperature Data Channel	No	No
Seal Force Data Channel	No	No
No Injection Mode Enabled	No	No

Parameter	ESI(+)	ESI(-)
Run Events	No	No
Injection Volume (ul)	2	2
MS functions		
Function 1		
Scans in function	1368	1368
Cycle time (secs)	0.515	0.515
Scan duration (secs)	0.5	0.5
Inter Scan Delay (secs)	0.015	0.015
Start and End Time(mins)	1 to 25.000	1.000 to 25.000
Ionization mode	ES+	ES-
Data type	Enhanc	Enhanced Mass
Function type	TOF MS	TOF MS
Mass range	50 to 2000	50 to 2000
Function 2		
Scans in function:	1368	1368
Cycle time (secs)	0.515	0.515
Scan duration (secs)	0.5	0.5
Inter Scan Delay (secs)	0.015	0.015
Start and End Time(mins)	1 to 25.000	1.000 to 25.000
Ionization mode	ES+	ES-
Data type	Enhanced Mass	Enhanced Mass
Function type	TOF MS	TOF MS
Mass range	50 to 2000	50 to 2000
Function 3		
Scans in function	48	48
Cycle time (secs)	0.6	0.6
Scan duration (secs)	0.5	0.5
Inter Scan Delay (secs)	0.1	0.1
Start and End Time(mins)	1 to 25.000	1.000 to 25.000
Ionization mode	ES+	ES-
Data type	Enhanced Accurate Mass	Enhanced Accurate Mass
Function type	TOF MS	TOF MS
Mass range	50 to 2000	50 to 2000

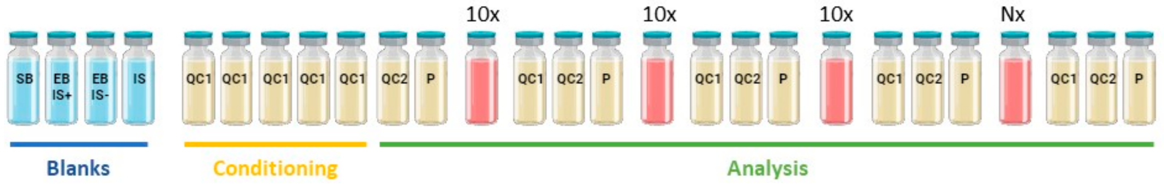

Figure S1. Schematic representation of run order for lipidomics measurements. To check the presence of non-biologically related LC-MS features that represent background effects each batch starts with four blank samples including a system blank, two process blanks (with and without internal standard) and a reconstitution solvent injection. A QC sample was injected five times at the beginning of the batch for system equilibration. Subsequently, two QC samples (low and high TG content) and a pooled sample were injected at regular intervals (every 10 samples, Nx* remaining samples). Data were assessed regarding accurate mass, retention time, and peak area of internal non-physiological and/or isotopically labelled standards. Pre-defined acceptance criteria were: (i) m/z error of 5 ppm ESI(+) for IS lipids, respectively; (ii) RSD% for peak area ≤ 30% all features.

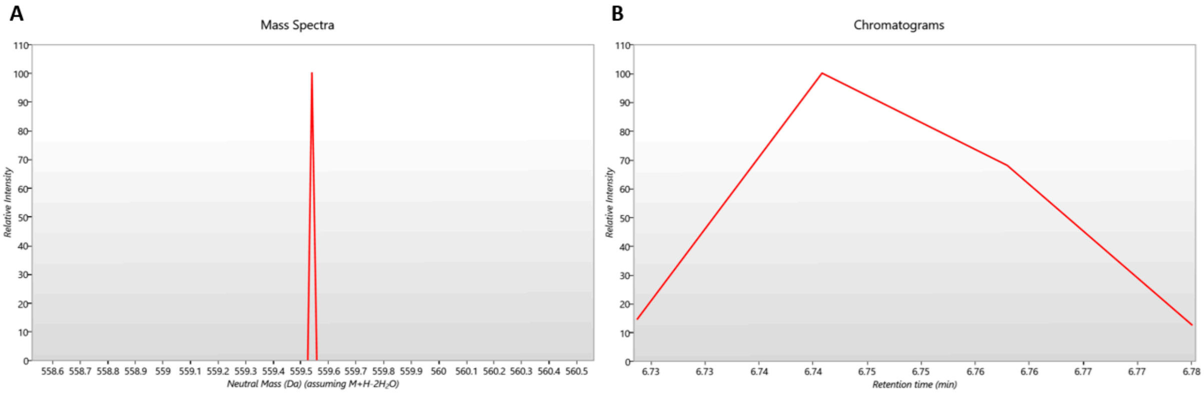

Figure S2. Example absence of isotopic pattern. Peaks were omitted from the analysis if their isotopic pattern manifested as a single peak in the mass spectrum (A), without isotopes this is reflected by the value of 100. The corresponding chromatogram is also shown (B).

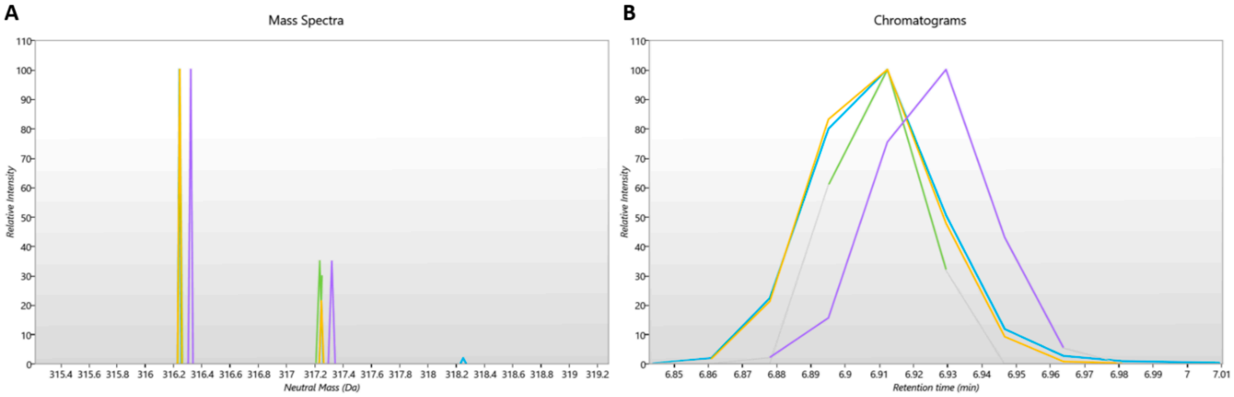

Figure S3. Example of deconvolution failure. The mass spectrum and chromatogram were used to detect deconvolution failures. The spectra of the ions are presented without their charges and adduct masses (see A). If the precursor ions belong to the same compound, their mass spectra should overlay. Unexpected differences in the ion masses, isotope patterns and/or retention times (B) may indicate that they represent different compounds.

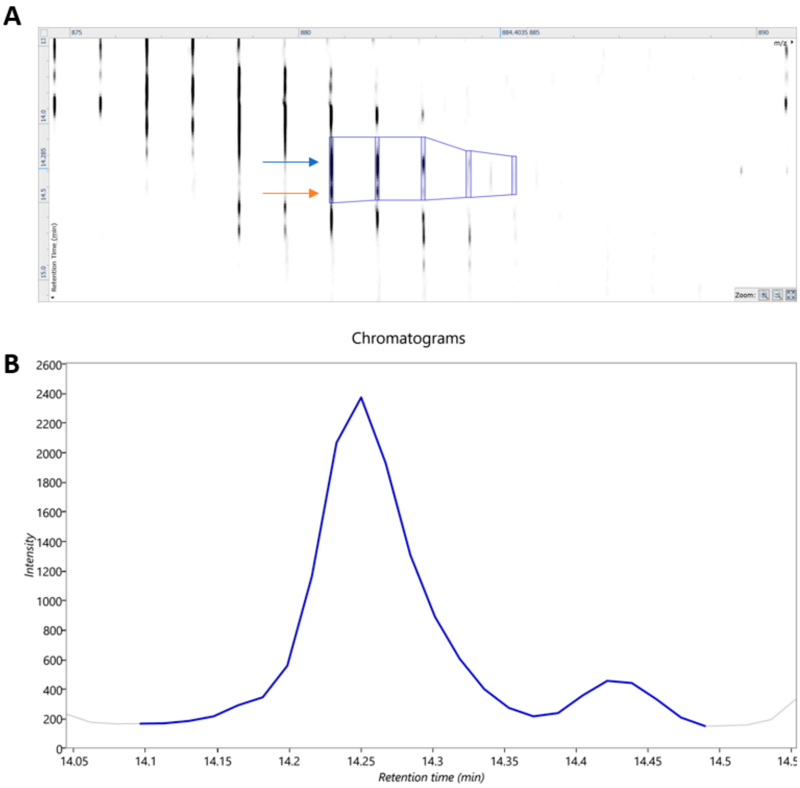

Figure S4. Example of chromatographic resolution failure. Some compounds were found to coelute with other compounds but were recognized as a single feature by Progenesis QI software. With the current software version it was not possible to adjust peak integration settings. Co-eluting peaks were therefore disregarded. These features were identified based on both the spectrum (A) and the chromatogram (B).

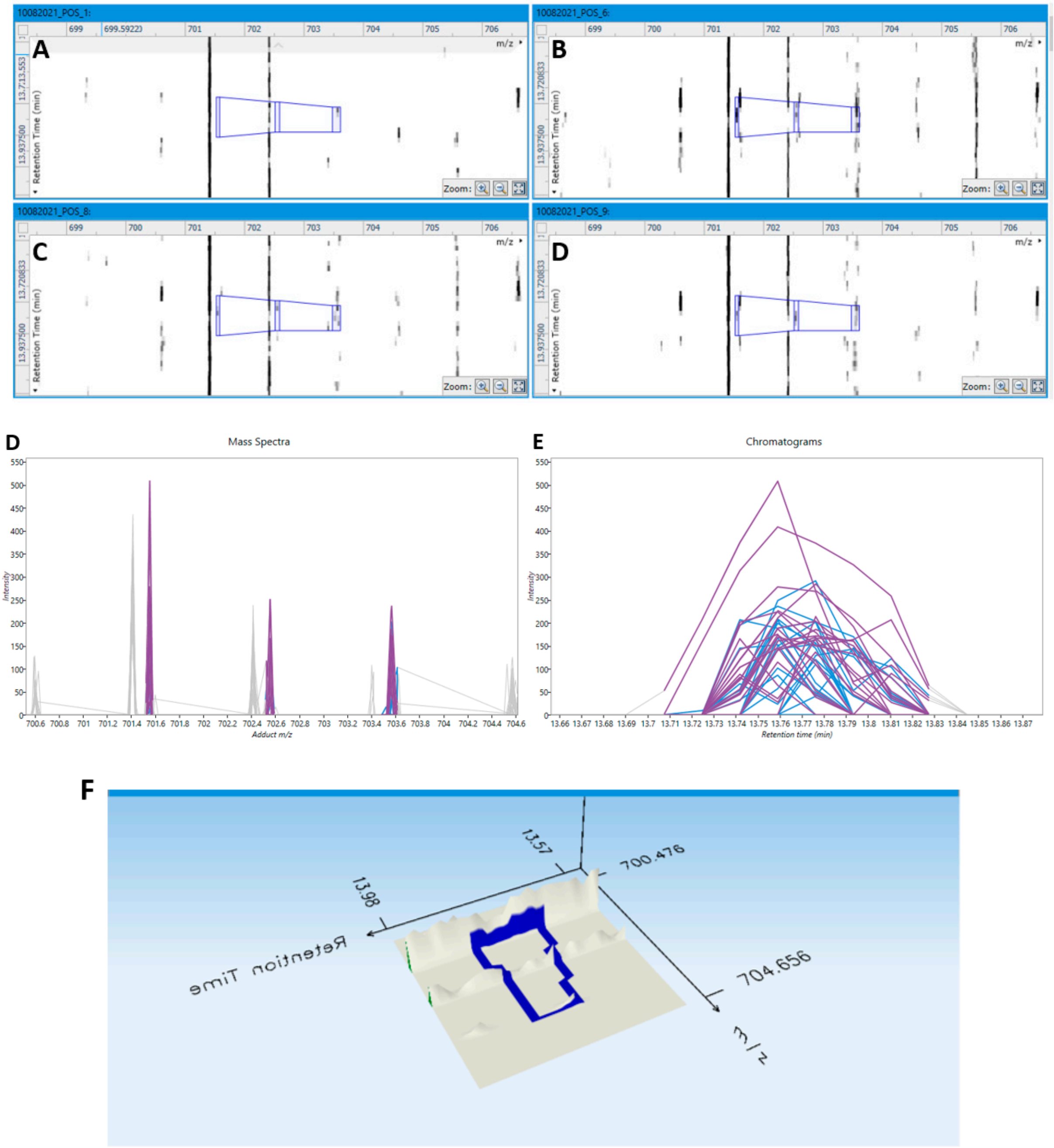

Figure S5. Examples of noise. Features for which a consistent signal was missing. Features for which there was no consistent signal observed among the study samples (A-D).

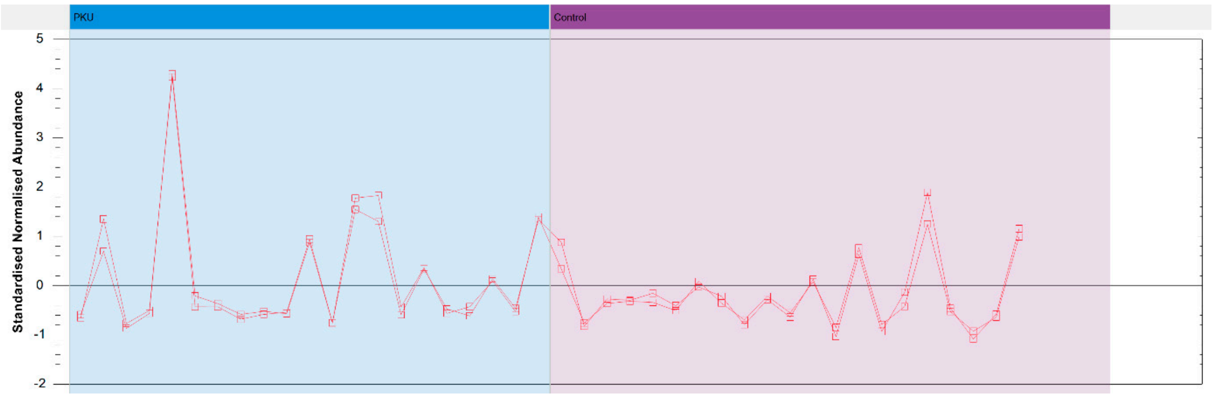

Figure S6. Example of in-source fragmentation. Two features with identical retention time. The standardized normalised abundance profile is highly similar indicating that the both features are part of the same compound.

Table S3. Qualitative fragments of lipids in negative ion mode. List of different lipid classes and their qualitative fragments, in negative ESI, when MS/MS fragmentation is performed.

**Lipid class**	**Precursor ion**	**Fragment**	
PA	$[M-H]^-$	Glycerol phosphate – H_2O $[C_3H_6O_5P]^-$	152.9953 amu
		FA carboxylate anions	*species dependent*
PC	$[M+HCOO]^-$	Neutral loss of methyl group and formate ion $[C_2H_6O_2]$	60.0211 amu
		FA carboxylate anions	*species dependent*
PE	$[M-H]^-$	Glycerol phosphoethanolamine – H_2O $[C_5H_{11}NO_5P]^-$	196.0375 amu
		FA carboxylate anions	*species dependent*
PG	$[M-H]^-$	Glycerol phosphate – H_2O $[C_3H_6O_5P]^-$	152.9953 amu
		Glycerol phosphoglycerol – H_2O $[C_6H_{12}O_7P]^-$	227.0321 amu
		FA carboxylate anions	*species dependent*
PI	$[M-H]^-$	Cyclic inositol phosphate – H_2O $[C_6H_8O_7P]^-$	223.0008 amu
		Cyclic inositol phosphate $[C_6H_{10}O_8P]^-$	241.0113 amu
		FA carboxylate anions	*species dependent*
PS	$[M-H]^-$	Neutral loss of serine – H_2O $[C_3H_5NO_2]$	87.0320 amu
		FA carboxylate anions	*species dependent*
SM	$[M+HCOO]^-$	Neutral loss of methyl group and formate ion $[C_2H_6O_2]$	60.0211 amu
		Choline phosphate ion $[C_4H_{12}NO_4P]^-$	168.0431 amu
		FA carboxylate anions	*species dependent*
CL	$[M-H]^-$	FA carboxylate anions	*species dependent*
	$[M-H]^-$	Glycerol phosphate – H_2O $[C_3H_6O_5P]^-$	152.9953 amu

Table S4. Qualitative fragments of lipids in positive ion mode. List of different lipid classes and their qualitative fragments, in positive ESI, when MS/MS fragmentation is performed.

Lipid class	Precursor ion	Fragment	
PC	$[M+H]^+$	Phosphocholine $[C_5H_{15}NO_4P]^+$	184.0739 amu
PE	$[M+H]^+$	Neutral loss of phosphoethanolamine $[C_2H_8NO_4P]$	141.0191 amu
PG	$[M+NH_4]^+$	Neutral loss of NH_3 and phosphoglycerol $[C_3H_{12}NO_6P]$	189.0402 amu
PI	$[M+NH_4]^+$	Neutral loss of NH_3 and phosphoinositol $[C_6H_{16}NO_9P]$	277.0563 amu
PS	$[M+H]^+$	Phosphorylserine $[C_3H_8NO_6P]^+$	185.0089 amu
SM	$[M+H]^+$	Phosphocholine $[C_5H_{15}NO_4P]^+$	184.0739 amu
CL	$[M+NH_4]^+$	Neutral loss of NH_3 and phosphoglycerol $[C_3H_{12}NO_6P]$	189.0402 amu
TG	$[M+NH_4]^+$	Neutral loss of NH_3 and FA	*species dependent*
Cholesterol, CE	$[M+NH_4]^+$	Neutral loss of NH_3 and –OH or FA, resulting in $[C_{27}H_{45}]^+$	369.3521 amu

A

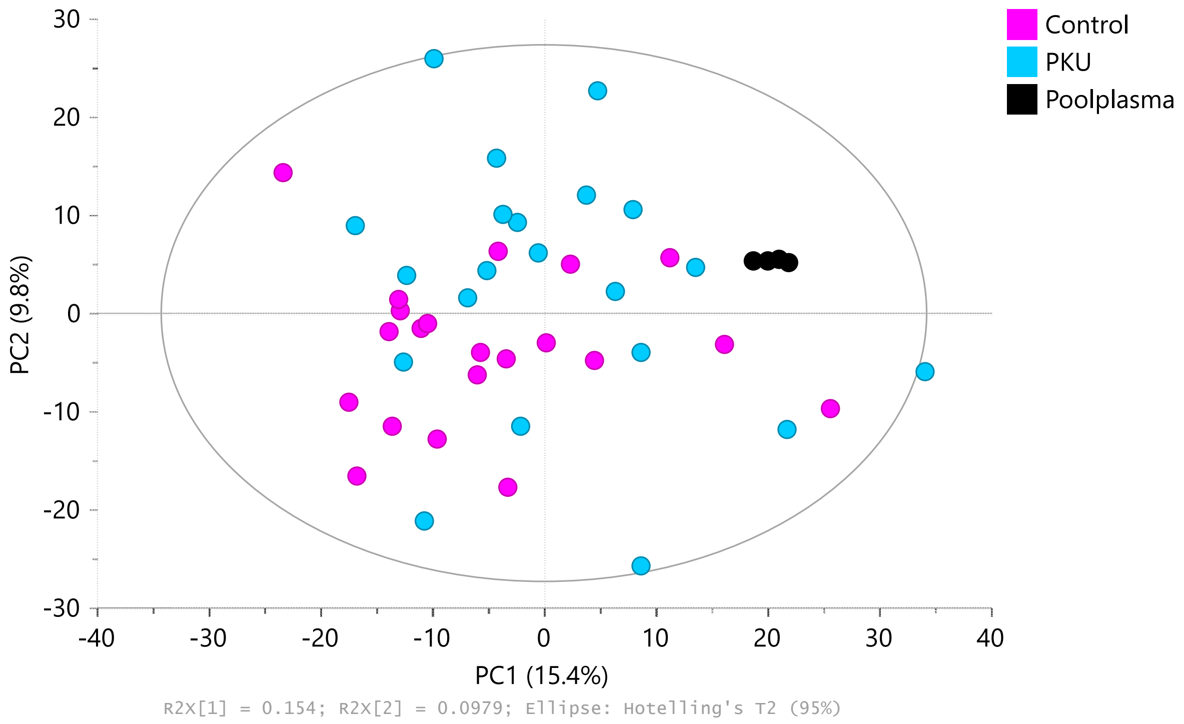

B

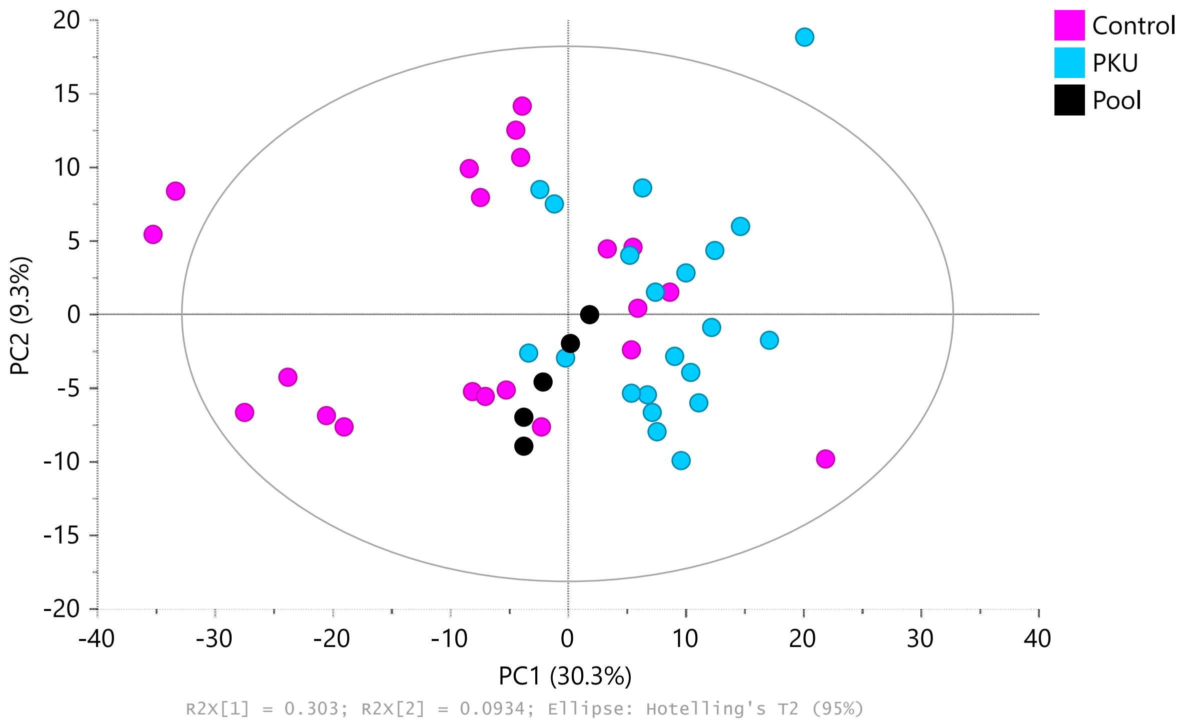

Figure S7. Two-dimensional score plot of the PCA model based on the raw data. The model included all study samples and the runs from the pooled sample (n=47) in positive (A) and negative (B) ionization mode. Samples are coloured according to group. In both figures, the first principal component (PC 1) is plotted against the second principal component (PC 2).

A

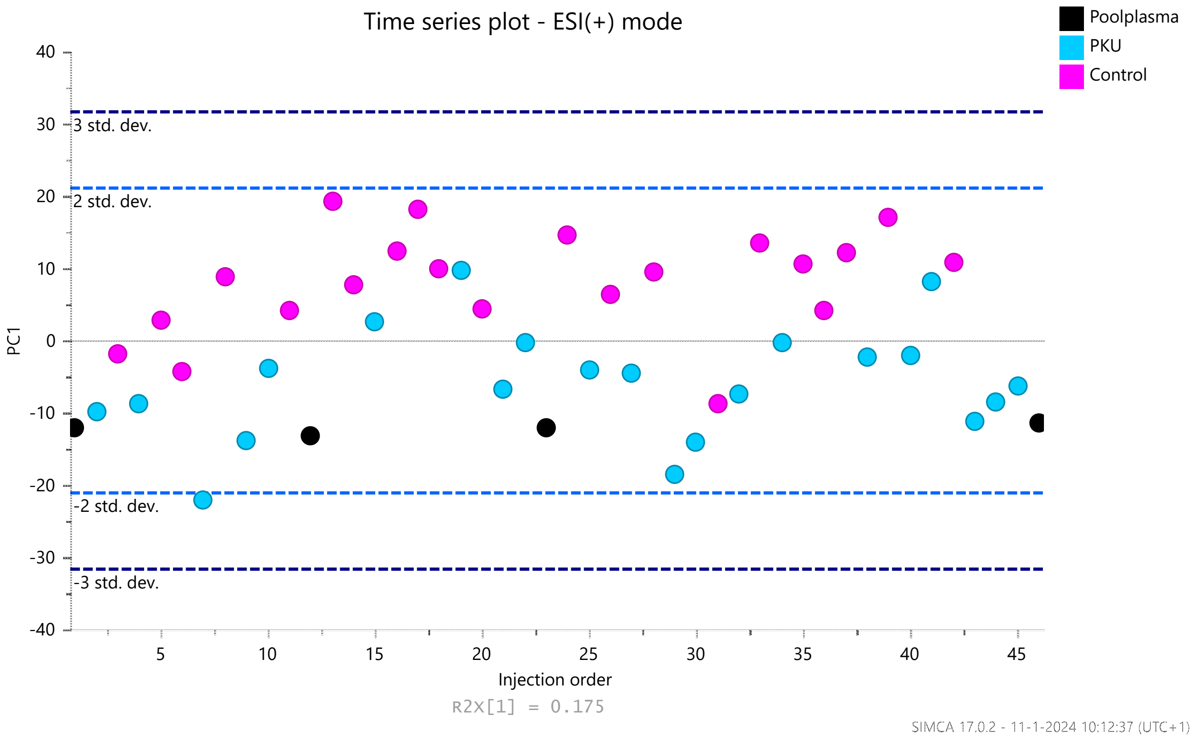

B

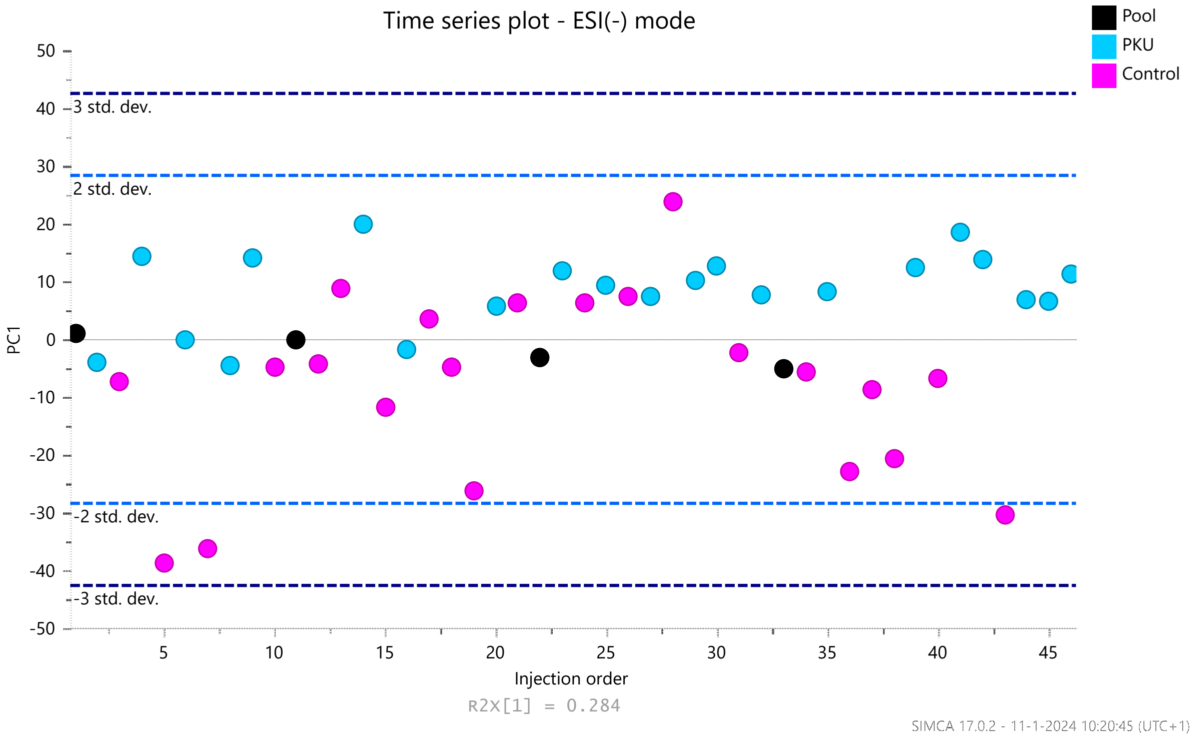

Figure S8. Time series plot of PC1 against the sample injection order in positive and negative ionization mode. The ±2SD and ±3SD limits are shown for PC1 values. Each sample type is displayed in a different color: PKU samples are shown in blue, control samples in pink and pooled sample in black. No trend was observed for the pooled samples, indicating stability of the analytical platform.

Table S5. Information of the samples included in the lipidomics and metabolomics study.

Sample ID	Type	Sample collection (year)	Age	Sex	Lifetime complete [Phe] (μmol/L)	Concurent [Phe] (μmol/L)	BH4 responsive	BH4 treatment	Lipidomics	Metabolomics
538	PKU	2013	38	Male	351	418	No	No	O	O
408	PKU	2014	31	Female	399	615	No	No	O	O
552	PKU	2013	20	Male	341	616	No	No	X	O
513	PKU	2012	19	Female	230	220	Yes	Yes	X	O
572	PKU	2014	23	Female	549	412	Unknown	No	X	O
570	PKU	2014	22	Female	527	394	Yes	Yes	X	O
577	PKU	2014	35	Male	502	763	No	No	O	O
548	PKU	2013	40	Female	734	618	No	No	O	O
467	PKU	2013	23	Male	353	471	Unknown	No	O	O
462	PKU	2013	29	Female	291	427	Yes	Yes	O	O
455	PKU	2012	24	Female	514	1250	No	No	O	O
567	PKU	2013	28	Male	337	314	No	No	O	O
466	PKU	2013	24	Female	306	259	Yes	No	X	O
524	PKU	2013	19	Male	223	317	Unknown	Yes	X	O
571	PKU	2014	19	Male	350	1030	Yes	Yes	X	O
579	PKU	2014	35	Male	-	1250	No	No	O	O
539	PKU	2013	33	Male	728	828	No	No	O	O
581	PKU	2014	22	Female	1001	884	No	No	X	O
580	PKU	2014	36	Male	554	1030	Unknown	No	O	O
569	PKU	2014	28	Female	420	570	Unknown	Yes	X	O
527	PKU	2014	33	Male	356	511	Yes	Yes	O	O
547	PKU	2013	32	Female	439	749	No	No	O	O
541	PKU	2013	32	Female	698	1010	Unknown	No	O	O
573	PKU	2014	23	Male	255	176	No	No	O	O
576	PKU	2014	43	Female	-	594	No	No	O	O
542	PKU	2013	19	Male	337	669	Yes	Yes	X	O
564	PKU	2013	39	Male	298	262	Unknown	No	O	O
582	PKU	2014	39	Male	638	671	No	No	O	O
451	PKU	2013	26	Female	446	345	Unknown	No	X	O
520	PKU	2012	37	Male	452	702	No	No	O	O
521	PKU	2012	40	Male	505	752	No	No	O	O
525	PKU	2013	33	Female	297	328	Yes	No	O	O
545	PKU	2013	21	Male	642	987	Unknown	No	O	O
414	PKU	2013	23	Female	652	777	No	No	X	O
457	PKU	2012	25	Female	547	1060	Unknown	No	X	O
1	Control	2016	30	Male	-	-	-	-	O	O
17	Control	2016	40	Female	-	-	-	-	O	O
202	Control	2017	37	Male	-	-	-	-	O	O
319	Control	2017	43	Female	-	-	-	-	O	O
358	Control	2017	24	Female	-	-	-	-	O	O
403	Control	2017	30	Male	-	-	-	-	O	O

Sample ID	Type	Sample collection (year)	Age	Sex	Lifetime complete [Phe] (µmol/L)	Concurent [Phe] (µmol/L)	BH4 responsive	BH4 treatment	Lipidomics	Metabolomics
476	Control	2018	40	Male	-	-	-	-	O	X
485	Control	2018	32	Male	-	-	-	-	O	X
527	Control	2018	34	Male	-	-	-	-	O	O
570	Control	2018	36	Female	-	-	-	-	O	O
608	Control	2018	35	Female	-	-	-	-	O	O
662	Control	2018	27	Male	-	-	-	-	O	O
673	Control	2018	34	Female	-	-	-	-	O	O
699	Control	2018	34	Female	-	-	-	-	O	O
846	Control	2019	39	Male	-	-	-	-	O	O
883	Control	2019	38	Male	-	-	-	-	O	O
884	Control	2019	36	Male	-	-	-	-	O	O
1001	Control	2019	40	Male	-	-	-	-	O	O
1074	Control	2019	30	Male	-	-	-	-	O	O
1106	Control	2020	36	Female	-	-	-	-	O	O
1189	Control	2020	27	Male	-	-	-	-	O	O
1229	Control	2020	26	Male	-	-	-	-	O	O

Table S6. Lipidomic features in ESI(+).

Feature ID	Retention time (min)	m/z	Adducts	Annotation	Score	Fragmentation Score	Mass Error (ppm)	Isotope Similarity (%)	Annotation level
1.09_460.1374n	1.09	499.1006	M+H-H2O, M+H, M+Na, M+CH3OH+H, M+K						4
1.27_386.1735n	1.27	387.1808	M+H-H2O, M+H, M+NH4, M+Na, M+K						4
1.28_375.2526n	1.28	376.2599	M+H, M+NH4, M+Na, M+K						4
1.53_638.1636n	1.53	677.127	M+H-H2O, M+Na, M+K						4
1.83_446.2537m/z	1.83	446.2537							4
2.40_608.1526n	2.4	647.116	M+H-H2O, M+H, M+NH4, M+Na, M+K, M+2Na-H						4
2.77_620.1517n	2.77	659.115	M+H, M+NH4, M+Na, M+K						4
2.98_576.1260n	2.98	594.1598	M+H, M+NH4, M+Na, M+K						4
3.23_384.1917n	3.23	423.1563	M+Na, M+K						4

Feature ID	Retention time (min)	m/z	Adducts	Annotation	Score	Fragmentation Score	Mass Error (ppm)	Isotope Similarity (%)	Annotation level
3.64_800.1948n	3.64	839.1579	M+H-H2O, M+NH4, M+Na, M+K						4
3.74_812.1948n	3.74	851.1581	M+H, M+NH4, M+Na, M+K						4
3.77_355.2727n	3.77	356.28	M+H, M+Na, M+K, M+2Na-H						4
3.93_467.3013n	3.93	468.3086	M+H, M+Na, M+K	LPC 14:0 [iso 1]	43.4	21.2	0.34	96	2
4.18_541.3176n	4.18	542.3247	M+H, M+Na, M+K	LPC 20:5 [iso 2]	39.1	0	1.33	97	2
4.25_467.3020n	4.25	468.3093	M+H-H2O, M+H, M+Na, M+K	LPC 14:0 [iso 2]	52.3	45	1.78	99	2
4.44_541.3179n	4.44	542.3252	M+H, M+Na, M+K, M+2Na-H	LPC 20:5 [iso 3]	39.3	0	1.93	99	2
4.79_768.1700n	4.79	807.1334	M+H, M+NH4, M+K						4
4.86_519.3333n	4.86	520.3411	M+H-H2O, M+H, M+Na, M+CH3OH+H, M+K, M+2Na-H, 2M+H, 2M+Na, 2M+K						4
4.88_567.3335n	4.88	568.3403	M+H-H2O, M+H, M+Na	LPC 22:6 [iso 1]	48.5	35.2	1.72	98	2
4.95_482.3250m/z	4.95	482.325	M+H	LPE 18:0	39.3	0	1.83	99	2
4.96_376.2293n	4.96	377.2366	M+H-H2O, M+H, M+Na						4
4.96_415.1927m/z	4.96	415.1927							4
5.11_543.3334n	5.11	544.3407	M+H-H2O, M+H, M+Na, M+K, M+2Na-H, 2M+Na, 2M+K	LPC 20:4	48.4	45.8	1.71	98	2
5.11_567.3333n	5.11	568.3406	M+H-H2O, M+H, M+Na, M+K, 2M+Na, 2M+K	LPC 22:6 [iso 2]	50.7	38.1	1.49	97	2
5.15_519.3337n	5.15	520.341	M+H-H2O, M+H, M+Na, M+K, 2M+H, 2M+Na, 2M+K						4
5.15_622.4327n	5.15	623.4402	M+H-H2O, M+H						4

Feature ID	Retention time (min)	m/z	Adducts	Annotation	Score	Fragmentation Score	Mass Error (ppm)	Isotope Similarity (%)	Annotation level
5.32_495.3335n	5.32	496.3408	M+H-H2O, M+H, M+Na, M+K, 2M+H, 2M+Na, 2M+K	LPC 16:0 [iso 1]	41	8.59	2.14	99	2
5.49_569.3488n	5.49	570.3561	M+H-H2O, M+H, M+NH4, M+Na, M+K	LPC 22:5	46.8	36.7	1.17	99	2
5.60_495.3332n	5.6	496.3406	M+H-H2O, M+H, M+Na, M+K, 2M+H, 2M+Na, 2M+K	LPC 16:0 [iso 2]	63.1	92.1	1.37	99	2
5.60_498.3417n	5.6	499.3489	M+H, 2M+H						4
5.60_598.4326n	5.6	599.4401	M+H-H2O, M+H						4
5.64_545.3489n	5.64	546.3562	M+H-H2O, M+H, M+Na, 2M+H, 2M+Na	LPC 20:3	57.1	76.4	1.45	95	2
5.64_531.2744m/z	5.64	531.2744	M+H	LPG 20:5	37.3	0.0767	4.99	92	2
5.81_593.3691n	5.81	594.3763	M+H, M+Na, M+K						4
5.91_624.4483n	5.91	625.4552	M+H-H2O, M+H						4
6.23_467.3016n	6.23	468.3089	M+H-H2O, M+H, M+Na, M+K, M+2Na-H	LPE 17:0	60.4	85.4	0.88	95	2
6.49_523.3640n	6.49	546.3532	M+Na, M+K, 2M+Na	LPC 18:0 [iso 1]	51	57.5	0.35	98	2
6.62_360.2341n	6.62	361.2413	M+H, M+NH4, M+Na, M+K						4
6.74_523.3646n	6.74	546.3538	M+H-H2O, M+H, M+Na, M+K, M+2Na-H, 2M+H, 2M+Na, 2M+K	LPC 18:0 [iso 2]	63.6	93.6	1.57	97	2
6.95_549.3800n	6.95	550.3873	M+H, M+Na, M+K	LPC 20:1	53.2	73.8	1	94	2
7.15_507.3692n	7.15	508.3765	M+H, M+Na	LPC O-18:1 or LPC P-18:0	48	46	0.63	95	2
7.23_537.3798n	7.23	538.387	M+H, M+Na, M+K	LPE 22:0	45.4	0	0.59	97	2
7.28_509.3850n	7.28	510.3923	M+H, M+Na, M+K	LPC O-18:0	47.1	12.4	0.9	93	2
7.66_551.3954n	7.66	552.4026	M+H, M+Na, M+K	PC O-20:0	51.3	33.8	0.51	96	2
8.16_320.3086n	8.16	321.3159	M+H-H2O, M+H						4
8.16_460.3226n	8.16	461.3299	M+H, M+Na, M+K						4

Feature ID	Retention time (min)	m/z	Adducts	Annotation	Score	Fragmentation Score	Mass Error (ppm)	Isotope Similarity (%)	Annotation level
8.27_386.3400n	8.27	409.3292	M+H-H2O, M+H, M+Na, M+K	MG 20:0	47.8	24.8	0.95	99	2
8.42_974.7121n	8.42	992.7451	M+H, M+2H, M+NH4, M+Na						4
8.44_842.6329n	8.44	860.6668	M+H, M+2H, M+NH4, M+Na, M+K						4
8.47_589.4475n	8.47	590.4548	M+H, M+Na	CerP 32:1;O2	38.3	0	0.72	92	2
8.52_916.6057n	8.52	955.5688	M+NH4, M+Na, M+K	PI 40:3	43.9	30.7	1.75	91	2
8.55_624.4030n	8.55	625.4103	M+H, M+NH4, M+Na, M+K	PG 25:0	42.3	0	4.42	93	2
8.64_440.4100m/z	8.64	440.41							4
8.84_339.3506n	8.84	340.3579	M+H, M+Na, M+K	Amide C22	51.2	46.6	1.42	99	2
8.95_672.5209n	8.95	673.5282	M+H, M+Na, M+K	SM 32:2;O2	65.2	91.1	0.39	99	2
9.01_652.4342n	9.01	653.4414	M+H, M+NH4, M+Na, M+K	PG 27:0 [iso 1]	38	0.0448	4.04	95	2
9.15_678.4415n	9.15	679.4488	M+H, M+K						4
9.27_833.5858n	9.27	856.575	M+H-H2O, M+Na, M+K	Hex2Cer 32:1;O2	48.4	45	-0.76	98	2
9.32_652.4344n	9.32	653.4416	M+H-H2O, M+H, M+NH4, M+Na, M+K	PG 27:0 [iso 2]	42.7	25.5	4.32	93	2
9.35_686.5364n	9.35	687.5437	M+H, M+Na, M+K	CerPE 36:2;O2	51.1	28.9	0.18	96	2
9.44_859.6018n	9.44	882.591	M+Na, M+K						4
9.55_674.5369n	9.55	675.5441	M+H-H2O, M+H, M+Na, M+K, M+2Na-H, 2M+H, 2M+Na	SM 32:1;O2	67.5	95.1	0.88	98	2
9.74_700.5526n	9.74	701.5599	M+H-2H2O, M+H-H2O, M+H, M+Na, M+K, M+2Na-H, 2M+H, 2M+Na, 2M+K	SM 34:2;O2	62.8	87.8	0.93	98	2
9.76_616.5068n	9.76	599.5035	M+H-H2O, M+Na, M+K, M+2Na-H	DG 36:4 [iso 1]	57.9	0	0.24	94	2
9.83_592.5070n	9.83	575.5037	M+H-H2O, M+Na, M+K	DG 34:2 [iso 1]	52.1	17.5	0.54	98	2
9.83_834.5257n	9.83	857.5149	M+Na, M+K, M+2Na-H	PI 34:2	53.8	25.2	-0.15	98	2
9.86_369.3522m/z	9.86	369.3522	M+H						4

Feature ID	Retention time (min)	m/z	Adducts	Annotation	Score	Fragmentation Score	Mass Error (ppm)	Isotope Similarity (%)	Annotation level
9.88_676.5519n	9.88	677.5592	M+H, M+Na, M+K	SM 32:0;O2	59.7	64.8	-0.02	97	2
9.90_884.5406n	9.9	907.5298	M+Na, M+K, M+2Na-H	PI 38:5	40.6	6.78	-1.02	97	2
9.93_1023.6696n	9.93	1062.6327	M+H-H2O, M+H, M+K	Hex3Cer 34:1;O2	50.7	23.1	-0.93	97	2
9.93_777.5303n	9.93	800.5204	M+Na, M+K	PC 36:6	51	47.9	-0.74	89	2
9.93_726.5667n	9.93	727.574	M+H-H2O, M+H, M+Na, M+CH3OH+H, M+K	SM 36:3;O2	53.4	57.5	-1.21	94	2
9.96_765.5293n	9.96	766.5366	M+H, M+Na	PE 38:5	42.2	17.4	-2.06	96	2
9.98_688.5523n	9.98	689.5596	M+H-H2O, M+H, M+Na, M+K, 2M+Na	SM 33:1;O2	66.2	92.5	0.59	98	2
10.03_753.5298n	10.03	754.5371	M+H, M+Na, M+K	PC 34:4	52	51.5	-1.35	91	2
10.06_691.5156n	10.06	692.5229	M+H, M+Na	PE 32:0	53.1	44.5	0.59	96	2
10.08_861.6170n	10.08	884.6066	M+H-H2O, M+H, M+Na, M+K, M+2Na-H	Hex2Cer 34:1;O2	53.3	69	-0.9	99	2
10.08_729.5313n	10.08	730.5385	M+H, M+Na, M+CH3OH+H, M+K, 2M+Na	PC 32:2	58.5	78.7	0.55	93	2
10.11_803.5465n	10.11	826.5357	M+Na, M+K	PC 38:7 [iso 1]	58	85.8	-0.05	89	2
10.18_714.5673n	10.18	715.5745	M+H, M+Na	CerPE 38:2;O2 [iso 1]	42.1	21	-0.43	90	2
10.25_853.5600n	10.25	854.5672	M+H, M+Na, M+K	PC 42:10	40.1	15.8	-2.57	88	2
10.25_486.3745n	10.25	487.3818	M+H, M+NH4, M+Na, M+K						4
10.27_699.5649n	10.27	722.5541	M+H-H2O, M+Na, M+K	HexCer 34:1;O2	54.3	61.2	-0.07	86	2
10.32_826.5356m/z	10.32	826.5356	M+Na	PC 38:7 [iso 2]	55.7	34.8	-0.2	87	2
10.35_558.4312n	10.35	559.4385	M+H, M+Na, M+K	DG 32:5	41.3	16.4	4.99	96	2
10.37_779.5469n	10.37	780.5542	M+H, M+K, 2M+H, 2M+Na, 2M+K	PC 36:5	53.6	32.4	0.54	78	2
10.40_702.5684n	10.4	703.5757	M+H-H2O, M+H, M+Na, M+H+Na, 2M+H, 2M+Na, 2M+K	SM 34:1;O2	65.4	96	1.21	99	2
10.40_184.0745m/z	10.4	184.0745							4
10.47_668.5365n	10.47	651.5348	M+H-H2O, M+Na	DG 40:6	49.6	56	-2.14	95	2
10.49_705.5313n	10.49	706.5386	M+H, M+Na, M+K	PC 30:0	50.5	30.7	0.65	98	2
10.52_541.3146n	10.52	542.3218	M+H, M+Na	LPC 20:5 [iso 1]	46.9	31.8	-4.2	91	2

Feature ID	Retention time (min)	m/z	Adducts	Annotation	Score	Fragmentation Score	Mass Error (ppm)	Isotope Similarity (%)	Annotation level
10.52_743.5460n	10.52	744.5533	M+H, M+NH4, M+Na, M+K, 2M+Na, 2M+K	PC 33:2	38.5	7.05	-0.69	86	2
10.52_781.5604n	10.52	782.5698	M+H, 2M+H, 2M+Na, 2M+K	PC 36:4	56.3	68.6	-2.21	98	2
10.54_644.5385n	10.54	627.5352	M+H-H2O, M+Na, M+K	DG 38:4	51.8	61.3	0.79	99	2
10.54_886.5561n	10.54	909.5453	M+H-H2O, M+Na, M+K	PI 38:4	62.6	41.4	-1.15	98	2
10.60_728.5835n	10.6	729.5908	M+H-H2O, M+H, M+Na, M+2Na-H, 2M+H, 2M+Na	SM 36:2;O2	58.3	74.7	0.39	93	2
10.67_731.5470n	10.67	732.5543	M+H, M+Na, M+K, 2M+H, 2M+Na, 2M+K	PC 32:1	56	62.3	0.73	98	2
10.69_670.5536n	10.69	653.5503	M+H-H2O, M+Na, M+K	DG 40:5	45.8	34.3	0.02	95	2
10.69_769.5616n	10.69	770.5689	M+H, M+K	PE 38:3	43.2	22.6	-0.7	94	2
10.71_716.5827n	10.71	717.5899	M+H, M+Na	SM 35:1;O2 [iso 1]	44.3	15.8	-0.79	91	2
10.74_704.5831n	10.74	705.5906	M+H, M+Na	SM 34:0;O2	49.1	21.4	-0.17	97	2
10.76_908.6610n	10.76	891.6577	M+H-H2O, M+H						4
10.84_716.5831n	10.84	717.5904	M+H, M+Na	SM 35:1;O2 [iso 2]	46.1	10.6	-0.13	91	2
10.84_839.5204n	10.84	872.5382	M+Na, M+CH3OH+H						4
11.01_592.5083n	11.01	625.5188	M+Na, M+CH3OH+H	DG 34:2 [iso 2]	38.9	0.0585	2.69	98	2
11.03_694.4958m/z	11.03	694.4958							4
11.10_746.5694m/z	11.1	746.5694	M+H	PE 36:1	38.8	1.44		92	2
11.27_717.5661n	11.27	718.5734	M+H, M+Na	PC O-32:1 or PC P-32:0 [iso 1]	41.1	13.3	-1.59	94	2
11.30_795.5765n	11.3	796.5837	M+H, M+K	PE 40:4	56.7	54.2	-1.68	97	2
11.37_733.5622n	11.37	734.5695	M+H, M+Na, M+K, 2M+H, 2M+Na, 2M+K	PC 32:0	53.6	48.2	0.08	98	2
11.40_767.5826n	11.4	768.5899	M+H, M+Na, 2M+H	PC O-36:4 or PC P-36:3	54.9	60.5	-0.35	96	2
11.42_817.5971n	11.42	818.6044	M+H, M+Na	PC P-40:6	44.9	29.6	-1.7	97	2
11.47_744.5898m/z	11.47	744.5898	M+H	PC O-34:2 or PC P-34:1	40.8	6.48	-0.5	98	2

Feature ID	Retention time (min)	m/z	Adducts	Annotation	Score	Fragmentation Score	Mass Error (ppm)	Isotope Similarity (%)	Annotation level
11.47_756.6139n	11.47	757.6211	M+H-H2O, M+H, M+Na	SM 38:2;O2	52	41.3	-0.88	98	2
11.54_759.5778n	11.54	760.5851	M+H, 2M+H, 2M+Na	PC 34:1	61.9	96	0.02	97	2
11.55_717.5326n	11.55	718.5399	M+H, M+Na	PE 34:1	41.7	20	2.49	91	2
11.60_616.5066n	11.6	617.5137	M+H-H2O, M+H, M+NH4, M+Na, M+K	DG 36:4 [iso 2]	39.2	0.716	-0.14	95	2
11.62_833.5929n	11.62	834.6001	M+H-H2O, M+H, M+Na, M+K, 2M+H, 2M+Na, 2M+K	PC 40:6	54.5	60.9	-0.71	98	2
11.66_528.4212n	11.66	529.4285	M+H, M+NH4, M+Na, M+K						4
11.67_360.1796n	11.67	361.1869	M+H, M+CH3OH+H						4
11.67_569.3674m/z	11.67	569.3674							4
11.74_859.6063n	11.74	860.6136	M+H, M+Na	PC 42:7	42.8	29.9	-3.24	88	2
11.76_717.5669n	11.76	718.5742	M+H, M+Na	PC O-32:1 or PC P-32:0 [iso 2]	49.9	38.3	-0.45	91	2
11.76_609.5007n	11.76	627.5346	M+NH4, M+CH3OH+H						4
11.88_835.6085n	11.88	836.6158	M+H, M+Na, M+K, 2M+H, 2M+Na	PC 40:5	55.4	69.6	-0.68	93	2
11.90_720.5895m/z	11.9	720.5895	M+H	PC O-32:0	45.6	31.5	-0.88	97	2
11.98_592.5048n	11.98	615.4959	M+H-H2O, M+H, M+Na, M+K, M+2Na-H	DG 34:2 [iso 3]	39.7	3.14	-3.09	99	2
12.04_745.5978n	12.04	746.6051	M+H, M+Na	PC O-34:1 or PC P-34:0	55.3	64.4	-0.97	96	2
12.08_811.6094n	12.08	812.6167	M+H, M+Na, M+K, 2M+H, 2M+Na, 2M+K	PC 38:3	57.2	73.6	0.42	97	2
12.13_751.5515n	12.13	752.5587	M+H, M+K, M+2Na-H	PE O-38:5 or PE P-38:4	44	6.39	-0.16	99	2
12.15_758.6304n	12.15	759.6377	M+H-H2O, M+H, M+Na, M+K, 2M+H, 2M+Na	SM 38:1;O2	54.8	58.1	0.32	96	2
12.18_861.6227n	12.18	862.6293	M+H, M+Na, M+K	PC 42:6	43.1	23.9	-2.35	94	2
12.20_618.5223n	12.2	601.5193	M+H-H2O, M+H, M+NH4, M+Na, M+K	DG 36:3	39.6	1.07	-0.09	97	2

Feature ID	Retention time (min)	m/z	Adducts	Annotation	Score	Fragmentation Score	Mass Error (ppm)	Isotope Similarity (%)	Annotation level
12.25_765.5657n	12.25	766.6117	M+H, 2M+NH4	PC O-36:5 or PC P-36:4	38.8	2.56	-1.99	94	2
12.25_795.6141n	12.25	796.6214	M+H-H2O, M+H, M+Na	PC O-38:4 or PC P-38:3	40.5	8.61	-0.12	94	2
12.39_787.6096n	12.39	788.6169	M+H, M+K, 2M+H, 2M+Na, 2M+K	PC 36:1	62	95.1	0.64	99	2
12.44_810.6617n	12.44	811.6689	M+H-H2O, M+H, M+Na, 2M+Na	SM 42:3;O2	60.1	87.2	0.23	96	2
12.57_772.6453n	12.57	773.6525	M+H-H2O, M+H, M+Na, 2M+Na	SM 39:1;O2	50.7	40.6	-0.72	87	2
12.60_945.7104n	12.6	968.6996	M+H-H2O, M+Na, M+K	Hex2Cer 40:1;O2	46.3	43.1	-1.34	90	2
12.69_971.7265n	12.69	994.7157	M+H-H2O, M+Na, M+K	Hex2Cer 42:2;O2	52.1	47.6	-0.81	97	2
12.76_747.6141n	12.76	748.6214	M+H-H2O, M+H, M+Na, M+CH3OH+H	PC O-34:0	60.6	92.6	-0.11	93	2
12.78_620.5376n	12.78	643.5278	M+H-H2O, M+H, M+NH4, M+Na, M+K, M+2Na-H	DG 36:2	61.7	32.6	-0.59	98	2
12.81_783.6585n	12.81	806.6478	M+H-H2O, M+Na	HexCer 40:1;O2	48.4	31.3	-0.43	95	2
12.81_645.6061n	12.81	668.5953	M+Na, M+K						4
12.90_809.6510n	12.9	774.6371	M+H-2H2O, M+H-H2O						4
12.98_786.6622n	12.98	787.6695	M+H-H2O, M+H, M+Na, M+H+Na, M+K, 2M+H, 2M+Na	SM 40:1;O2	60.9	91.1	0.91	97	2
12.98_592.5911n	12.98	593.5984	M+H, M+Na, M+K						4
13.22_646.4526n	13.22	647.4599	M+H, M+Na, M+K						4
13.40_800.6774n	13.4	801.6847	M+H-2H2O, M+H-H2O, M+H, M+NH4, M+Na, M+K, 2M+H, 2M+Na	SM 41:1;O2	61.9	94.6	0.32	97	2
13.57_775.6449n	13.57	776.6522	M+H, M+Na	PC O-36:0	52.4	49.4	-0.77	97	2
13.60_793.6793n	13.6	794.6866	M+H, M+K						4
13.79_814.6935n	13.79	815.7007	M+H-H2O, M+H, M+Na, M+K, M+2Na-H, 2M+H, 2M+Na	SM 42:1;O2	62.1	96.6	0.84	98	2

**Feature ID**	**Retention time (min)**	**m/z**	**Adducts**	**Annotation**	**Score**	**Fragmentation Score**	**Mass Error (ppm)**	**Isotope Similarity (%)**	**Annotation level**
13.90_598.4998n	13.9	621.4893	M+H, M+NH4, M+Na, M+K						4
13.98_843.6717n	13.98	844.679	M+H, M+Na, M+K	PC 40:1	38.5	8.09	0.04	84	2
14.16_649.6367n	14.16	672.6271	M+H-2H2O, M+H-H2O, M+H, M+Na, M+K, M+2Na-H, 2M+Na	Cer 42:1;O2	42.7	16.8	-0.88	98	2
14.22_869.6867n	14.22	870.694	M+H, M+Na, M+K	PC 42:2	42.7	20.8	-0.76	94	2
14.25_879.7072n	14.25	880.7145	M+H, M+Na, M+K	PC O-44:4	50.7	58.2	-1.03	96	2
14.62_714.5790n	14.62	737.5526	M+NH4, M+Na, M+K	TG 42:4	41.6	15.1	-1.15	94	2
14.64_698.5886n	14.64	699.5959	M+H-H2O, M+H, M+Na						4
14.72_871.7027n	14.72	872.71	M+H, M+Na	PC 42:1	38.2	1.95	-0.3	90	2
15.15_694.6109n	15.15	712.6451	M+NH4, M+Na, M+K	TG 40:0	44.7	26.3	-0.28	98	2
15.62_754.6147n	15.62	772.6485	M+H, M+NH4, M+Na, M+K	TG 45:5	39.2	7.74	4.73	94	2
15.84_722.6425n	15.84	740.6763	M+NH4, M+Na	TG 42:0	42.9	16.9	0.06	98	2
15.96_748.6582n	15.96	766.692	M+H, M+NH4, M+Na	TG 44:1	44.9	25.8	0.11	99	2
16.10_774.6723n	16.1	792.7075	M+NH4, M+Na, M+CH3OH+H, M+K	TG 46:2	45.5	31	-1.83	99	2
16.16_348.3091n	16.16	714.6426	M+Na, 2M+NH4						4
16.16_430.3120n	16.16	413.3087	M+H-H2O, M+H						4
16.16_444.3276n	16.16	445.3348	M+H, M+NH4, M+Na						4
16.16_682.5942n	16.16	683.6014	M+H, M+NH4, M+Na, M+K						4
16.23_800.6895n	16.23	818.7233	M+H, M+NH4, M+Na, M+K	TG 48:3	55.6	85.4	0.07	93	2
16.27_644.5530n	16.27	667.5422	M+Na, M+K						4
16.42_926.7356n	16.42	944.7692	M+NH4, M+Na, M+K	TG 58:10	50.2	54	-0.84	98	2
16.55_714.5661n	16.55	737.5545	M+H, M+NH4, M+Na, M+K	CerPE 38:2;O2 [iso 2]	45.5	34.6	-2.1	96	2
16.57_750.6737n	16.57	768.7075	M+NH4, M+Na, M+K	TG 44:0	49.2	47.6	-0.11	99	2

Feature ID	Retention time (min)	m/z	Adducts	Annotation	Score	Fragmentation Score	Mass Error (ppm)	Isotope Similarity (%)	Annotation level
16.67_776.6893n	16.67	794.7232	M+H, M+NH4, M+Na, M+K	TG 46:1	53.5	68.2	-0.06	99	2
16.74_878.7355n	16.74	896.7693	M+NH4, M+Na, M+K	TG 54:6	49.5	53	-0.93	96	2
16.84_646.5692n	16.84	669.5585	M+H, M+NH4, M+Na, M+K						4
16.91_696.5844n	16.91	719.5738	M+H, M+NH4, M+Na, M+K						4
16.93_928.7511n	16.93	946.7849	M+NH4, M+Na, M+K	TG 58:9	51.4	61.3	-0.96	97	2
17.20_336.2925n	17.2	695.5742	M+CH3OH+H, 2M+H, 2M+NH4, 2M+Na, 2M+K						4
17.34_698.5987n	17.34	721.5879	M+Na, M+K	CE 22:5	37.8	0.936	-2.1	90	2
17.37_778.7045n	17.37	796.7383	M+NH4, M+Na, M+K	TG 46:0	49.9	51.4	-0.73	99	2
17.42_904.7514n	17.42	922.7852	M+NH4, M+Na, M+K	TG 56:7	51.2	66	-0.69	91	2
17.49_648.5845n	17.49	671.5743	M+H, M+NH4, M+Na, M+K	CE 18:2	41.9	11.7	-0.1	98	2
17.49_804.7206n	17.49	822.7544	M+H, M+NH4, M+Na, M+K	TG 48:1	46.8	35.3	-0.08	99	2
17.62_830.7357n	17.62	848.7696	M+H, M+NH4, M+Na, M+K	TG 50:2	53.4	71.4	-0.72	96	2
17.67_880.7515n	17.67	898.7854	M+NH4, M+Na, M+K	TG 54:5	42	20.1	-0.51	91	2
17.84_856.7516n	17.84	895.7147	M+H-H2O, M+H, M+K	TG 52:3	56.9	89.8	-0.47	95	2
17.98_882.7672n	17.98	900.8011	M+H, M+NH4, M+Na, M+K	TG 54:4	58.9	97	-0.46	98	2
18.11_844.7514n	18.11	862.7852	M+H, M+NH4, M+Na, M+K	TG 51:2	51.5	64.3	-0.71	94	2
18.22_624.5846n	18.22	647.5738	M+Na, 2M+Na	CE 16:0	40.4	2.9	0.12	99	2
18.30_870.7668n	18.3	888.8006	M+NH4, M+Na, M+K	TG 53:3	45.6	31.7	-0.99	98	2
18.34_806.7358n	18.34	824.7696	M+NH4, M+Na, M+K	TG 48:0	41.2	7.8	-0.69	99	2
18.37_650.6005n	18.37	673.5897	M+H, M+Na, M+K	CE 18:1	41.4	9.88	0.48	98	2
18.39_932.7818n	18.39	950.8156	M+NH4, M+Na, M+K	TG 58:7	43.4	28.9	-1.59	90	2
18.47_832.7509n	18.47	850.7858	M+H-H2O, M+H, M+NH4, M+Na, M+K, 2M+Na	TG 50:1	57.1	88.8	-1.31	99	2

Feature ID	Retention time (min)	m/z	Adducts	Annotation	Score	Fragmentation Score	Mass Error (ppm)	Isotope Similarity (%)	Annotation level
18.49_896.7831n	18.49	914.8169	M+NH4, M+Na	TG 55:4	50.3	59.4	-0.27	92	2
18.62_890.8159m/z	18.62	890.8159	M+NH4	TG 53:2	46.1	35	-1.4	97	2
18.64_858.7677n	18.64	876.8016	M+H-H2O, M+H, M+NH4, M+Na, 2M+Na	TG 52:2	57.7	91.1	0.12	97	2
18.69_908.7825n	18.69	926.8163	M+NH4, M+Na, M+K	TG 56:5	52.5	65.3	-0.91	98	2
18.79_884.7832n	18.79	902.817	M+H, M+NH4, M+Na, M+K	TG 54:3	58.6	95.6	-0.13	98	2
19.06_846.7668n	19.06	864.8006	M+NH4, M+Na, M+K	TG 51:1	45.2	30	-0.98	97	2
19.52_834.7670n	19.52	852.8008	M+NH4, M+Na, M+K	TG 50:0	50.7	56.1	-0.78	98	2
19.67_860.7830n	19.67	878.8168	M+H, M+NH4, M+Na, M+K	TG 52:1	57.9	91.3	-0.35	99	2
19.84_886.7988n	19.84	904.8326	M+H, M+NH4, M+Na, M+K	TG 54:2	59.1	97.9	-0.18	98	2
19.98_912.8140n	19.98	930.8478	M+H, M+NH4, M+Na, M+K	TG 56:3	48.3	48.5	-0.66	94	2
21.11_888.8140n	21.11	906.8478	M+NH4, M+Na, M+K	TG 54:1	58.8	96.4	-0.65	98	2
21.30_914.8295n	21.3	932.8633	M+NH4, M+Na, M+K	TG 56:2	53.7	71.6	-0.86	98	2
22.86_916.8453n	22.86	939.8346	M+NH4, M+Na, M+K	TG 56:1	43.5	24.2	-0.69	94	2

Table S7. Lipidomic features in ESI(-).

Feature ID	Retention time (min)	m/z	Adducts	Annotation	Score	Fragmentation Score	Mass Error (ppm)	Isotope Similarity	Annotation level	Isotope Distribution	Normalised on
5.13_564.3298 m/z	5.13	564.3298	M+FA-H	LPC 18:2	46.5	41.3	-1.71	93	2	100 - 32.1 - 7.79 - 5.79	LPC 17:1
5.13_588.3289 m/z	5.13	588.3289	M+FA-H	LPC 20:4	42.1	25.2	-3.3	89	2	100 - 41.6 - 11.7 - 1.78	LPC 17:1
5.15_524.2772 m/z	5.15	524.2772	M-H	LPE 22:6	38.8	5.6	-1.94	90	2	100 - 34.2 - 8.91 - 8.28	LPE 17:1
5.18_476.2769 m/z	5.18	476.2769	M-H	LPE 18:2	38.6	0	-2.93	97	2	100 - 29.3 - 5.4	LPE 17:1
5.27_277.2168 m/z	5.27	277.2168	M-H	FA 18:3	39.4	2.5	-1.72	96	2	100 - 19.6 - 6.41 - 0.413	Average
5.57_497.2877 m/z	5.57	497.2877	M-H	LPG 17:0	42.1	17.1	-1.65	95	2	100 - 23.8 - 8.17 - 1.7	LPG 17:0
5.59_480.3093 m/z	5.59	480.3093	M-H	LPE 18:0	43.6	21.8	-0.6	97	2	100 - 28.5 - 5.68 - 1.45	LPE 17:1
5.60_540.3305 m/z	5.6	540.3305	M+FA-H	LPC 16:0	51.8	60.5	-0.45	99	2	100 - 27.5 - 4.55 - 0.956 - 0.209	LPC 17:1
5.64_590.3439 m/z	5.64	590.3439	M+FA-H	LPC 20:3	40.8	22.8	-4.48	87	2	100 - 42.5 - 15.5	LPC 17:1
5.83_327.2324 m/z	5.83	327.2324	M-H	DHA	39.9	4.88	-1.81	97	2	100 - 20.7 - 3.61	Average
5.91_436.2544 n	5.91	473.2818	M-H2O-H, M-H, M+Na-2H, M+K-2H						4	100 - 26 - 3.83 - 0.405	Average
5.93_303.2329 m/z	5.93	303.2329	M-H	FA 20:4	40.1	2.56	-0.15	98	2	100 - 20.9 - 3.69 - 0.38	Average
5.98_302.2212 n	5.98	347.2194	M-H, M+FA-H, 2M+FA-H						4	100 - 22.5 - 9.16	Average
6.30_329.2478 m/z	6.3	329.2478	M-H	FA 22:5	36.7	10.7	-2.58	76	2	100 - 25.7 - 41.9	Average
6.44_256.2395 n	6.44	255.233	M-H2O-H, M-H	FA 16:0	39.2	0.604	-2.92	99	2	100 - 19 - 1.64 - 0.129	Average
6.72_312.2888 n	6.72	349.2361	M+Na-2H, M+K-2H						4	100 - 20 - 5.7 - 1.18	Average
6.74_508.3396 m/z	6.74	508.3396	M-H	LPC 17:0	45.8	42.1	-2.47	90	2	100 - 38.4 - 9.54	LPC 17:1
7.01_269.2477 m/z	7.01	269.2477	M-H	FA 17:0	39.8	3.81	-3.2	99	2	100 - 19.4 - 1.38	Average
7.27_808.5688 m/z	7.27	808.5688	M-H	IPC 36:0;O2	38.1	0	-2.61	94	2	100 - 52.6 - 15.6 - 5.11	Average
7.69_465.3036 m/z	7.69	465.3036	M-H	ST 27:1;O;S	39.3	1.77	-1.82	97	2	100 - 27.4 - 10.5 - 2.02	Average
8.47_913.5791 m/z	8.47	913.5791	M+Na-2H	PI 38:1	36.7	0.169	0.41	84	2	100 - 61.2	Average
8.52_916.6020 n	8.52	915.5947	M-H, M+Na-2H, M+FA-H	PI 40:3 [iso 1]	40	5.52	-2.29	97	2	100 - 57.5 - 18.3 - 3.7 - 1.08	Average
9.27_832.5752 m/z	9.27	832.5752	M-H	Hex2Cer 32:1;O2	39.5	9.43	-4.71	93	2	100 - 54.9 - 11.5 - 5.56	Average
9.55_719.5314 m/z	9.55	719.5314	M+FA-H	SM 32:1;O2	46.4	43.8	-4.59	93	2	100 - 40.6 - 14.3 - 6.21	Average
9.74_857.5147 m/z	9.74	857.5147	M-H	PI 36:4	41.6	17.6	-4.45	95	2	100 - 46.3 - 15	Average
9.79_833.5153 m/z	9.79	833.5153	M-H	PI 34:2	50.3	62	-3.88	94	2	100 - 45.5 - 18.9 - 4.28 - 2.02	Average
9.88_916.6022 n	9.88	915.5949	M-H, M+Na-2H	PI 40:3 [iso 2]	44.4	30.4	-2.07	94	2	100 - 60.6 - 21.6 - 4.13	Average
9.88_969.6053 m/z	9.88	969.6053	M+FA-H	PI O-42:6	41.5	21.6	-2.27	89	2	100 - 66.6 - 25.7	Average

10.08_774.5256m/z	10.08	774.5256	M+FA-H	PC 32:2	42.3	24.7	-4.79	92	2	100 - 53.3 - 13.6	PC 34:0
10.27_698.5543m/z	10.27	698.5543	M-H	HexCer 34:1;O2	43.2	26	-4.78	96	2	100 - 49 - 13.3 - 2.99	Average
10.32_835.5308m/z	10.32	835.5308	M-H	PI 34:1	43.6	27.1	-4.12	95	2	100 - 50.3 - 18.5 - 2.48 - 0.869	Average
10.37_824.5412m/z	10.37	824.5412	M+FA-H	PC 36:5	40	16.6	-4.49	88	2	100 - 52.2	PC 34:0
10.40_687.5422m/z	10.4	687.5422	M-H	CerPE 36:1;O2	50.9	62.5	-3.5	96	2	100 - 40.5 - 9.65 - 2.95 - 2.12	Average
10.40_661.5027n	10.4	642.4849							4	100 - 41.2 - 9.8 - 3.75 - 0.996	Average
10.42_909.5453m/z	10.42	909.5453	M+Na-2H	PI 38:3	40.1	10.3	-2.36	93	2	100 - 51.3 - 20.3 - 7.06 - 4.41	Average
10.52_848.5764n	10.52	885.5468							4	100 - 48.9 - 13.6 - 3.06 - 0.756 - 0.184	Average
10.67_911.5596m/z	10.67	911.5596	M+Na-2H	PI 38:2	41	20.3	-3.9	89	2	100 - 57.7 - 26.2	Average
10.76_805.5592n	10.76	850.5574	M+FA-H, 2M+FA-H						4	100 - 51.2 - 17.8 - 4.58 - 1.2	Average
10.77_537.5099n	10.77	582.5078	M-H, M+FA-H	Cer 34:1;O2	38.4	3.27	-4.07	93	2	100 - 45.3 - 10.4 - 0.708	Average
10.86_781.5593n	10.86	826.5575	M+FA-H, 2M+FA-H	PC 36:4	46.8	39.9	-3.67	98	2	100 - 48.4 - 13.2 - 3.39 - 1.72	PC 34:0
10.93_757.5599n	10.93	802.5581	M-H, M+FA-H, 2M+FA-H	PC 34:2	54	74.1	-2.98	99	2	100 - 46.6 - 12.5 - 2.94 - 0.585 - 0.0338	PC 34:0
10.93_743.5436n	10.93	742.5363	M-H, M+FA-H	PE 36:2	55	81.5	-3.9	98	2	100 - 46.6 - 10.6 - 3.11	PE 34:0
11.09_863.5613m/z	11.09	863.5613	M-H	PI 36:1	41.3	26.1	-4.87	86	2	100 - 56.8 - 25.4 - 8.62 - 5.79	Average
11.16_814.4983m/z	11.16	814.4983	M+Na-2H	PS O-38:6	38.3	19.8	-2.64	75	2	100 - 55.2 - 58.9	PS 34:0
11.18_582.5319n	11.18	581.5246	M-H, M+Na-2H						4	100 - 38 - 9.34 - 3.65 - 1.04 - 0.247	Average
11.53_759.5752n	11.53	804.5734	M+FA-H, 2M+FA-H	PC 34:1	48.7	48.7	-3.42	99	2	100 - 47.1 - 13 - 3.01 - 0.806 - 0.00647	PC 34:0
11.62_833.6073n	11.62	878.5873	M-H2O-H, M+FA-H						4	100 - 56.4 - 23.6 - 5.34	Average
11.72_809.5901n	11.72	854.5883	M+FA-H, 2M+FA-H	PC 38:4	46.1	36.3	-4.1	99	2	100 - 49.9 - 14.7 - 2.78 - 0.366	PC 34:0
11.79_785.5897n	11.79	830.5879	M+FA-H, 2M+FA-H	PC 36:2	50.2	59	-4.73	98	2	100 - 49.4 - 12.3 - 3.13 - 2.73	PC 34:0
11.88_880.6021m/z	11.88	880.6021	M+FA-H	Hex2Cer 32:0;O2	44.3	36.2	2.16	88	2	100 - 55 - 24.3 - 10.7	Average
12.08_811.6053n	12.08	856.6035	M+FA-H, 2M+FA-H	PC 38:3	44.9	33.1	-4.75	97	2	100 - 51.4 - 16.8 - 5.52 - 0.00946	PC 34:0
12.09_564.5560n	12.09	609.5558	M-H, M+FA-H						4	100 - 40.2 - 9.68 - 1.99 - 0.19 - 0.00658	Average
12.39_787.6072n	12.39	832.6054	M+FA-H, 2M+FA-H	PC 36:1	44.4	29	-2.47	96	2	100 - 48.6 - 15.2 - 5.12 - 2.41	PC 34:0
12.44_810.6578n	12.44	855.656	M+FA-H, 2M+FA-H	SM 42:3;O2	44.2	38.8	-4.55	87	2	100 - 53.4 - 17.2 - 16.8 - 8.32	Average
12.53_593.5720n	12.53	638.5699	M-H, M+FA-H	Cer 38:1;O2	38.8	4.84	-4.59	94	2	100 - 48 - 11.2 - 2.84 - 0.361 - 0.257 - 0.0474	Average
12.69_971.7253n	12.69	970.718	M-H, M+FA-H	Hex2Cer 42:2;O2	43.9	34.3	-2.08	87	2	100 - 48.3 - 11.3 - 4.24	Average
12.98_701.5730n	12.98	700.5606	M-H2O-H, M-H	CerP 40:1;O2	39.7	16.1	1.02	84	2	100 - 57.8 - 17.8 - 8.73 - 4.36	Average
12.98_592.5871n	12.98	637.5869	M-H, M+FA-H						4	100 - 48.9 - 13.7 - 2.58 - 0.458	Average
13.08_752.5923m/z	13.08	752.5923							4	100 - 51.2 - 21.9 - 8.65 - 2.46	Average

13.40_785.6497m/z	13.4	785.6497							4	100 - 53 - 18.9 - 3.46	Average
13.60_810.6791m/z	13.6	810.6791	M-H	HexCer 42:1;O2	51.1	64.4	-4.64	96	2	100 - 59.4 - 15.9 - 4.04 - 0.431	Average
13.79_859.6878m/z	13.79	859.6878	M+FA-H	SM 42:1;O2	49	51.8	-3.96	98	2	100 - 53.7 - 15.4 - 4.81	Average
13.93_922.6915m/z	13.93	922.6915	M+FA-H	PC O-44:5	43.7	29.2	1	90	2	100 - 50 - 9.54 - 3.27	PC 34:0

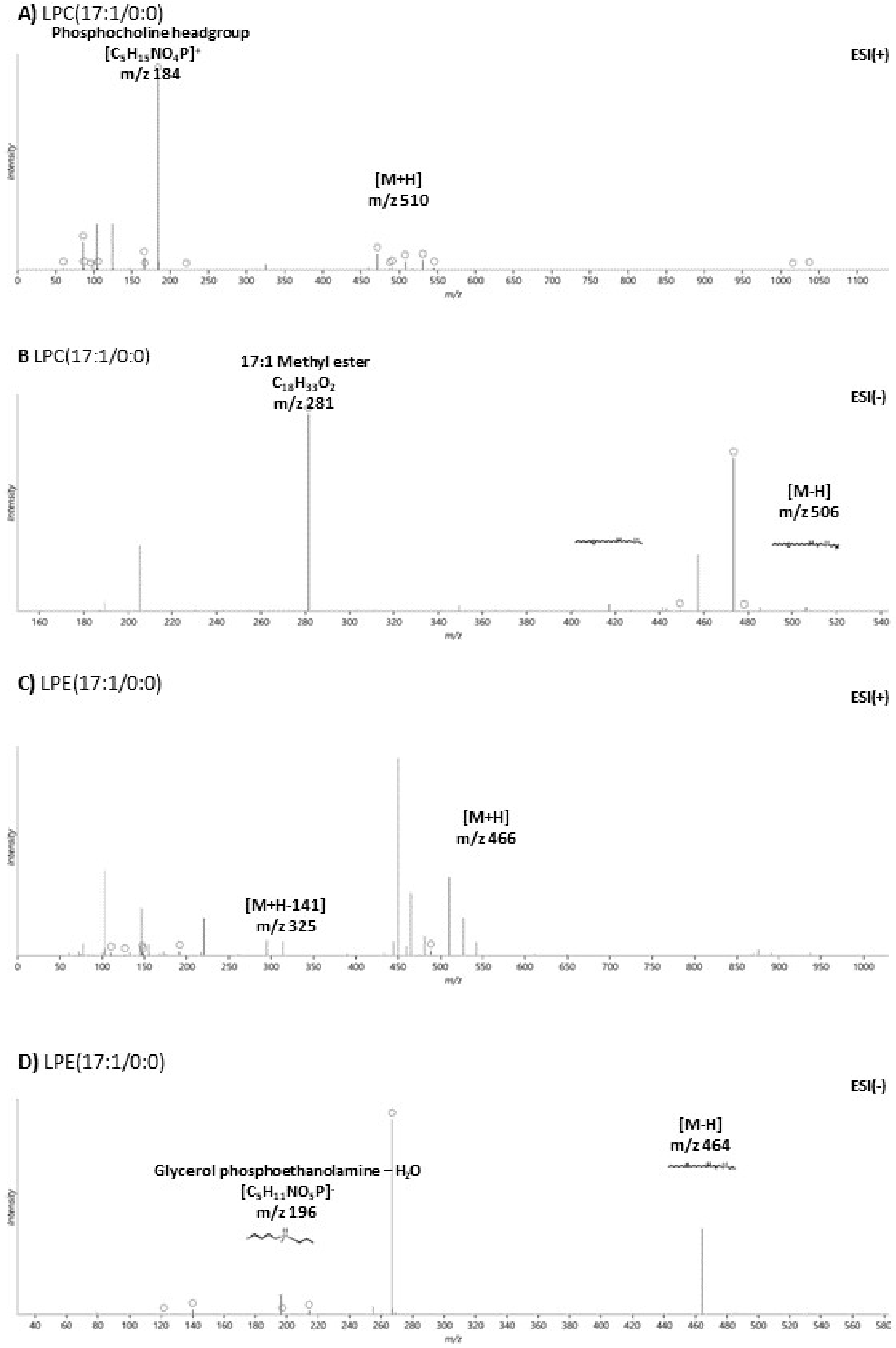
A) LPC(17:1/0:0)
Phosphocholine headgroup
[C5H15NO4P]+
m/z 184
ESI(+)
[M+H]
m/z 510
B LPC(17:1/0:0)
17:1 Methyl ester
C18H33O2
m/z 281
ESI(-)
[M-H]
m/z 506
C) LPE(17:1/0:0)
ESI(+)
[M+H]
m/z 466
[M+H-141]
m/z 325
D) LPE(17:1/0:0)
ESI(-)
[M-H]
m/z 464
Glycerol phosphoethanolamine – H2O
[C5H11NO5P]-
m/z 196
Intensity
m/z

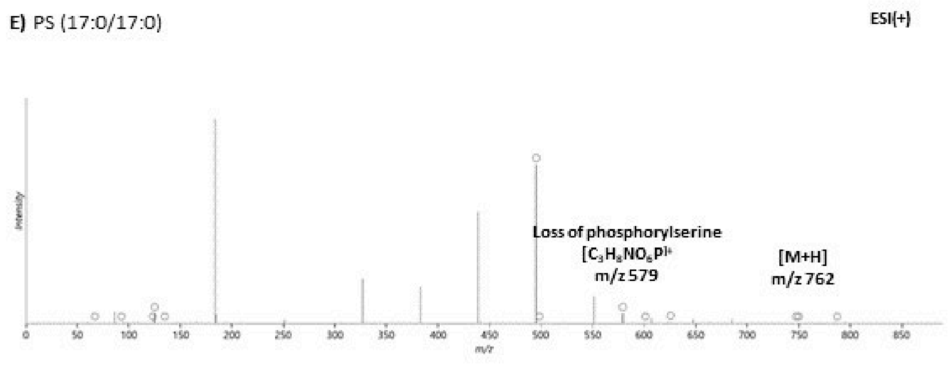
E) PS (17:0/17:0)
ESI(+)
Loss of phosphorylserine
$[C_3H_8NO_6P]^+$
m/z 579
[M+H]
m/z 762
Intensity
m/z

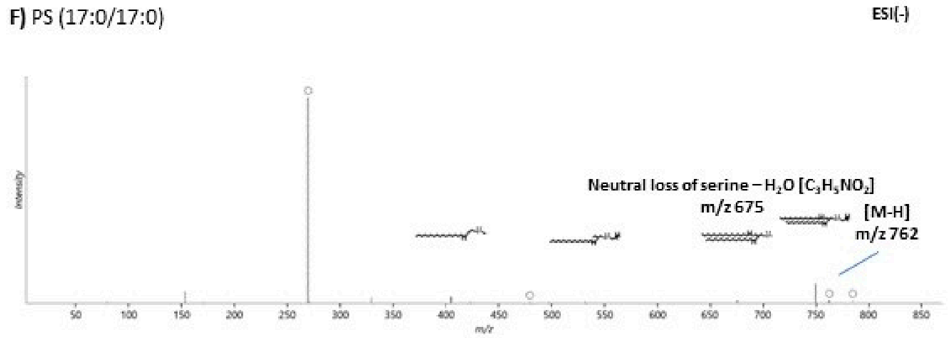
F) PS (17:0/17:0)
ESI(-)
Neutral loss of serine − H_2O $[C_3H_5NO_2]$
m/z 675
[M-H]
m/z 762
Intensity
m/z

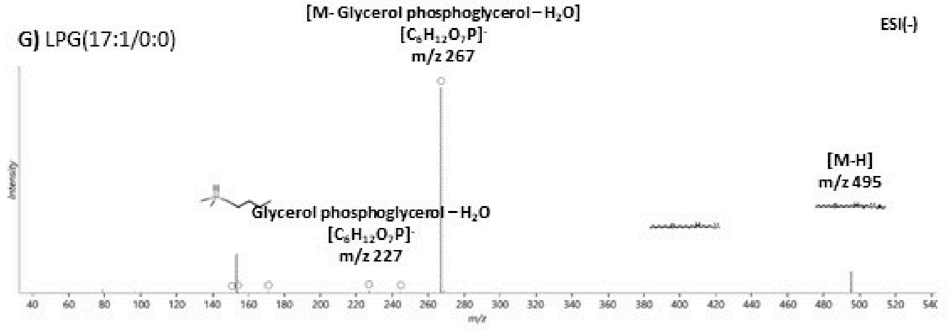
G) LPG(17:1/0:0)
[M- Glycerol phosphoglycerol − H_2O]
$[C_6H_{12}O_7P]^-$
m/z 267
ESI(-)
[M-H]
m/z 495
Glycerol phosphoglycerol − H_2O
$[C_6H_{12}O_7P]^-$
m/z 227
Intensity
m/z

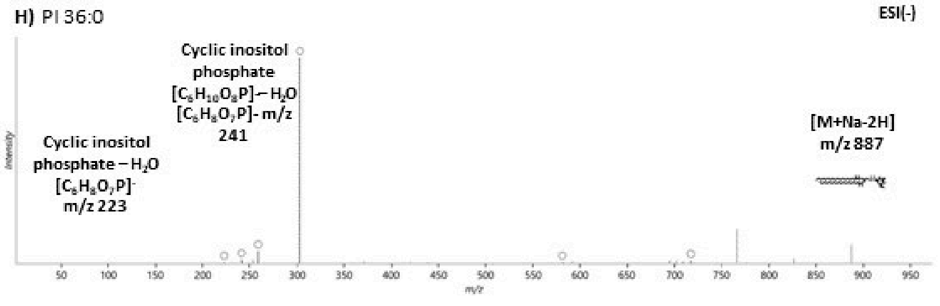
H) PI 36:0
ESI(-)
Cyclic inositol
phosphate
$[C_6H_{10}O_8P]^-$ − H_2O
$[C_6H_8O_7P]^-$ m/z
241
Cyclic inositol
phosphate − H_2O
$[C_6H_8O_7P]^-$
m/z 223
[M+Na-2H]
m/z 887
Intensity
m/z

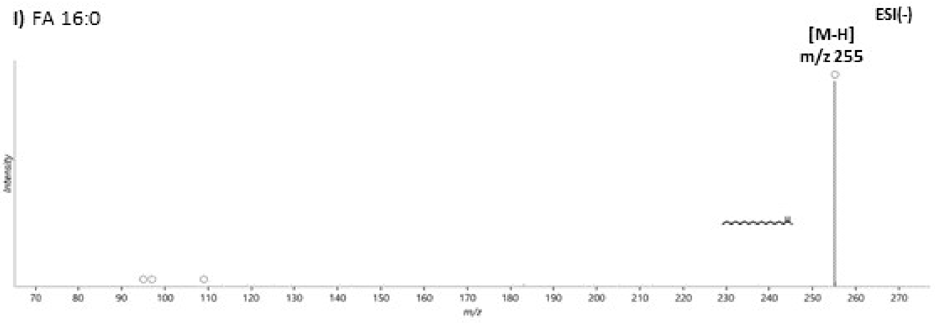
I) FA 16:0
ESI(-)
[M-H]
m/z 255
Intensity
m/z

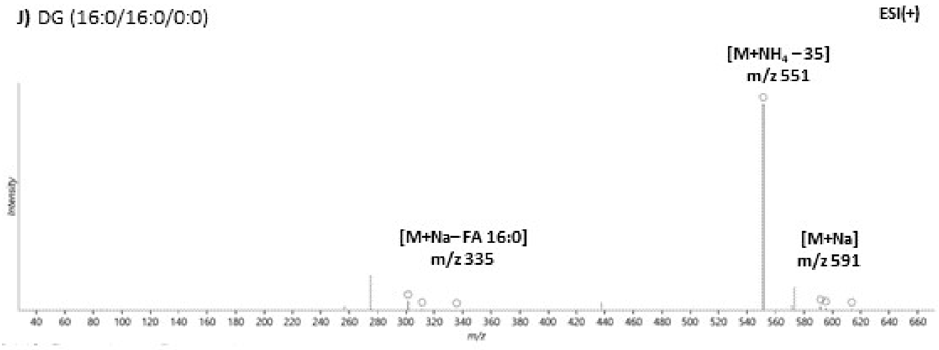
J) DG (16:0/16:0/0:0)
ESI(+)
[M+NH4 −35]
m/z 551
[M+Na–FA 16:0]
m/z 335
[M+Na]
m/z 591
Intensity
m/z

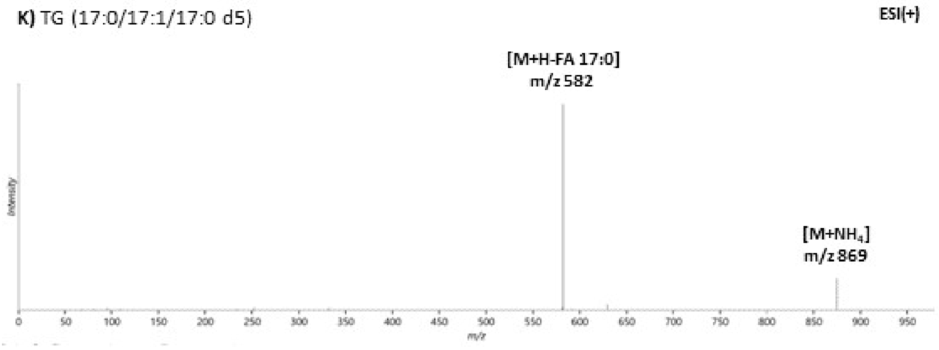
K) TG (17:0/17:1/17:0 d5)
ESI(+)
[M+H-FA 17:0]
m/z 582
[M+NH4]
m/z 869
Intensity
m/z

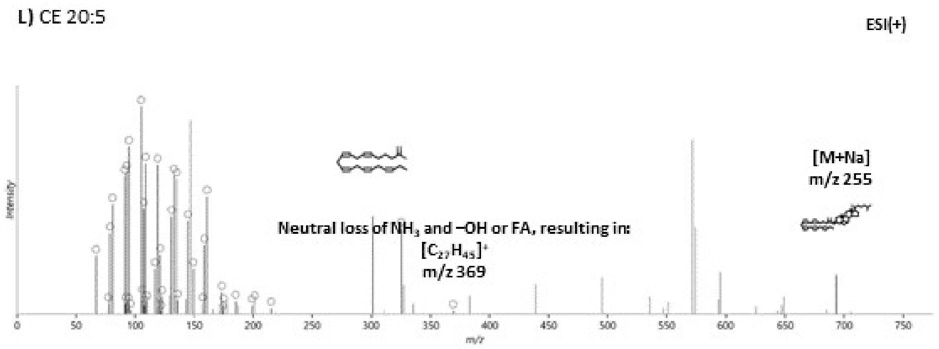
L) CE 20:5
ESI(+)
[M+Na]
m/z 255
Neutral loss of NH3 and −OH or FA, resulting in:
[C27H45]+
m/z 369
Intensity
m/z

M) SM 42:2 **ESI(+)**

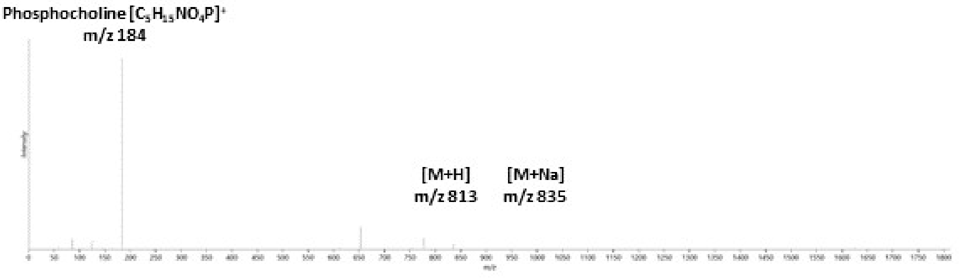

N) SM 42:2 **ESI(-)**

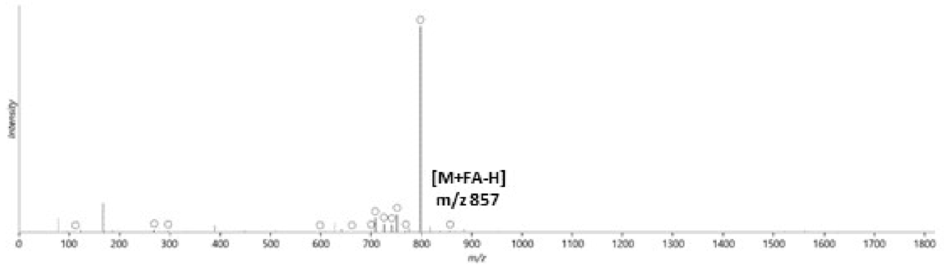

O) HexCer 34:1 **ESI(+)**

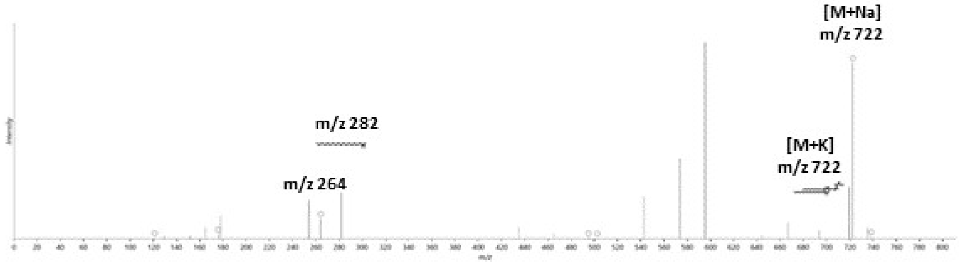

P) HexCer 34:1 **ESI(-)**

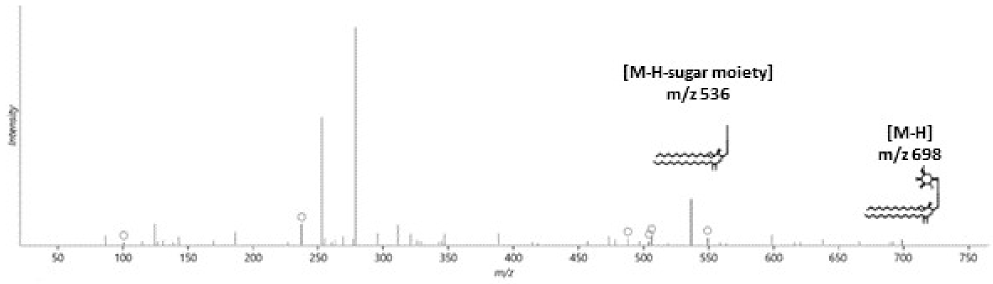

Figure S9. Representive ion ESI-MS spectra for a selection of lipid classes detected.

Panel A-B: Typical fragmentation pattern of LPC and PC. In ESI(+) LPCs and PCs were predominantly found in protonated form (Panel A), while in ESI(-) they were deprotonated or formed adducts with formic acid (Panel B). The ESI(+) spectrum was characterized by the presence of the fragment m/z 184 corresponding to the phosphatidyl choline head group.

Panel C-D: In ESI(+) LPEs and PEs were present in protonated form [M+H] (Panel C). Fragmentation was characterized by loss of the phosphoethanolamine head group [M+H-141]. In ESI(-) LPEs and PEs were deprotonated and the head group was detected at m/z 196 corresponding to the fragment [C5H11NO5P]- (glycerol phosphoethanolamine – H2O) (Panel D).

Panel E-F: LPS and PS were protonated in ESI(+). The fragmentation spectra were characterized by the loss of phopshorylserine (185.0089 amu [C3H8NO6P]+). In ESI(-) the lipids were deprotonated [M-H], and the fragmentation spectra were characterized by the neutral loss of serine and water (87.0320 amu).

Panel G: LPG and PG were predominantly detected in deprotonated form [M-H] in ESI(-). Fragmentation was characterized by m/z 227 representing [glycerol phosphoglycerol – H2O], and m/z 267 corresponding to [M- glycerol phosphoglycerol – H2O].

Panel H: PIs were predominantly detected in ESI(-) and formed the following adduct: [M+Na-2H]. Fragmentation was characterised by the presence of specific ions representing inositol headgroup fragments (m/z 223, 241 and 259).

Panel I: Free FA species were detected in deprotonated form [M-H]. No characteristic fragmentation pattern was observed.

Panel J: Ammonium and sodium adducts of DG were detected in ESI(+). Fragmentation was characterized by characteristic the neutral loss of the acyl group [M+Na-FA]. As well as a fragment ion at [M+NH4−35]+, corresponding to the sequential loss of ammonia and then water.

Panel K: TG(17:0/17:1/17:0 d5) Triglycerides are lipid molecules made up of a glycerol backbone and three fatty acids, connected via ester linkages. IN positive ionization mode they formed ammonium adducts [M+NH4 +]. Fragmentation was characterized by the neutral losses of one of the three fatty acid residues and the ammonium ion.

Panel L: CE 20:5 Fragmentation was characterized by the neutral loss of NH3 and –OH or FA, resulting in m/z 369 [C27H45]+ .

Panel M-N: Sphingomyelin species have a phopshocholine head group similar to LPCs and PCs In positive ionization mode generated [M+H] ions and fragmentation gave rise to m/z 184 ion.

Panel O-P: Glycosphingolipids including HexCer, Hex2Cer and Hex3Cer were detected in both ESI(+) and ESI(-). Sodium adducts of glycolipids were detected in ESI(+). In ESI(-) glycosphingolipids were deprotonated [M-H] and fragmentation pattern was characterised by loss of the hexose moeiety.

Table S8. Results from the Wilcoxon Rank Sum Test comparing control and PKU groups within the ESI(+) lipidomics dataset.

	Control							PKU											
**Feature**	**n**	**Median**		**Min**		**Max**		**n**	**Median**		**Min**		**Max**		**FC**	**U**	***p*-value**	***p*-value adjusted**	**Significant**
PC 34:1	21	1.67E+00	[	1.12E+00	-	2.22E+00	]	21	2.03E+00	[	1.72E+00	-	2.43E+00	]	1.23	49	3.55E-06	5.54E-04	*
PC 42:6	21	2.18E-03	[	9.30E-04	-	3.35E-03	]	21	3.80E-03	[	1.51E-03	-	7.14E-03	]	1.88	54	7.81E-06	5.54E-04	*
PI 40:3	21	2.83E-02	[	1.82E-02	-	5.07E-02	]	21	4.99E-02	[	3.20E-02	-	8.96E-02	]	1.64	54	7.81E-06	5.54E-04	*
PC 32:1	21	1.84E-01	[	9.58E-02	-	3.55E-01	]	21	2.88E-01	[	1.56E-01	-	6.03E-01	]	1.76	63	2.88E-05	1.53E-03	*
TG 45:5	21	3.26E-02	[	1.46E-02	-	5.87E-02	]	21	5.69E-02	[	2.46E-02	-	1.49E-01	]	1.85	67	4.94E-05	2.11E-03	*
PC O-44:4	21	5.75E-03	[	2.42E-03	-	1.20E-02	]	21	1.10E-02	[	2.90E-03	-	2.12E-02	]	1.69	77	1.73E-04	6.13E-03	*
1.28_375.2526n	21	8.16E-02	[	6.47E-02	-	1.66E-01	]	21	1.05E-01	[	6.63E-02	-	1.56E-01	]	1.26	80	2.45E-04	6.53E-03	*
PC O-34:2 or PC P-34:1	21	5.63E-02	[	2.43E-02	-	8.36E-02	]	21	3.62E-02	[	1.50E-02	-	5.86E-02	]	0.68	361	2.45E-04	6.53E-03	*
CerPE 38:2;O2 [iso 2]	21	7.84E-03	[	3.36E-03	-	1.39E-02	]	21	1.18E-02	[	6.71E-03	-	2.50E-02	]	1.57	82	3.08E-04	7.29E-03	*
11.67_360.1796n	21	2.65E-02	[	1.49E-02	-	4.34E-02	]	21	3.63E-02	[	2.21E-02	-	6.48E-02	]	1.35	93	9.95E-04	1.95E-02	*
CE 18:2	21	1.03E+00	[	8.77E-01	-	1.31E+00	]	21	8.69E-01	[	6.73E-01	-	1.31E+00	]	0.86	347	1.10E-03	1.95E-02	*
TG 56:7	21	1.55E-01	[	7.35E-02	-	5.69E-01	]	21	3.59E-01	[	1.45E-01	-	1.03E+00	]	1.97	94	1.10E-03	1.95E-02	*
TG 58:7	21	8.68E-03	[	2.50E-03	-	2.99E-02	]	21	1.74E-02	[	8.61E-03	-	4.77E-02	]	1.96	95	1.22E-03	1.99E-02	*
TG 50:2	21	1.30E+00	[	6.23E-01	-	3.77E+00	]	21	2.79E+00	[	1.47E+00	-	5.83E+00	]	1.69	96	1.34E-03	2.04E-02	*
PC 32:0	21	1.82E-01	[	1.27E-01	-	2.34E-01	]	21	2.21E-01	[	1.50E-01	-	2.89E-01	]	1.19	97	1.48E-03	2.10E-02	*
PC 38:3	21	5.41E-01	[	4.54E-01	-	1.07E+00	]	21	7.79E-01	[	5.06E-01	-	1.31E+00	]	1.33	98	1.63E-03	2.17E-02	*
3.23_384.1917n	21	4.00E-02	[	2.62E-02	-	4.97E-02	]	21	4.67E-02	[	3.32E-02	-	7.98E-02	]	1.22	99	1.79E-03	2.24E-02	*
PC 38:7 [iso 2]	21	7.44E-04	[	2.25E-04	-	1.17E-03	]	21	1.03E-03	[	5.60E-04	-	2.34E-03	]	1.54	102	2.37E-03	2.80E-02	*
PC 40:6	21	3.57E-01	[	1.79E-01	-	5.85E-01	]	21	4.49E-01	[	2.28E-01	-	7.63E-01	]	1.42	108	4.05E-03	4.32E-02	*
TG 50:1	21	6.95E-01	[	2.75E-01	-	1.79E+00	]	21	1.17E+00	[	5.91E-01	-	3.71E+00	]	1.81	108	4.05E-03	4.32E-02	*
13.22_646.4526n	21	2.25E+00	[	1.57E+00	-	3.08E+00	]	21	2.75E+00	[	1.83E+00	-	4.79E+00	]	1.23	110	4.81E-03	4.88E-02	*
16.27_644.5530n	21	3.02E-03	[	1.14E-03	-	8.23E-03	]	21	6.66E-03	[	1.13E-03	-	1.69E-02	]	1.76	115	7.29E-03	5.61E-02	
LPC 20:3	21	3.85E-01	[	1.22E-01	-	8.98E-01	]	21	7.19E-01	[	1.28E-01	-	2.48E+00	]	1.81	116	7.90E-03	5.61E-02	
LPC 22:6 [iso 2]	21	3.43E-01	[	1.21E-01	-	6.21E-01	]	21	5.41E-01	[	9.27E-02	-	1.64E+00	]	1.97	113	6.19E-03	5.61E-02	
PC 36:1	21	5.60E-01	[	3.27E-01	-	9.76E-01	]	21	6.64E-01	[	4.89E-01	-	1.09E+00	]	1.22	114	6.72E-03	5.61E-02	
PC 38:7 [iso 1]	21	4.35E-03	[	1.62E-03	-	6.77E-03	]	21	5.96E-03	[	2.55E-03	-	1.50E-02	]	1.65	114	6.72E-03	5.61E-02	
TG 48:0	21	4.69E-02	[	1.30E-02	-	1.69E-01	]	21	7.63E-02	[	2.74E-02	-	4.46E-01	]	2.55	113	6.19E-03	5.61E-02	
TG 48:1	21	1.65E-01	[	2.98E-02	-	5.37E-01	]	21	3.22E-01	[	1.06E-01	-	1.00E+00	]	1.92	116	7.90E-03	5.61E-02	
TG 50:0	21	2.98E-02	[	8.96E-03	-	8.51E-02	]	21	4.64E-02	[	1.75E-02	-	3.47E-01	]	2.48	116	7.90E-03	5.61E-02	

PC 36:6	21	2.56E-03	[1.33E-03 - 3.90E-03]	21	3.79E-03	[1.57E-03 - 8.80E-03]	1.61	117	8.55E-03	5.69E-02
TG 52:2	21	3.02E+00	[1.87E+00 - 9.70E+00]	21	5.10E+00	[2.22E+00 - 1.19E+01]	1.41	117	8.55E-03	5.69E-02
12.81_645.6061n	21	9.28E-03	[2.81E-03 - 1.63E-02]	21	1.09E-02	[7.26E-03 - 2.23E-02]	1.36	119	1.00E-02	5.76E-02
12.98_592.5911n	21	3.85E-02	[1.97E-02 - 7.31E-02]	21	5.07E-02	[2.85E-02 - 1.06E-01]	1.44	119	1.00E-02	5.76E-02
17.20_336.2925n	21	4.24E-01	[2.84E-01 - 7.23E-01]	21	3.16E-01	[1.82E-01 - 5.68E-01]	0.77	322	1.00E-02	5.76E-02
6.62_360.2341n	21	2.28E-01	[1.54E-02 - 5.70E-01]	21	4.42E-01	[1.43E-02 - 1.05E+00]	1.91	118	9.26E-03	5.76E-02
PC 40:5	21	1.29E-01	[8.10E-02 - 2.15E-01]	21	1.49E-01	[8.70E-02 - 2.72E-01]	1.23	119	1.00E-02	5.76E-02
8.42_974.7121n	21	3.04E-03	[2.30E-03 - 4.28E-03]	21	3.40E-03	[2.67E-03 - 6.58E-03]	1.20	120	1.08E-02	6.06E-02
LPC 22:6 [iso 1]	21	4.36E-02	[1.67E-02 - 8.69E-02]	21	7.37E-02	[1.19E-02 - 2.32E-01]	2.02	122	1.26E-02	6.70E-02
TG 52:1	21	2.88E-01	[8.75E-02 - 8.30E-01]	21	3.41E-01	[1.72E-01 - 2.39E+00]	2.08	122	1.26E-02	6.70E-02
PC O-36:4 or PC P-36:3	21	2.02E-01	[1.12E-01 - 3.83E-01]	21	1.50E-01	[1.06E-01 - 2.44E-01]	0.78	315	1.69E-02	8.79E-02
11.67_569.3674m/z	21	9.09E-02	[5.40E-02 - 1.32E-01]	21	1.09E-01	[6.17E-02 - 1.83E-01]	1.26	127	1.82E-02	8.80E-02
TG 51:1	21	1.96E-02	[5.71E-03 - 6.32E-02]	21	3.07E-02	[1.07E-02 - 1.22E-01]	1.64	127	1.82E-02	8.80E-02
TG 52:3	21	3.14E-01	[1.84E-01 - 7.97E-01]	21	4.93E-01	[1.53E-01 - 1.15E+00]	1.36	127	1.82E-02	8.80E-02
TG 58:9	21	9.17E-02	[2.30E-02 - 2.58E-01]	21	1.53E-01	[5.34E-02 - 3.83E-01]	1.63	128	1.95E-02	9.24E-02
DG 34:2 [iso 3]	21	3.77E-02	[1.52E-02 - 7.59E-02]	21	5.71E-02	[1.94E-02 - 1.49E-01]	1.54	129	2.09E-02	9.69E-02
13.90_598.4998n	21	7.27E-02	[3.13E-02 - 1.07E-01]	21	8.59E-02	[5.46E-02 - 1.35E-01]	1.23	130	2.24E-02	9.76E-02
LPG 20:5	21	1.05E-01	[6.35E-02 - 1.58E-01]	21	1.22E-01	[7.75E-02 - 1.63E-01]	1.18	130	2.24E-02	9.76E-02
TG 46:0	21	1.87E-02	[7.34E-03 - 8.40E-02]	21	3.70E-02	[1.25E-02 - 1.72E-01]	1.83	130	2.24E-02	9.76E-02
LPC 20:5 [iso 1]	21	1.74E-02	[6.97E-03 - 3.45E-02]	21	3.22E-02	[5.94E-03 - 1.01E-01]	1.89	131	2.40E-02	9.85E-02
LPC 20:5 [iso 2]	21	1.36E-01	[6.58E-02 - 3.04E-01]	21	2.77E-01	[5.26E-02 - 8.43E-01]	1.86	131	2.40E-02	9.85E-02
SM 34:0;O2	21	1.74E-01	[1.14E-01 - 2.16E-01]	21	1.30E-01	[8.29E-02 - 2.54E-01]	0.85	310	2.40E-02	9.85E-02
9.86_369.3522m/z	21	1.49E-01	[1.15E-01 - 1.92E-01]	21	1.64E-01	[1.32E-01 - 2.50E-01]	1.12	132	2.57E-02	1.03E-01
TG 58:10	21	4.12E-02	[8.76E-03 - 1.18E-01]	21	8.98E-02	[1.71E-02 - 2.29E-01]	1.78	134	2.94E-02	1.16E-01
PC 30:0	21	3.00E-02	[1.59E-02 - 7.47E-02]	21	3.54E-02	[2.05E-02 - 7.56E-02]	1.24	135	3.15E-02	1.22E-01
10.76_908.6610n	21	1.02E-02	[1.56E-03 - 1.83E-02]	21	1.14E-02	[6.40E-03 - 2.97E-02]	1.53	138	3.82E-02	1.45E-01
PC P-40:6	21	2.30E-02	[1.33E-02 - 3.78E-02]	21	2.88E-02	[1.44E-02 - 6.33E-02]	1.32	139	4.07E-02	1.49E-01
TG 51:2	21	1.26E-01	[5.51E-02 - 4.64E-01]	21	1.94E-01	[1.09E-01 - 4.16E-01]	1.32	139	4.07E-02	1.49E-01
LPC 22:5	21	1.00E-01	[3.93E-02 - 1.83E-01]	21	1.71E-01	[2.35E-02 - 7.01E-01]	1.91	140	4.33E-02	1.54E-01
SM 42:3;O2	21	1.47E+00	[8.70E-01 - 1.93E+00]	21	1.57E+00	[9.53E-01 - 2.42E+00]	1.16	140	4.33E-02	1.54E-01
DG 40:6	21	1.78E-03	[6.84E-04 - 4.52E-03]	21	2.46E-03	[8.95E-04 - 7.50E-03]	1.50	141	4.61E-02	1.58E-01
PC O-38:4 or PC P-38:3	21	6.77E-02	[3.39E-02 - 9.91E-02]	21	5.15E-02	[2.97E-02 - 9.66E-02]	0.82	300	4.61E-02	1.58E-01

PC 32:2	21	6.33E-02	[3.24E-02 - 2.09E-01]	21	8.40E-02	[3.92E-02 - 1.50E-01]	1.12	143	5.21E-02	1.76E-01
PE O-38:5 or PE P-38:4	21	6.03E-01	[2.22E-01 - 1.30E+00]	21	4.39E-01	[1.72E-01 - 1.09E+00]	0.74	297	5.53E-02	1.84E-01
PC 42:7	21	2.27E-03	[1.01E-03 - 4.06E-03]	21	2.93E-03	[1.07E-03 - 6.54E-03]	1.31	146	6.22E-02	2.04E-01
DG 36:2	21	8.87E-02	[3.85E-02 - 2.80E-01]	21	1.37E-01	[6.48E-02 - 5.09E-01]	1.34	147	6.59E-02	2.13E-01
3.77_355.2727n	21	8.86E-02	[3.84E-03 - 2.89E-01]	21	1.59E-01	[6.41E-03 - 6.30E-01]	1.89	148	6.98E-02	2.22E-01
PC O-32:0	21	2.57E-02	[1.67E-02 - 4.12E-02]	21	2.94E-02	[1.50E-02 - 4.62E-02]	1.14	149	7.39E-02	2.28E-01
SM 35:1;O2 [iso 1]	21	2.49E-02	[2.24E-03 - 4.96E-02]	21	1.91E-02	[5.35E-03 - 3.83E-02]	0.78	292	7.39E-02	2.28E-01
10.25_486.3745n	21	6.60E-02	[2.65E-02 - 9.64E-02]	21	7.83E-02	[4.09E-02 - 1.55E-01]	1.20	150	7.81E-02	2.34E-01
TG 53:3	21	1.16E-01	[5.36E-02 - 5.06E-01]	21	1.73E-01	[7.99E-02 - 3.89E-01]	1.23	150	7.81E-02	2.34E-01
9.15_678.4415n	21	1.11E-02	[8.36E-03 - 1.42E-02]	21	1.19E-02	[9.66E-03 - 1.89E-02]	1.10	151	8.26E-02	2.35E-01
CerP 32:1;O2	21	2.72E-03	[1.26E-03 - 4.41E-03]	21	3.60E-03	[8.28E-04 - 1.12E-02]	1.50	151	8.26E-02	2.35E-01
Hex2Cer 40:1;O2	21	4.02E-03	[2.22E-03 - 6.64E-03]	21	2.98E-03	[1.55E-03 - 7.55E-03]	0.82	290	8.26E-02	2.35E-01
TG 54:2	21	4.67E-01	[1.93E-01 - 1.66E+00]	21	5.86E-01	[3.73E-01 - 2.85E+00]	1.41	151	8.26E-02	2.35E-01
11.66_528.4212n	21	7.74E-02	[3.65E-02 - 1.01E-01]	21	8.66E-02	[5.06E-02 - 1.53E-01]	1.16	152	8.72E-02	2.38E-01
DG 36:3	21	5.23E-02	[2.63E-02 - 1.06E-01]	21	6.77E-02	[1.62E-02 - 2.11E-01]	1.40	152	8.72E-02	2.38E-01
TG 54:4	21	2.36E+00	[1.25E+00 - 6.40E+00]	21	2.98E+00	[1.01E+00 - 1.17E+01]	1.40	152	8.72E-02	2.38E-01
PC 42:10	21	3.80E-03	[2.08E-03 - 6.36E-03]	21	4.68E-03	[2.16E-03 - 8.16E-03]	1.21	153	9.21E-02	2.45E-01
LPC 20:5 [iso 3]	21	1.53E-02	[8.70E-03 - 2.08E-02]	21	1.40E-02	[7.82E-03 - 2.27E-02]	0.88	287	9.72E-02	2.52E-01
PC O-32:1 or PC P-32:0 [iso 2]	21	1.03E-02	[6.21E-03 - 1.69E-02]	21	1.14E-02	[6.45E-03 - 1.69E-02]	1.10	154	9.72E-02	2.52E-01
8.44_842.6329n	21	4.09E-03	[3.15E-03 - 5.84E-03]	21	4.49E-03	[3.23E-03 - 8.15E-03]	1.12	155	1.02E-01	2.57E-01
LPE 17:0	21	3.96E-02	[1.51E-03 - 9.01E-02]	21	6.81E-02	[6.00E-03 - 1.89E-01]	1.77	155	1.02E-01	2.57E-01
TG 46:2	21	5.85E-02	[8.56E-03 - 2.18E-01]	21	9.55E-02	[1.62E-02 - 2.25E-01]	1.36	155	1.02E-01	2.57E-01
Hex2Cer 34:1;O2	21	8.57E-02	[5.98E-02 - 1.38E-01]	21	1.04E-01	[5.64E-02 - 1.54E-01]	1.12	156	1.08E-01	2.67E-01
8.16_320.3086n	21	4.34E-02	[1.92E-02 - 1.44E-01]	21	5.37E-02	[3.12E-02 - 1.54E-01]	1.26	157	1.14E-01	2.72E-01
LPC 18:0 [iso 1]	21	1.39E-01	[7.97E-02 - 1.83E-01]	21	1.15E-01	[6.77E-02 - 2.45E-01]	0.93	284	1.14E-01	2.72E-01
PC 36:5	21	4.09E-01	[2.51E-01 - 5.27E-01]	21	4.59E-01	[2.27E-01 - 8.59E-01]	1.18	157	1.14E-01	2.72E-01
14.64_698.5886n	21	8.73E-02	[2.41E-02 - 2.15E-01]	21	4.86E-02	[1.88E-02 - 3.94E-01]	0.96	283	1.20E-01	2.77E-01
5.91_624.4483n	21	3.80E-03	[1.62E-03 - 1.07E-02]	21	5.69E-03	[6.56E-04 - 3.02E-02]	1.65	158	1.20E-01	2.77E-01
TG 54:6	21	6.07E-01	[2.22E-01 - 1.70E+00]	21	1.02E+00	[2.22E-01 - 5.77E+00]	1.84	158	1.20E-01	2.77E-01
PC O-32:1 or PC P-32:0 [iso 1]	21	3.90E-03	[2.49E-03 - 6.08E-03]	21	4.74E-03	[3.02E-03 - 6.77E-03]	1.13	159	1.26E-01	2.88E-01
SM 39:1;O2	21	3.79E-01	[1.73E-01 - 6.08E-01]	21	3.04E-01	[1.83E-01 - 5.64E-01]	0.88	281	1.32E-01	3.00E-01
PC O-34:1 or PC P-34:0	21	6.97E-02	[4.80E-02 - 1.17E-01]	21	8.05E-02	[4.75E-02 - 1.11E-01]	1.11	161	1.39E-01	3.11E-01

Amide C22	21	3.39E-02	[1.62E-02 - 9.06E-02]	21	4.07E-02	[2.47E-02 - 1.24E-01]	1.33	162	1.46E-01	3.17E-01
PE 36:1	21	7.62E-01	[2.36E-01 - 1.48E+00]	21	8.72E-01	[5.63E-01 - 1.95E+00]	1.23	162	1.46E-01	3.17E-01
PG 27:0 [iso 1]	21	2.08E-01	[1.20E-01 - 2.82E-01]	21	2.15E-01	[1.30E-01 - 3.54E-01]	1.14	162	1.46E-01	3.17E-01
Hex2Cer 42:2;O2	21	2.06E-02	[1.26E-02 - 3.49E-02]	21	2.28E-02	[1.07E-02 - 4.24E-02]	1.17	164	1.60E-01	3.45E-01
LPE 22:0	21	3.78E-02	[1.89E-02 - 6.45E-02]	21	2.65E-02	[1.17E-02 - 6.83E-02]	0.86	276	1.68E-01	3.58E-01
TG 54:3	21	2.65E+00	[1.05E+00 - 8.26E+00]	21	2.87E+00	[1.16E+00 - 8.75E+00]	1.28	166	1.76E-01	3.72E-01
PC 36:4	21	3.00E-01	[1.43E-01 - 5.20E-01]	21	2.62E-01	[1.24E-01 - 5.37E-01]	0.86	274	1.84E-01	3.78E-01
PC O-20:0	21	3.35E-03	[1.89E-03 - 7.10E-03]	21	2.92E-03	[1.74E-03 - 5.48E-03]	0.86	274	1.84E-01	3.78E-01
SM 33:1;O2	21	3.23E-01	[1.01E-01 - 5.75E-01]	21	2.45E-01	[1.46E-01 - 4.87E-01]	0.88	274	1.84E-01	3.78E-01
LPC 18:0 [iso 2]	21	1.01E+00	[7.16E-01 - 1.19E+00]	21	8.84E-01	[5.53E-01 - 1.53E+00]	0.94	273	1.93E-01	3.84E-01
PC O-36:5 or PC P-36:4	21	4.97E-03	[4.27E-03 - 6.44E-03]	21	5.43E-03	[4.10E-03 - 6.91E-03]	1.08	168	1.93E-01	3.84E-01
TG 48:3	21	1.52E-01	[3.64E-02 - 4.92E-01]	21	2.52E-01	[7.02E-02 - 5.55E-01]	1.27	168	1.93E-01	3.84E-01
PI 38:4	21	3.10E-02	[2.22E-02 - 4.70E-02]	21	3.61E-02	[2.08E-02 - 5.09E-02]	1.10	170	2.11E-01	4.16E-01
PI 38:5	21	7.39E-03	[2.62E-03 - 1.74E-02]	21	8.79E-03	[4.67E-03 - 1.59E-02]	1.18	171	2.20E-01	4.26E-01
SM 41:1;O2	21	9.18E-01	[5.26E-01 - 1.36E+00]	21	8.25E-01	[4.65E-01 - 1.37E+00]	0.91	270	2.20E-01	4.26E-01
11.76_609.5007n	21	2.51E-02	[1.22E-02 - 4.33E-02]	21	2.63E-02	[1.08E-02 - 6.22E-02]	1.18	174	2.50E-01	4.67E-01
LPE 18:0	21	1.21E-01	[5.68E-02 - 1.82E-01]	21	9.83E-02	[3.55E-02 - 2.05E-01]	0.88	267	2.50E-01	4.67E-01
PC 42:1	21	9.09E-04	[5.89E-04 - 2.82E-03]	21	1.25E-03	[5.37E-04 - 2.50E-03]	1.12	174	2.50E-01	4.67E-01
TG 53:2	21	1.27E-02	[5.63E-03 - 3.33E-02]	21	1.58E-02	[8.42E-03 - 4.05E-02]	1.24	174	2.50E-01	4.67E-01
8.64_440.4100m/z	21	1.09E-02	[9.50E-03 - 1.31E-02]	21	1.17E-02	[8.30E-03 - 1.65E-02]	1.05	176	2.71E-01	4.78E-01
CerPE 38:2;O2 [iso 1]	21	2.32E-02	[8.21E-03 - 4.55E-02]	21	1.99E-02	[9.65E-03 - 3.57E-02]	0.90	265	2.71E-01	4.78E-01
DG 32:5	21	5.92E-03	[3.17E-03 - 2.46E-02]	21	5.10E-03	[2.47E-03 - 1.38E-02]	0.79	266	2.61E-01	4.78E-01
PI 34:2	21	1.57E-02	[9.61E-03 - 2.39E-02]	21	1.82E-02	[7.53E-03 - 2.71E-02]	1.11	176	2.71E-01	4.78E-01
SM 35:1;O2 [iso 2]	21	5.88E-02	[2.87E-02 - 1.17E-01]	21	5.42E-02	[3.50E-02 - 7.99E-02]	0.90	265	2.71E-01	4.78E-01
TG 46:1	21	7.99E-02	[1.33E-02 - 2.95E-01]	21	1.03E-01	[2.47E-02 - 3.74E-01]	1.24	175	2.61E-01	4.78E-01
TG 54:5	21	2.69E-01	[8.03E-02 - 7.10E-01]	21	2.94E-01	[1.65E-01 - 1.14E+00]	1.19	176	2.71E-01	4.78E-01
PE 40:4	21	9.56E-01	[4.90E-01 - 1.37E+00]	21	7.55E-01	[3.48E-01 - 1.59E+00]	0.89	264	2.83E-01	4.93E-01
1.09_460.1374n	21	5.40E-02	[5.59E-03 - 8.77E-02]	21	3.59E-02	[9.73E-03 - 8.45E-02]	0.85	262	3.06E-01	5.17E-01
5.81_593.3691n	21	1.32E-02	[6.21E-03 - 5.24E-02]	21	1.02E-02	[5.37E-03 - 3.77E-02]	0.86	262	3.06E-01	5.17E-01
DG 40:5	21	1.71E-03	[8.95E-04 - 3.10E-03]	21	1.91E-03	[8.61E-04 - 4.81E-03]	1.15	179	3.06E-01	5.17E-01
TG 44:0	21	1.26E-02	[4.75E-03 - 4.16E-02]	21	1.60E-02	[6.16E-03 - 4.94E-02]	1.26	179	3.06E-01	5.17E-01
9.44_859.6018n	21	4.37E-03	[1.96E-03 - 7.62E-03]	21	4.80E-03	[1.66E-03 - 8.00E-03]	1.14	180	3.18E-01	5.25E-01

DG 34:2 [iso 1]	21	1.92E-02	[1.05E-02 - 2.95E-02]	21	2.30E-02	[8.87E-03 - 3.81E-02]	1.12	180	3.18E-01	5.25E-01
LPC O-18:0	21	3.15E-02	[1.87E-02 - 5.00E-02]	21	2.75E-02	[7.72E-03 - 6.21E-02]	0.90	261	3.18E-01	5.25E-01
TG 55:4	21	6.99E-03	[2.00E-03 - 2.31E-02]	21	6.70E-03	[4.40E-03 - 2.42E-02]	1.14	181	3.30E-01	5.41E-01
PE 34:1	21	6.07E-02	[4.18E-02 - 9.56E-02]	21	6.83E-02	[3.22E-02 - 1.33E-01]	1.10	182	3.43E-01	5.53E-01
TG 56:3	21	9.04E-02	[3.10E-02 - 2.85E-01]	21	9.40E-02	[4.49E-02 - 3.24E-01]	1.19	182	3.43E-01	5.53E-01
MG 20:0	21	1.07E-02	[8.05E-03 - 1.75E-02]	21	1.05E-02	[6.72E-03 - 1.74E-02]	0.94	258	3.56E-01	5.65E-01
SM 32:0;O2	21	3.62E-02	[2.01E-02 - 6.34E-02]	21	3.89E-02	[2.06E-02 - 6.57E-02]	0.91	258	3.56E-01	5.65E-01
CerPE 36:2;O2	21	1.19E-02	[2.95E-03 - 2.38E-02]	21	8.84E-03	[3.29E-03 - 1.92E-02]	0.89	257	3.69E-01	5.69E-01
LPC 20:4	21	1.00E+00	[7.03E-01 - 1.94E+00]	21	1.32E+00	[3.94E-01 - 2.34E+00]	1.18	184	3.69E-01	5.69E-01
PC 34:4	21	2.85E-02	[1.15E-02 - 6.10E-02]	21	3.03E-02	[1.72E-02 - 6.63E-02]	1.16	184	3.69E-01	5.69E-01
8.16_460.3226n	21	7.43E-03	[3.00E-04 - 3.14E-02]	21	6.12E-03	[9.19E-04 - 1.67E-02]	0.78	256	3.82E-01	5.78E-01
CE 18:1	21	4.28E-01	[3.39E-01 - 5.59E-01]	21	4.12E-01	[3.13E-01 - 5.43E-01]	0.96	256	3.82E-01	5.78E-01
LPC O-18:1 or LPC P-18:0	21	1.65E-02	[7.76E-03 - 2.33E-02]	21	1.54E-02	[5.76E-03 - 3.13E-02]	0.94	256	3.82E-01	5.78E-01
3.64_800.1948n	21	8.90E-03	[6.02E-04 - 5.62E-02]	21	1.22E-02	[2.74E-03 - 5.46E-02]	1.01	186	3.96E-01	5.82E-01
DG 36:4 [iso 2]	21	1.19E-02	[5.48E-03 - 2.64E-02]	21	1.54E-02	[2.84E-03 - 5.08E-02]	1.32	186	3.96E-01	5.82E-01
TG 42:0	21	8.90E-03	[4.27E-03 - 2.24E-02]	21	9.39E-03	[4.09E-03 - 3.58E-02]	1.23	186	3.96E-01	5.82E-01
TG 54:1	21	2.57E-02	[4.01E-03 - 1.11E-01]	21	2.99E-02	[8.21E-03 - 5.81E-01]	2.04	186	3.96E-01	5.82E-01
CE 22:5	21	5.13E-03	[1.08E-03 - 1.01E-02]	21	4.35E-03	[9.99E-04 - 1.28E-02]	0.91	254	4.10E-01	5.95E-01
SM 32:1;O2	21	5.47E-01	[3.30E-01 - 9.32E-01]	21	5.10E-01	[3.44E-01 - 9.14E-01]	0.94	254	4.10E-01	5.95E-01
SM 42:1;O2	21	1.71E+00	[1.16E+00 - 2.40E+00]	21	1.58E+00	[1.06E+00 - 2.79E+00]	0.96	253	4.25E-01	6.07E-01
TG 44:1	21	1.83E-02	[2.66E-03 - 8.08E-02]	21	2.01E-02	[2.53E-03 - 1.07E-01]	1.23	188	4.25E-01	6.07E-01
PE 38:5	21	8.05E-02	[2.60E-02 - 1.34E-01]	21	8.99E-02	[2.23E-02 - 2.27E-01]	1.21	191	4.70E-01	6.67E-01
10.84_839.5204n	21	1.76E-02	[7.81E-03 - 2.95E-02]	21	1.99E-02	[6.63E-03 - 2.54E-02]	1.00	193	5.01E-01	7.02E-01
PC 40:1	21	1.41E-03	[9.65E-04 - 2.84E-03]	21	1.79E-03	[9.33E-04 - 3.17E-03]	1.07	193	5.01E-01	7.02E-01
Hex3Cer 34:1;O2	21	9.67E-03	[7.03E-03 - 1.43E-02]	21	9.64E-03	[5.82E-03 - 1.77E-02]	1.05	194	5.17E-01	7.20E-01
5.60_498.3417n	21	1.37E-02	[1.00E-02 - 1.67E-02]	21	1.27E-02	[1.03E-02 - 2.37E-02]	1.01	246	5.33E-01	7.33E-01
SM 34:1;O2	21	3.63E+00	[3.00E+00 - 4.87E+00]	21	3.46E+00	[2.77E+00 - 5.66E+00]	0.99	246	5.33E-01	7.33E-01
1.27_386.1735n	21	3.19E-01	[2.14E-01 - 4.43E-01]	21	3.25E-01	[1.71E-01 - 5.01E-01]	1.06	196	5.50E-01	7.37E-01
1.83_446.2537m/z	21	7.00E-03	[4.56E-03 - 1.77E-02]	21	6.62E-03	[3.67E-03 - 2.07E-02]	0.96	245	5.50E-01	7.37E-01
16.16_348.3091n	21	1.19E-02	[3.47E-03 - 2.87E-02]	21	8.13E-03	[3.30E-03 - 3.61E-02]	0.92	245	5.50E-01	7.37E-01
16.16_682.5942n	21	2.00E+00	[5.51E-01 - 4.92E+00]	21	1.25E+00	[7.97E-01 - 7.22E+00]	1.01	245	5.50E-01	7.37E-01
16.16_444.3276n	21	2.57E-02	[5.81E-03 - 4.64E-02]	21	1.60E-02	[9.30E-03 - 8.65E-02]	1.07	244	5.67E-01	7.45E-01

TG 42:4	21	6.62E-03	[	3.82E-03	-	3.11E-02	]	21	6.18E-03	[	3.10E-03	-	1.78E-02	]	0.79	244	5.67E-01	7.45E-01
TG 56:1	21	1.91E-03	[	6.85E-07	-	2.73E-02	]	21	2.09E-03	[	4.64E-07	-	6.87E-02	]	1.23	244	5.67E-01	7.45E-01
10.40_184.0745m/z	21	5.94E-02	[	4.63E-02	-	7.47E-02	]	21	6.12E-02	[	4.40E-02	-	8.18E-02	]	1.03	198	5.84E-01	7.53E-01
11.03_694.4958m/z	21	5.43E-03	[	2.15E-03	-	1.11E-02	]	21	5.14E-03	[	2.73E-03	-	1.46E-02	]	0.97	243	5.84E-01	7.53E-01
16.16_430.3120n	21	8.93E-02	[	2.42E-02	-	2.15E-01	]	21	5.59E-02	[	3.36E-02	-	3.80E-01	]	1.06	243	5.84E-01	7.53E-01
5.60_598.4326n	21	3.01E-02	[	1.98E-02	-	4.40E-02	]	21	2.70E-02	[	1.42E-02	-	7.06E-02	]	1.03	242	6.01E-01	7.62E-01
LPC 16:0 [iso 2]	21	6.87E+00	[	5.36E+00	-	7.97E+00	]	21	6.40E+00	[	4.86E+00	-	9.76E+00	]	1.00	242	6.01E-01	7.62E-01
TG 56:5	21	1.15E-01	[	5.66E-02	-	3.61E-01	]	21	1.16E-01	[	4.22E-02	-	2.20E-01	]	0.98	199	6.01E-01	7.62E-01
PC O-34:0	21	9.30E-03	[	5.87E-03	-	1.48E-02	]	21	8.37E-03	[	4.78E-03	-	1.74E-02	]	0.98	241	6.18E-01	7.79E-01
HexCer 34:1;O2	21	3.22E-02	[	2.28E-02	-	4.77E-02	]	21	2.95E-02	[	1.67E-02	-	4.85E-02	]	0.97	240	6.36E-01	7.92E-01
SM 34:2;O2	21	9.55E-01	[	6.73E-01	-	1.37E+00	]	21	1.01E+00	[	7.11E-01	-	1.58E+00	]	1.06	201	6.36E-01	7.92E-01
16.84_646.5692n	21	1.61E-01	[	1.17E-01	-	2.48E-01	]	21	1.62E-01	[	6.00E-02	-	3.56E-01	]	1.02	239	6.54E-01	8.05E-01
HexCer 40:1;O2	21	4.61E-02	[	3.00E-02	-	6.44E-02	]	21	5.19E-02	[	2.53E-02	-	7.17E-02	]	1.02	202	6.54E-01	8.05E-01
2.77_620.1517n	21	2.92E-02	[	1.20E-02	-	7.08E-02	]	21	2.57E-02	[	1.00E-02	-	7.07E-02	]	0.95	238	6.72E-01	8.09E-01
SM 36:3;O2	21	5.42E-02	[	2.78E-02	-	9.24E-02	]	21	5.24E-02	[	3.00E-02	-	8.09E-02	]	0.96	238	6.72E-01	8.09E-01
SM 40:1;O2	21	1.71E+00	[	1.25E+00	-	2.50E+00	]	21	1.69E+00	[	1.08E+00	-	2.66E+00	]	0.98	238	6.72E-01	8.09E-01
TG 56:2	21	2.38E-02	[	1.42E-03	-	1.03E-01	]	21	2.40E-02	[	5.04E-03	-	1.92E-01	]	1.23	203	6.72E-01	8.09E-01
5.15_622.4327n	21	1.20E-02	[	4.86E-03	-	2.95E-02	]	21	1.29E-02	[	1.99E-03	-	5.58E-02	]	1.29	204	6.90E-01	8.21E-01
DG 34:2 [iso 2]	21	3.96E-03	[	1.26E-03	-	5.98E-03	]	21	3.75E-03	[	9.77E-04	-	1.12E-02	]	1.14	204	6.90E-01	8.21E-01
Cer 42:1;O2	21	2.02E-01	[	1.38E-01	-	3.60E-01	]	21	2.13E-01	[	1.12E-01	-	4.01E-01	]	1.07	205	7.09E-01	8.25E-01
DG 36:4 [iso 1]	21	8.84E-03	[	5.68E-03	-	1.84E-02	]	21	1.02E-02	[	4.91E-03	-	1.86E-02	]	1.04	205	7.09E-01	8.25E-01
DG 38:4	21	8.24E-02	[	5.79E-02	-	1.33E-01	]	21	9.63E-02	[	4.74E-02	-	1.79E-01	]	1.06	205	7.09E-01	8.25E-01
LPC 16:0 [iso 1]	21	1.02E+00	[	6.62E-01	-	1.28E+00	]	21	8.78E-01	[	5.61E-01	-	1.84E+00	]	1.03	236	7.09E-01	8.25E-01
13.60_793.6793n	21	8.42E-03	[	4.84E-03	-	2.41E-02	]	21	7.98E-03	[	2.86E-03	-	1.82E-02	]	0.93	235	7.27E-01	8.33E-01
2.98_576.1260n	21	3.68E-01	[	1.92E-01	-	8.55E-01	]	21	3.51E-01	[	1.40E-01	-	8.31E-01	]	0.91	235	7.27E-01	8.33E-01
CE 16:0	21	7.36E-02	[	4.39E-02	-	1.26E-01	]	21	7.48E-02	[	4.10E-02	-	1.14E-01	]	0.94	235	7.27E-01	8.33E-01
4.79_768.1700n	21	9.15E-02	[	4.88E-02	-	1.94E-01	]	21	9.49E-02	[	3.75E-02	-	1.86E-01	]	1.01	208	7.65E-01	8.53E-01
LPC 20:1	21	7.84E-02	[	3.66E-02	-	1.66E-01	]	21	7.24E-02	[	3.04E-02	-	1.74E-01	]	0.97	233	7.65E-01	8.53E-01
PC O-36:0	21	1.48E-03	[	7.95E-04	-	2.95E-03	]	21	1.62E-03	[	6.83E-04	-	3.75E-03	]	1.07	208	7.65E-01	8.53E-01
PG 27:0 [iso 2]	21	7.24E-01	[	3.51E-01	-	1.12E+00	]	21	7.21E-01	[	3.30E-01	-	1.38E+00	]	1.07	208	7.65E-01	8.53E-01
PE 32:0	21	3.22E-02	[	1.79E-02	-	8.02E-02	]	21	2.72E-02	[	1.89E-02	-	6.62E-02	]	1.03	232	7.84E-01	8.66E-01
SM 36:2;O2	21	4.65E-01	[	2.31E-01	-	7.72E-01	]	21	4.59E-01	[	3.65E-01	-	7.17E-01	]	1.06	209	7.84E-01	8.66E-01

	2	1.71E-		6.06E-		3.85E-		2	1.77E-		6.38E-		3.87E-		0.9	21	8.03E-	
1.53_638.1636n	1	02	[	03	-	02	]	1	02	[	03	-	02	]	9	0	01	8.78E-01
	2	9.32E-		4.94E-		1.33E+		2	9.32E-		5.88E-		1.15E+		0.9	23	8.03E-	
SM 38:1;O2	1	01	[	01	-	00	]	1	01	[	01	-	00	]	7	1	01	8.78E-01
	2	5.02E-		2.31E-		9.90E-		2	4.76E-		2.69E-		7.47E-		0.9	21	8.23E-	
PC 33:2	1	02	[	02	-	02	]	1	02	[	02	-	02	]	9	1	01	8.94E-01
	2	4.69E-		3.11E-		7.33E-		2	4.88E-		2.87E-		7.09E-		1.0	21	8.42E-	
12.90_809.6510n	1	02	[	02	-	02	]	1	02	[	02	-	02	]	1	2	01	9.06E-01
	2	1.00E-		5.08E-		1.81E-		2	9.16E-		3.86E-		2.63E-		1.0	21	8.42E-	
16.91_696.5844n	1	01	[	02	-	01	]	1	02	[	02	-	01	]	9	2	01	9.06E-01
	2	1.94E-		8.45E-		3.56E-		2	1.84E-		1.05E-		3.40E-		1.0	21	8.81E-	
LPC 14:0 [iso 1]	1	02	[	03	-	02	]	1	02	[	02	-	02	]	2	4	01	9.39E-01
	2	7.05E-		3.42E-		1.05E+		2	7.50E-		3.34E-		1.29E+		1.0	21	8.81E-	
PG 25:0	1	01	[	01	-	00	]	1	01	[	01	-	00	]	5	4	01	9.39E-01
	2	1.73E-		7.41E-		3.21E-		2	1.58E-		8.42E-		2.84E-		1.0	21	9.01E-	
LPC 14:0 [iso 2]	1	01	[	02	-	01	]	1	01	[	02	-	01	]	2	5	01	9.55E-01
	2	1.65E-		6.25E-		4.59E-		2	1.66E-		8.53E-		5.10E-		1.0	21	9.21E-	
4.96_376.2293n	1	01	[	03	-	01	]	1	01	[	03	-	01	]	3	6	01	9.57E-01
	2	1.13E-		7.24E-		2.12E-		2	1.15E-		6.42E-		1.69E-		0.9	21	9.21E-	
Hex2Cer 32:1;O2	1	02	[	03	-	02	]	1	02	[	03	-	02	]	9	6	01	9.57E-01
	2	2.22E-		1.04E-		8.98E-		2	2.54E-		1.18E-		4.21E-		0.7	22	9.21E-	
PC 42:2	1	03	[	03	-	03	]	1	03	[	03	-	03	]	9	5	01	9.57E-01
	2	2.49E-		1.38E-		3.75E-		2	2.49E-		1.71E-		3.16E-		1.0	21	9.21E-	
SM 38:2;O2	1	01	[	01	-	01	]	1	01	[	01	-	01	]	1	6	01	9.57E-01
	2	1.70E-		8.43E-		4.23E-		2	1.67E-		5.72E-		4.04E-		1.0	22	9.40E-	
3.74_812.1948n	1	02	[	03	-	02	]	1	02	[	03	-	02	]	0	4	01	9.68E-01
	2	2.72E-		6.96E-		5.13E-		2	2.85E-		1.61E-		4.40E-		0.9	22	9.40E-	
PE 38:3	1	01	[	02	-	01	]	1	01	[	01	-	01	]	7	4	01	9.68E-01
	2	3.49E-		2.06E-		6.23E-		2	3.70E-		1.48E-		1.06E+		1.1	21	9.60E-	
4.86_519.3333n	1	01	[	01	-	01	]	1	01	[	01	-	00	]	5	8	01	9.79E-01
	2	5.92E-		3.06E-		1.49E-		2	5.79E-		3.26E-		2.44E-		1.1	21	9.60E-	
TG 40:0	1	03	[	03	-	02	]	1	03	[	03	-	02	]	0	8	01	9.79E-01
	2	1.14E-		1.90E-		5.12E-		2	1.31E-		4.09E-		5.16E-		0.9	21	9.80E-	
2.40_608.1526n	1	01	[	02	-	01	]	1	01	[	02	-	01	]	4	9	01	9.89E-01
	2	5.02E-		3.24E-		9.04E-		2	4.48E-		2.98E-		9.62E-		1.0	22	9.80E-	
SM 32:2;O2	1	02	[	02	-	02	]	1	02	[	02	-	02	]	4	2	01	9.89E-01
	2	3.16E-		4.57E-		7.15E-		2	3.09E-		5.56E-		7.65E-		1.0	22	1.00E+	
4.96_415.1927m/z	1	02	[	03	-	02	]	1	02	[	03	-	02	]	2	0	00	1.00E+00
	2	2.03E+		1.33E+		3.32E+		2	2.11E+		9.61E-		4.94E+		1.1	22	1.00E+	
5.15_519.3337n	1	00	[	00	-	00	]	1	00	[	01	-	00	]	0	0	00	1.00E+00

[1] The foldchange was calculated as $\bar{x}_{pku} / \bar{x}_{control}$.

Table S9. Results from the Wilcoxon Rank Sum Test comparing control and PKU groups within the ESI(-) lipidomics dataset.

	Control							PKU											
**Feature**	**n**	**Median**		**Min**		**Max**		**n**	**Median**		**Min**		**Max**		**FC I**	**U**	***p*-value**	***p*-value adjusted**	**Significant**
13.08_752.5923 m/z	21	0.056	[	2.24E-02	-	1.21E-01	]	21	0.081	[	4.67E-02	-	1.93E-01	]	1.59	75	1.36E-04	3.22E-03	*
LPC 17:0	21	1.019	[	7.39E-01	-	1.25E+00	]	21	0.759	[	5.98E-01	-	1.66E+00	]	0.81	370	8.28E-05	3.22E-03	*
PC 34:1	21	2.332	[	1.58E+00	-	4.48E+00	]	21	2.908	[	2.21E+00	-	4.17E+00	]	1.24	76	1.53E-04	3.22E-03	*
PC 38:3	21	0.566	[	3.85E-01	-	2.30E+00	]	21	0.797	[	5.17E-01	-	1.28E+00	]	1.20	87	5.33E-04	8.40E-03	*
11.62_833.6073 n	21	0.181	[	1.21E-01	-	2.63E-01	]	21	0.231	[	1.42E-01	-	3.66E-01	]	1.33	103	2.60E-03	2.93E-02	*
Hex2Cer 32:0;O2	21	0.061	[	4.21E-02	-	1.21E-01	]	21	0.072	[	4.57E-02	-	1.17E-01	]	1.18	107	3.72E-03	2.93E-02	*
PC 36:1	21	0.615	[	3.90E-01	-	1.31E+00	]	21	0.715	[	5.02E-01	-	1.22E+00	]	1.19	104	2.84E-03	2.93E-02	*
PC 36:5	21	0.235	[	1.21E-01	-	4.48E-01	]	21	0.347	[	1.05E-01	-	8.82E-01	]	1.50	107	3.72E-03	2.93E-02	*
10.76_805.5592 n	21	0.406	[	2.42E-01	-	6.19E-01	]	21	0.537	[	3.50E-01	-	1.02E+00	]	1.33	109	4.42E-03	3.09E-02	*
LPC 20:3	21	0.165	[	1.05E-01	-	2.79E-01	]	21	0.206	[	1.24E-01	-	3.75E-01	]	1.28	112	5.70E-03	3.26E-02	*
PI 36:4	21	0.053	[	2.65E-02	-	2.51E-01	]	21	0.080	[	3.85E-02	-	1.47E-01	]	1.16	112	5.70E-03	3.26E-02	*
PI 38:3	21	0.022	[	1.22E-02	-	6.15E-02	]	21	0.033	[	1.83E-02	-	8.03E-02	]	1.43	114	6.72E-03	3.53E-02	*
SM 42:3;O2	21	0.223	[	1.47E-01	-	2.94E-01	]	21	0.250	[	1.76E-01	-	3.41E-01	]	1.16	124	1.46E-02	7.08E-02	
DHA	21	0.024	[	1.26E-02	-	7.12E-02	]	21	0.037	[	1.64E-02	-	1.80E-01	]	1.73	126	1.69E-02	7.61E-02	
LPE 18:0	21	0.184	[	1.34E-01	-	2.56E-01	]	21	0.151	[	1.11E-01	-	2.17E-01	]	0.87	313	1.95E-02	7.68E-02	
PI 38:2	21	0.019	[	1.35E-02	-	5.13E-02	]	21	0.024	[	1.52E-02	-	4.13E-02	]	1.15	128	1.95E-02	7.68E-02	
FA 17:0	21	0.012	[	1.02E-02	-	1.80E-02	]	21	0.013	[	1.06E-02	-	2.26E-02	]	1.10	133	2.75E-02	9.64E-02	
LPC 16:0	21	3.765	[	3.01E+00	-	6.00E+00	]	21	3.315	[	2.55E+00	-	5.24E+00	]	0.88	308	2.75E-02	9.64E-02	
PC O-44:5	21	0.038	[	2.35E-02	-	4.95E-02	]	21	0.042	[	2.03E-02	-	6.45E-02	]	1.18	134	2.94E-02	9.76E-02	
LPE 22:6	21	0.013	[	8.88E-03	-	2.28E-02	]	21	0.018	[	9.80E-03	-	3.54E-02	]	1.32	141	4.61E-02	1.45E-01	
IPC 36:0;O2	21	0.008	[	5.68E-03	-	1.33E-02	]	21	0.010	[	6.19E-03	-	1.19E-02	]	1.13	148	6.98E-02	2.00E-01	
PI 40:3 [iso 1]	21	0.163	[	1.04E-01	-	2.75E-01	]	21	0.186	[	1.34E-01	-	2.74E-01	]	1.12	148	6.98E-02	2.00E-01	
10.52_848.5764 n	21	0.607	[	3.95E-01	-	1.25E+00	]	21	0.663	[	4.51E-01	-	1.05E+00	]	1.10	153	9.21E-02	2.30E-01	
Cer 34:1;O2	21	0.026	[	1.97E-02	-	4.13E-02	]	21	0.028	[	1.56E-02	-	4.76E-02	]	1.15	153	9.21E-02	2.30E-01	
FA 18:3	21	0.024	[	1.07E-02	-	8.31E-02	]	21	0.031	[	9.04E-03	-	7.52E-02	]	1.26	155	1.02E-01	2.30E-01	
PC 32:2	21	0.059	[	3.64E-02	-	1.82E-01	]	21	0.072	[	4.55E-02	-	1.20E-01	]	1.02	155	1.02E-01	2.30E-01	
PI 34:1	21	0.071	[	3.84E-02	-	2.23E-01	]	21	0.087	[	4.71E-02	-	2.35E-01	]	1.20	154	9.72E-02	2.30E-01	
PI 34:2	21	0.057	[	2.51E-02	-	3.50E-01	]	21	0.071	[	3.93E-02	-	1.48E-01	]	1.00	155	1.02E-01	2.30E-01	
FA 22:5	21	0.010	[	6.70E-03	-	2.74E-02	]	21	0.013	[	6.66E-03	-	2.37E-02	]	1.15	158	1.20E-01	2.60E-01	

LPG 17:0	21	0.017	[	5.97E-03	-	9.80E-02	]	21	0.023	[	6.79E-03	-	8.39E-02	]	1.50	159	1.26E-01	2.64E-01
PS O-38:6	21	0.054	[	2.28E-02	-	9.22E-02	]	21	0.063	[	3.11E-02	-	1.15E-01	]	1.17	160	1.32E-01	2.69E-01
PI 38:1	21	0.040	[	2.15E-02	-	6.18E-02	]	21	0.042	[	3.24E-02	-	6.50E-02	]	1.15	161	1.39E-01	2.74E-01
Cer 38:1;O2	21	0.020	[	1.21E-02	-	3.66E-02	]	21	0.023	[	1.35E-02	-	3.79E-02	]	1.14	167	1.84E-01	3.52E-01
12.09_564.5560n	21	0.135	[	7.56E-02	-	2.84E-01	]	21	0.154	[	9.61E-02	-	2.84E-01	]	1.05	172	2.30E-01	4.26E-01
11.18_582.5319n	21	0.108	[	6.68E-02	-	2.23E-01	]	21	0.120	[	7.98E-02	-	2.19E-01	]	1.05	174	2.50E-01	4.50E-01
LPC 18:2	21	1.656	[	9.54E-01	-	2.92E+00	]	21	1.537	[	1.11E+00	-	2.86E+00	]	0.93	266	2.61E-01	4.56E-01
12.98_592.5871n	21	0.133	[	7.59E-02	-	2.45E-01	]	21	0.144	[	9.52E-02	-	2.64E-01	]	1.08	177	2.83E-01	4.81E-01
FA 16:0	21	1.092	[	8.91E-01	-	1.44E+00	]	21	1.126	[	9.47E-01	-	1.35E+00	]	1.03	179	3.06E-01	5.07E-01
10.40_661.5027n	21	0.198	[	9.73E-02	-	2.31E-01	]	21	0.177	[	1.37E-01	-	2.40E-01	]	0.99	252	4.39E-01	7.10E-01
PI 36:1	21	0.092	[	5.98E-02	-	1.53E-01	]	21	0.101	[	4.85E-02	-	2.19E-01	]	1.09	191	4.70E-01	7.40E-01
5.98_302.2212n	21	0.050	[	2.50E-02	-	8.72E-02	]	21	0.059	[	2.46E-02	-	8.21E-02	]	1.06	193	5.01E-01	7.70E-01
13.40_785.6497m/z	21	0.062	[	3.17E-02	-	8.56E-02	]	21	0.058	[	3.28E-02	-	1.00E-01	]	0.98	245	5.50E-01	8.06E-01
6.72_312.2888n	21	0.136	[	5.51E-02	-	2.70E-01	]	21	0.154	[	8.30E-02	-	2.26E-01	]	1.04	196	5.50E-01	8.06E-01
CerP 40:1;O2	21	0.027	[	2.04E-02	-	3.66E-02	]	21	0.029	[	1.85E-02	-	3.77E-02	]	1.01	197	5.67E-01	8.06E-01
PC 34:2	21	4.384	[	3.13E+00	-	7.58E+00	]	21	4.543	[	3.14E+00	-	5.47E+00	]	1.00	198	5.84E-01	8.06E-01
PC 38:4	21	1.349	[	9.73E-01	-	2.17E+00	]	21	1.440	[	9.77E-01	-	1.98E+00	]	1.00	199	6.01E-01	8.06E-01
ST 27:1;O;S	21	0.158	[	8.57E-02	-	2.59E-01	]	21	0.163	[	1.01E-01	-	2.30E-01	]	1.03	199	6.01E-01	8.06E-01
Hex2Cer 42:2;O2	21	0.036	[	2.29E-02	-	4.46E-02	]	21	0.036	[	2.29E-02	-	6.17E-02	]	1.07	200	6.18E-01	8.12E-01
5.91_436.2544n	21	0.244	[	2.01E-01	-	3.10E-01	]	21	0.241	[	1.95E-01	-	4.18E-01	]	1.06	201	6.36E-01	8.18E-01
Hex2Cer 32:1;O2	21	0.011	[	8.55E-03	-	1.87E-02	]	21	0.012	[	7.33E-03	-	1.65E-02	]	0.97	231	8.03E-01	9.37E-01
HexCer 42:1;O2	21	0.111	[	7.66E-02	-	1.81E-01	]	21	0.120	[	5.83E-02	-	1.82E-01	]	1.01	208	7.65E-01	9.37E-01
LPC 20:4	21	0.451	[	3.07E-01	-	6.17E-01	]	21	0.431	[	2.87E-01	-	8.00E-01	]	1.07	209	7.84E-01	9.37E-01
PC 36:2	21	3.407	[	2.56E+00	-	5.11E+00	]	21	3.627	[	2.35E+00	-	3.97E+00	]	0.98	210	8.03E-01	9.37E-01
SM 32:1;O2	21	0.070	[	4.31E-02	-	1.11E-01	]	21	0.068	[	4.00E-02	-	1.12E-01	]	0.97	234	7.46E-01	9.37E-01
HexCer 34:1;O2	21	0.054	[	3.65E-02	-	6.62E-02	]	21	0.056	[	2.91E-02	-	8.25E-02	]	1.02	213	8.62E-01	9.41E-01
LPE 18:2	21	0.020	[	1.16E-02	-	5.43E-02	]	21	0.019	[	7.80E-03	-	5.52E-02	]	0.99	230	8.23E-01	9.41E-01
PE 36:2	21	0.438	[	3.36E-01	-	7.43E-01	]	21	0.441	[	3.37E-01	-	5.99E-01	]	0.97	212	8.42E-01	9.41E-01
PI 40:3 [iso 2]	21	0.067	[	3.29E-02	-	1.43E-01	]	21	0.073	[	3.50E-02	-	1.44E-01	]	0.97	227	8.81E-01	9.41E-01
PI O-42:6	21	0.030	[	1.54E-02	-	7.52E-02	]	21	0.034	[	1.77E-02	-	7.06E-02	]	1.02	214	8.81E-01	9.41E-01
CerPE 36:1;O2	21	0.346	[	2.37E-01	-	4.25E-01	]	21	0.326	[	2.52E-01	-	4.43E-01	]	1.01	226	9.01E-01	9.46E-01
FA 20:4	21	0.036	[	1.94E-02	-	8.35E-02	]	21	0.040	[	1.47E-02	-	6.86E-02	]	0.96	217	9.40E-01	9.56E-01

PC 36:4	21	2.311	[	1.56E+00	-	3.49E+00	]	21	2.379	[	1.74E+00	-	3.95E+00	]	1.02	217	9.40E-01	9.56E-01
SM 42:1;O2	21	0.210	[	1.29E-01	-	3.76E-01	]	21	0.207	[	1.52E-01	-	2.90E-01	]	0.98	221	1.00E+00	1.00E+00

[1] The foldchange was calculated as x̄pku /x̄control.

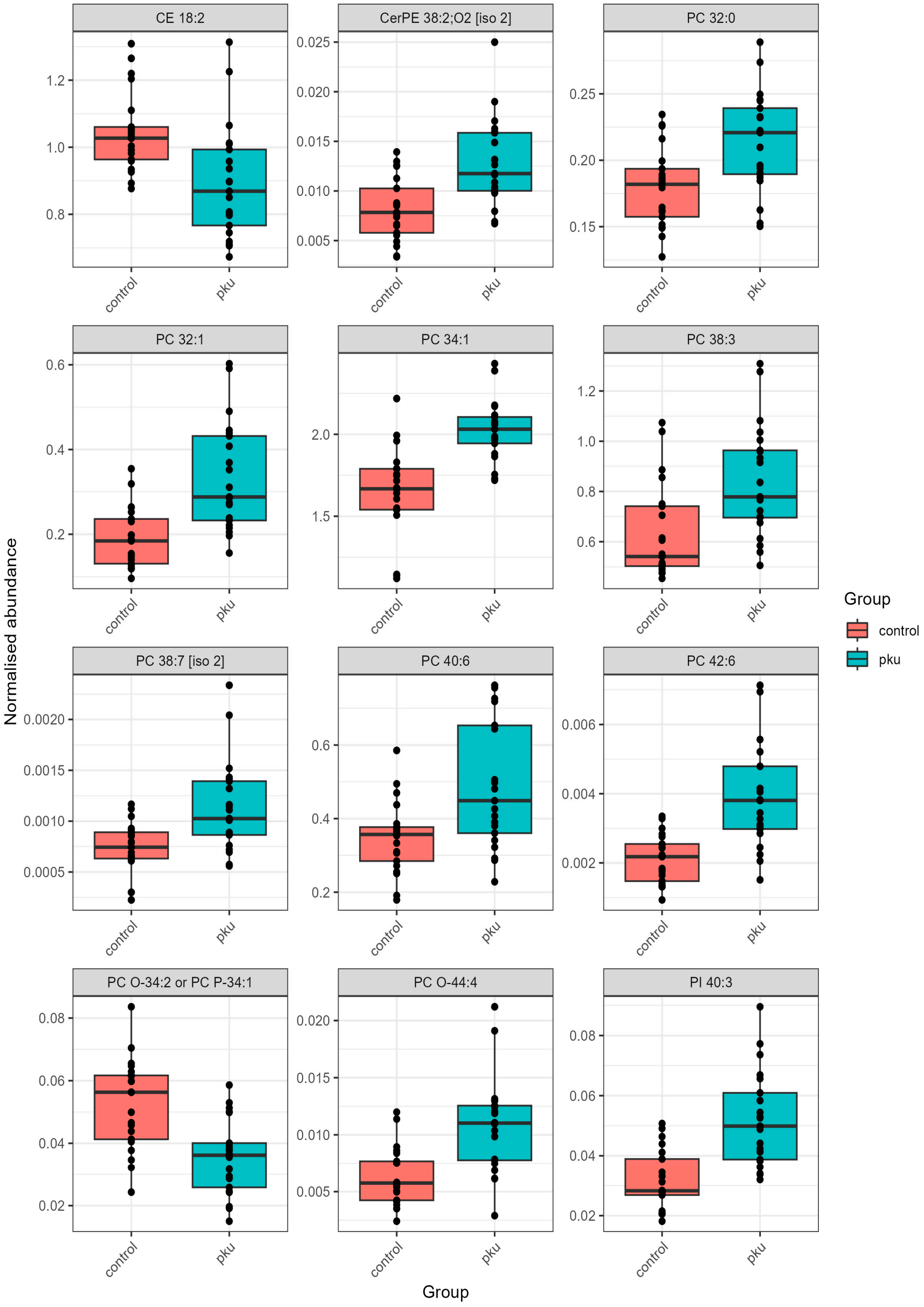

Figure S10. Statistically significant lipid features in ESI(+), at $p<0.05$.

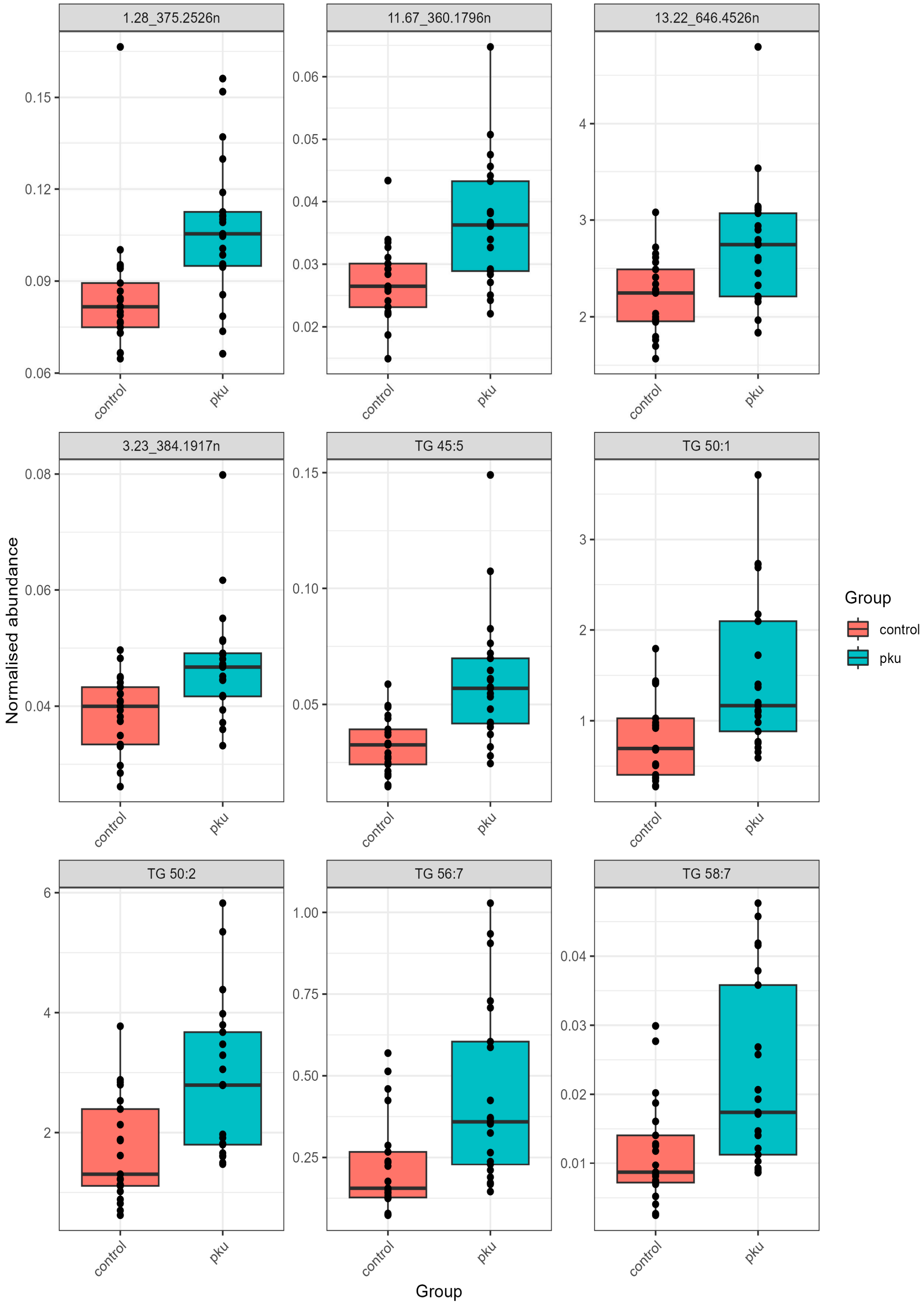

Figure S10 continued. Statistically significant lipid features in ESI(+), at $p<0.05$.

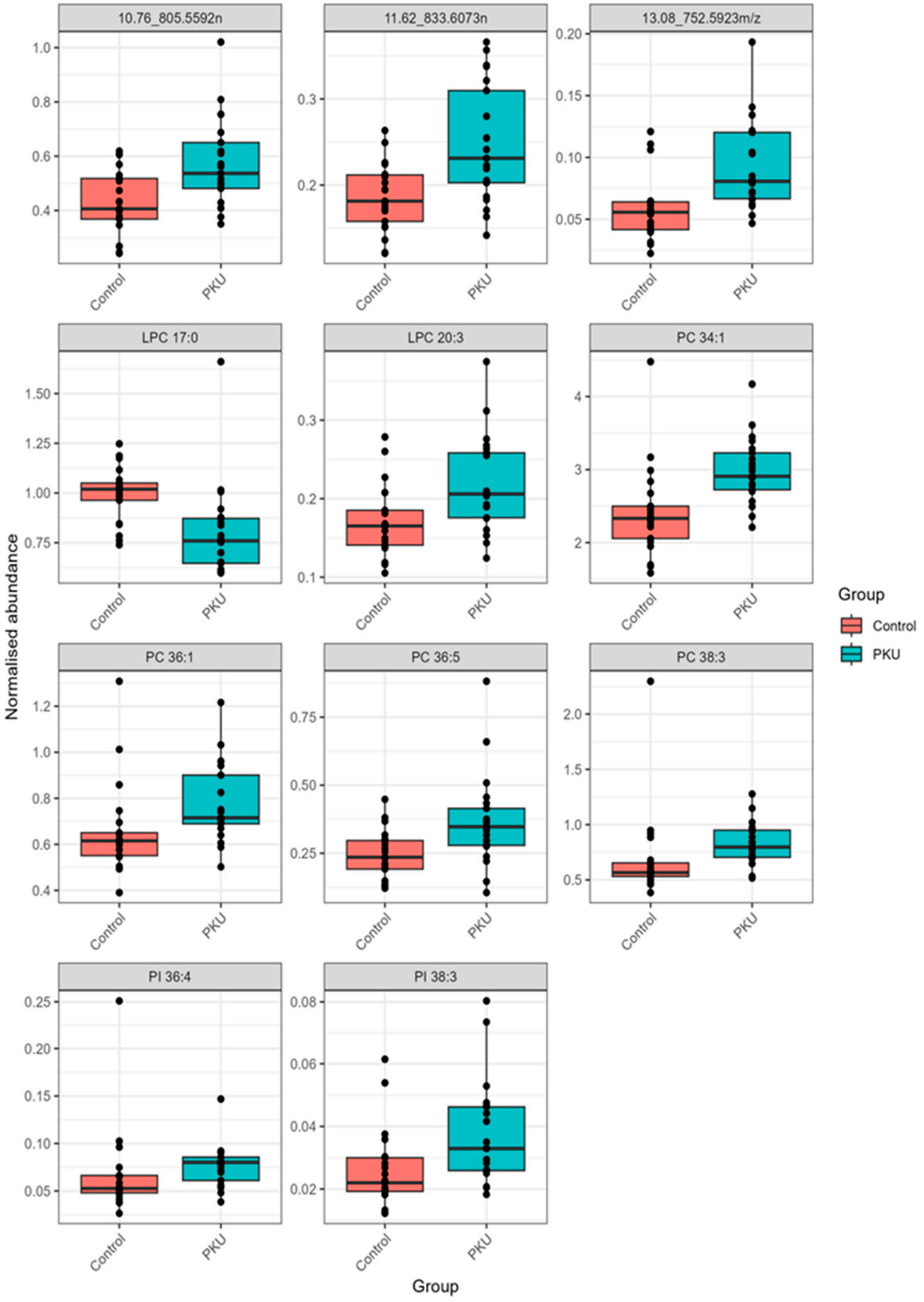

Figure S11. Statistically significant lipid features in ESI(-), at p<0.05.

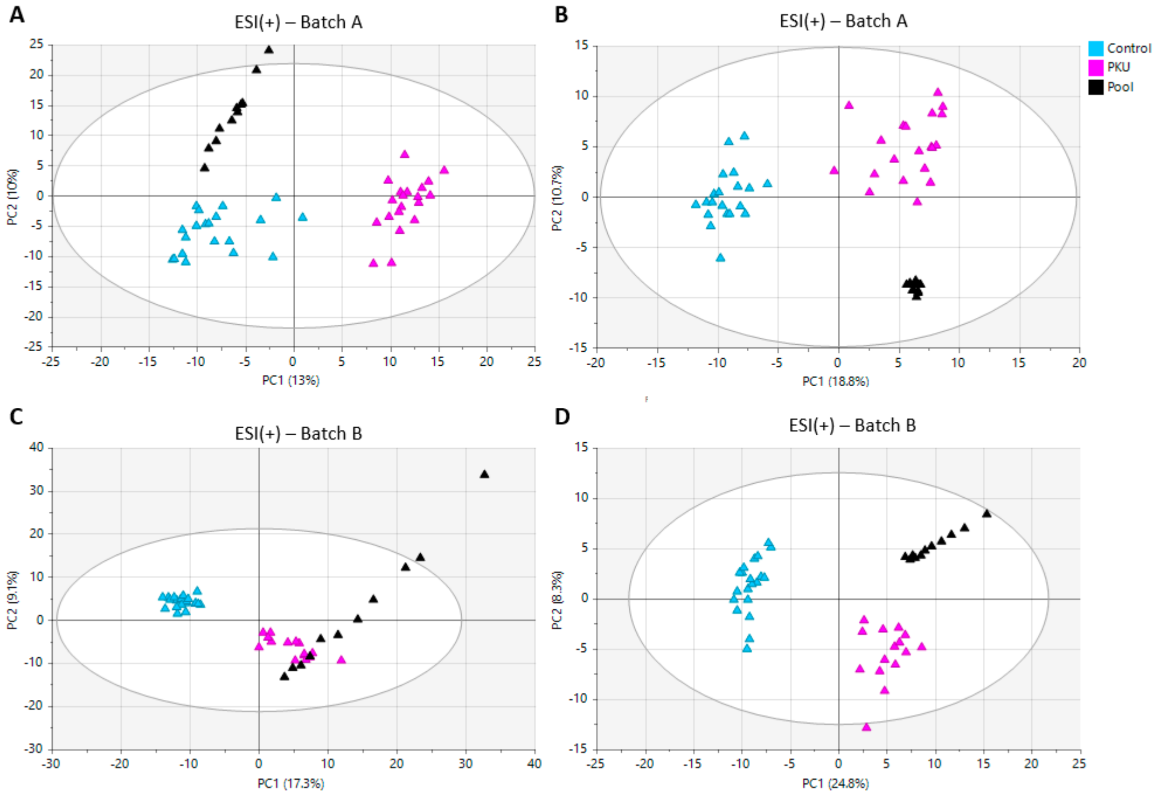

Figure S12. Metabolomics Normalization and Filtering Effects on the data obtained in ESI(+). The figure illustrates the impact of normalization and filtering on metabolomic data. OPLS-DA score plots of the different batches are shown. Panels A and B represent Batch A, while Panels C and D represent Batch B, with each panel showing the separation of metabolomic profiles between PKU patients (pink triangles), control group (blue triangles), and pooled quality control samples (black shapes). The following model parameters were obtained A) R2X 0.497 Q2 0.217 B) R2X 0.556 Q2 0.251 C) R2X 0.454 Q2 0.192 D) R2X 0.617 Q2 0.276

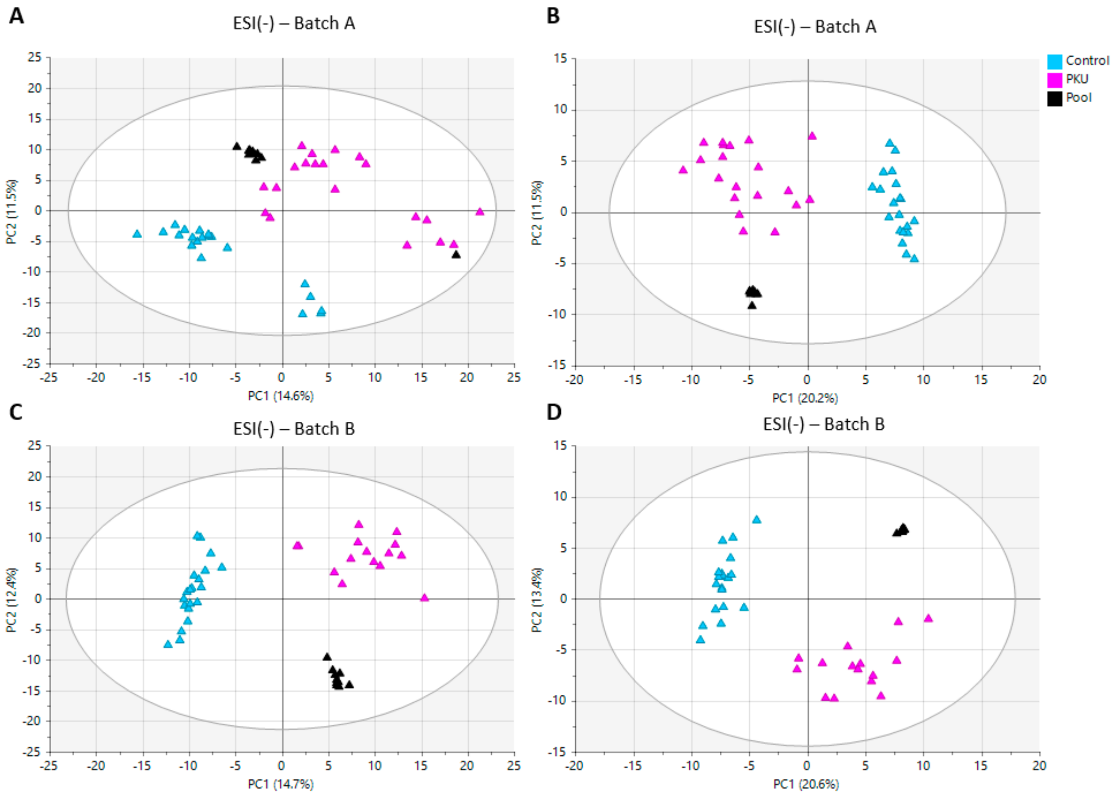

Figure S13. Metabolomics Normalization and Filtering Effects on the data obtained in ESI(-). PCA score plots demonstrating the effects of normalization and filtering on metabolomic data obtained in ESI(-) mode. Panels A and B depict Batch A, with cumulative explained variance (R2X(cum)) of 0.514 and predictive ability (Q2(cum)) of 0.26 for A, and R2X of 0.614 with Q2 of 0.215 for B. Panels C and D illustrate Batch B, with C showing an R2X of 0.499 and Q2 of 0.214, and D presenting R2X of 0.585 with Q2 of 0.26. Blue triangles represent control samples, pink triangles indicate PKU samples, and black triangles denote pooled samples across the two batches.

A

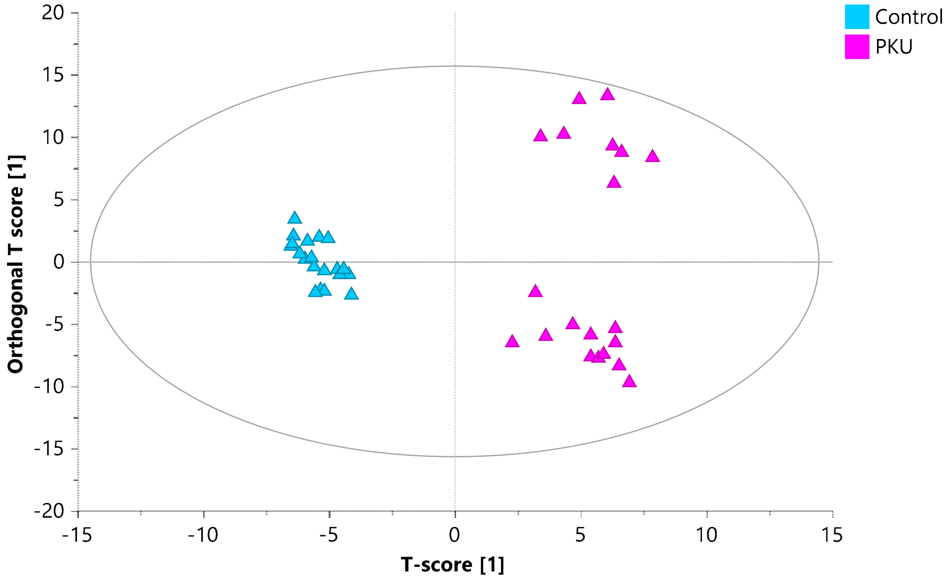

B

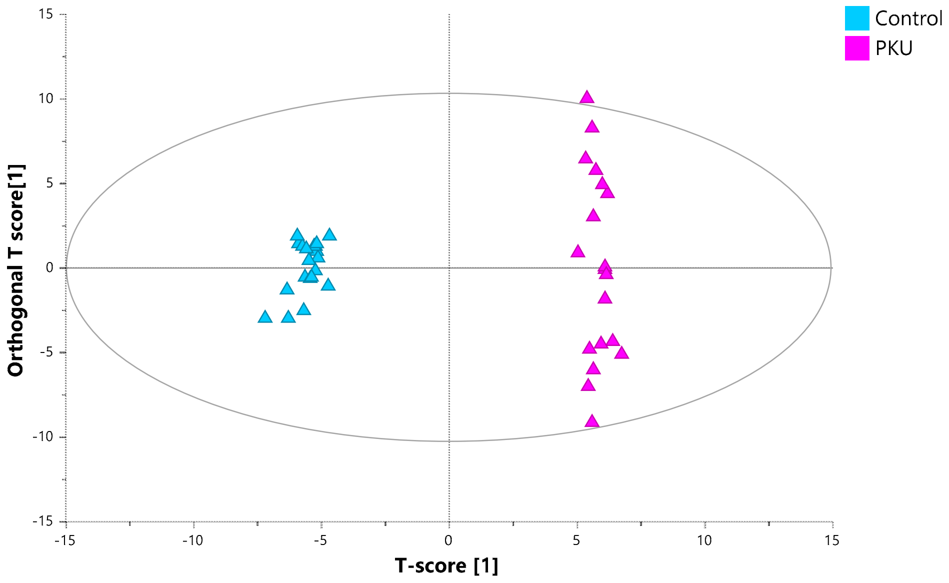

Figure S14. OPLS-DA results of metabolomic profiles in PKU patients compared to controls. The figure displays OPLSDA score plots for pairwise comparisons between PKU and control groups, utilizing metabolomics data from positive (A) and negative (B) ionization modes. Each point represents the metabolomic profile of an individual, with their metabolite abundances. For the positive ionization mode (ESI+), the model diagnostics are as follows: R2X = 0.394, R2Y = 0.959 and Q2(cum) = 0.926. B) R2X = 0.369, R2Y = 0.992 Q2(cum) = 0.95. Both models were statistically significant ($p<0.05$).

Table S10. Annotation metabolic features in ESI(+).

Feature ID	Retention time (min)	m/z	Adducts	Annotation	Mass Error (ppm)	Score	Isotope Similarity (%)	Level of annotation
0.50_132.0899n	0.50	133.0974	M+H-H2O, M+H	Ornithine	0.05	39.5	97	1
0.61_161.1074n	0.61	162.1147	M+H, M+Na, 2M+H					4
0.68_131.0696n	0.68	132.0769	M+H, M+Na, 2M+H	Creatine	0.99	39.5	98	1
0.71_186.0505n	0.71	187.0578	M+H, M+NH4					4
0.73_204.1231m/z	0.73	204.1231						4
0.94_229.1182m/z	0.94	229.1182	M+H	Pro-Hyp	-0.25	39.3	97	2
0.98_72.0809m/z	0.98	72.0809						4
1.03_186.1124m/z	1.03	186.1124						4
1.13_204.0211n	1.13	205.0288	M+H-H2O, M+H					4
1.17_244.0695n	1.17	267.0588	M+Na, M+K		-0.01	39.4	97	1
1.94_282.1195m/z	1.94	282.1195						2
2.23_86.0966m/z	2.23	86.0966						4
2.46_176.0949n	2.46	177.1022	M+H, M+Na	Serotonin or cotinine	-0.49	39.3	97	1
2.61_218.1388m/z	2.61	218.1388	M+H		0.57	39.1	96	1
2.96_175.0476m/z	2.96	175.0476	M+Na	N1-Methyl-4-pyridone-3-carboxamide	-1.15	39	97	1
3.07_153.0658m/z	3.07	153.0658		N1-Methyl-2-pyridone-5-carboxamide				1
3.66_165.0789n	3.66	188.0681	M+H-H2O, M+Na, M+K, M+2Na-H, 2M+H	Phe	-0.38	39.6	98	1
3.80_342.1560m/z	3.80	342.1560	M+H	Trp-His	-0.19	39	95	2
3.92_232.1543m/z	3.92	232.1543	M+H		-0.32	38.8	94	1
4.44_231.1702m/z	4.44	231.1702	M+H	Ile-Val/Leu-Val	-0.46	38.7	94	2
4.56_180.0648n	4.56	181.0720	M+H, M+Na	Paraxanthine	0.16	39.5	97	2
5.22_181.0720m/z	5.22	181.0720						4
5.40_180.0648n	5.40	181.0721	M+H, M+Na					4
5.77_179.0582n	5.77	180.0654	M+H-H2O, M+H, M+Na, M+K, M+2Na-H	Hippuric acid	-0.50	39.1	96	1
5.94_295.1289m/z	5.94	295.1289	M+H	Glutamyl-Phe	0.29	39.2	96	1
6.02_197.1283m/z	6.02	197.1283						2
6.29_195.0877m/z	6.29	195.0877						4
6.58_245.1859m/z	6.58	245.1859	M+H	Ile-Ile/Leu-Leu/Ile-Leu	-0.09	38.2	91	2
7.48_279.1702m/z	7.48	279.1702	M+H	Leu-Phe	-0.31	37.6	88	2
7.71_205.0738n	7.71	206.0811	M+H, M+Na	Indolelactic acid	-0.58	38.5	93	2
8.40_286.2014m/z	8.40	286.2014	M+H	5-Octenoylcarnitine (C8:1)	0.30	38.5	93	2
9.48_189.0788n	9.48	190.0860	M+H-H2O, M+H, M+Na, M+K	Indole-3-propionic acid	-1.18	37.9	91	2
9.59_291.2360m/z	9.59	291.2360						4
9.61_288.2171m/z	9.61	288.2171	M+H	Octanoylcarnitine (C8:0)	0.52	38.8	95	1
11.49_316.2485m/z	11.49	316.2485	M+H	Decanoylcarnitine (C10:0)	0.80	39	96	1
12.07_181.0857m/z	12.07	181.0857						4
12.11_210.1620n	12.11	228.1960	M+H-2H2O, M+H-H2O, M+H, M+NH4					4
12.11_267.1813n	12.11	250.1780	M+H-H2O, M+H					4

Feature ID	Retention time (min)	m/z	Adducts	Annotation	Mass Error (ppm)	Score	Isotope Similarity (%)	Level of annotation
13.63_279.1021m/z	13.63	279.1021	M+H		4.23	37.7	94	2
15.26_541.3189n	15.26	542.3262	M+H, M+Na	LPC 20:5	3.78	34.2	75	2
15.47_519.3335n	15.47	520.3408	M+H, M+Na	LPC 18:2 [iso 1]	2.01	38.3	94	2
15.53_329.2574n	15.53	352.2466	M+H-H2O, M+Na, M+K, 2M+H, 2M+Na					3
15.61_501.2869n	15.61	502.2942	M+H, M+Na	LPE 20:4	2.79	34.8	77	2
15.61_567.3337n	15.61	568.3410	M+H, M+Na, M+K	LPC 22:6	2.11	36.5	85	2
15.63_543.3335n	15.63	544.3408	M+H, M+Na	LPC 20:4 [iso 2]	1.84	38	92	2
15.64_519.3337n	15.64	520.3410	M+H, M+Na	LPC 18:2 [iso 2]	2.38	38.5	95	2
15.68_329.2575n	15.68	352.2467	M+Na, M+K, 2M+H, 2M+Na					4
15.70_496.3408m/z	15.70	496.3408	M+H	LPC 16:0 [iso1]	2.07	37.4	90	2
15.89_495.3339n	15.89	496.3412	M+H, M+Na, M+K	LPC 16:0 [iso 2]	2.86	38.7	97	2
15.90_411.3356n	15.90	434.3253	M+H, M+Na		1.82	38.4	94	2
15.90_545.3499n	15.90	546.3572	M+H, M+Na	LPC 20:3	3.25	37.2	90	2

Table S11. Annotation metabolic features in ESI(-).

Feature ID	Retention time (min)	m/z	Adducts	Annotation	Mass Error (ppm)	Score	Isotope Similarity (%)	Level of annotation
0.60_180.0640n	0.60	179.0567	M-H2O-H, M-H, 2M-H					4
0.60_298.1151m/z	0.60	298.1151						4
1.02_90.0279m/z	1.02	90.0279						4
2.97_151.0510m/z	2.97	151.0510	M-H	N1-Methyl-2-pyridone-5-carboxamide	-2.26	39.1	98	1
3.25_147.0448m/z	3.25	147.0448						4
3.25_329.1505m/z	3.25	329.1505						4
3.26_712.2200m/z	3.26	712.2200						4
3.76_326.1243m/z	3.76	326.1243	M-H	N-(1-Deoxy-1-fructosyl)phenylalanine	-0.66	39.4	98	1
3.81_221.0929m/z	3.81	221.0929	M-H	Gly-Phe	-1.21	37.3	88	2
3.86_165.0415m/z	3.86	165.0415	M-H	Methylxanthine	-1.54	39.2	98	2
4.23_218.1032m/z	4.23	218.1032	M-H	Panthothenic acid	-1.00	38.8	95	1
4.49_151.0398m/z	4.49	151.0398	M-H		-1.53	39.4	99	1
4.68_174.0592m/z	4.68	174.0592						4
4.69_280.1058n	4.69	279.0983	M-H2O-H, M-H	Asp-Phe	-0.53	38.9	95	2
4.84_211.0585m/z	4.84	211.0585						4
5.11_179.0573m/z	5.11	179.0573						4
5.29_179.0574m/z	5.29	179.0574	M-H	Paraxanthine	-0.31	39.7	99	2
5.53_294.1216n	5.53	293.1143	M-H, M+Na-2H	Glu-Phe	-0.01	39.5	98	1
5.76_146.0688n	5.76	145.0615	M-H2O-H, M-H	Ala-Gly	-2.62	38.8	97	2
5.76_263.1038m/z	5.76	263.1038	M-H	Phenylacetylglutamine	0.08	39.5	97	2
6.09_163.0400m/z	6.09	163.0400	M-H	Phenylpyruvic acid	-0.61	39.6	99	1
6.28_159.0659m/z	6.28	159.0659						4
6.35_151.0399m/z	6.35	151.0399	M-H	Hydroxyphenylacetic acid	-0.97	39.7	99	1
7.02_151.0397m/z	7.02	151.0397						4
7.07_166.0609n	7.07	165.0536	M-H2O-H, M-H, 2M-H	Phenyl lactic acid				1
7.40_144.0452m/z	7.40	144.0452						4
7.70_748.3751n	7.70	747.3678	M-H, M+Na-2H					4
7.71_144.0453m/z	7.71	144.0453	M-H	Indole-3-carboxaldehyde	-1.33	39.4	99	2
7.91_236.0926m/z	7.91	236.0926	M-H	N-lactoyl Phe	-0.91	37.8	90	1
7.93_91.0551m/z	7.93	91.0551						4

Feature ID	Retention time (min)	m/z	Adducts	Annotation	Mass Error (ppm)	Score	Isotope Similarity (%)	Level of annotation
8.16_199.0061m/z	8.16	199.0061						4
8.75_429.1587m/z	8.75	429.1587						4
8.80_150.0018m/z	8.80	150.0018						1
8.88_201.0229m/z	8.88	201.0229						4
9.35_165.9789m/z	9.35	165.9789						4
9.50_149.0606m/z	9.50	149.0606						4
9.86_159.1025m/z	9.86	159.1025						4
9.87_209.0818m/z	9.87	209.0818						4
10.24_385.1687m/z	10.24	385.1687						4
10.38_385.1688m/z	10.38	385.1688						4
10.71_163.0762m/z	10.71	163.0762						4
10.86_541.2650m/z	10.86	541.2650	M-H	Cortolone-3-glucuronide	-0.74	38.9	95	2
11.13_539.2494m/z	11.13	539.2494						4
11.16_383.1533m/z	11.16	383.1533						4
12.05_143.1075m/z	12.05	143.1075						4
12.05_204.1360n	12.05	225.1107	M-H2O-H, M+Na-2H					4
12.26_187.1336m/z	12.26	187.1336						4
12.31_165.0917m/z	12.31	165.0917						4
12.38_331.1760m/z	12.38	331.1760	M-H	Trp-Lys	-4.65	37.3	92	2
12.81_367.1589m/z	12.81	367.1589						4
13.20_369.1740m/z	13.20	369.1740						4
13.26_193.1232m/z	13.26	193.1232	M-H	FA 12:3	-1.27	39.4	99	2
13.42_169.1230m/z	13.42	169.1230						4
13.56_444.9728m/z	13.56	444.9728						4
13.74_511.2909m/z	13.74	511.2909						4
14.03_369.1742m/z	14.03	369.1742						4
14.32_448.3067m/z	14.32	448.3067	M+FA-H	N-Palmitoyl Phe	-0.29	39.5	98	2
15.30_578.3462m/z	15.30	578.3462	M+FA-H	LPE 22:2	-0.21	39.7	99	2
15.32_602.3458m/z	15.32	602.3458						4
15.34_500.2780m/z	15.34	500.2780	M-H	LPE 20:4	-0.54	39.3	97	2

Feature ID	Retention time (min)	m/z	Adducts	Annotation	Mass Error (ppm)	Score	Isotope Similarity (%)	Level of annotation
15.34_524.2777m/z	15.34	524.2777	M-H	LPE 22:6	-0.99	39.2	97	2
15.54_554.3460m/z	15.54	554.3460	M+FA-H	LPE 20:0 [iso 1]	-0.67	39.5	98	2
15.71_554.3464m/z	15.71	554.3464	M+FA-H	LPE 20:0 [iso 2]	0.21	39.9	100	2
15.73_604.3615m/z	15.73	604.3615						4
15.87_580.3620m/z	15.87	580.3620	M+FA-H	LPE 22:1	0.09	39.6	98	2
15.91_339.2332m/z	15.91	339.2332						4
15.99_277.2173m/z	15.99	277.2173	M-H	FA 18:3	-0.18	39.3	97	2

Table S12. Results from the Wilcoxon Rank Sum Test comparing control and PKU groups within the ESI(-) metabolomics dataset.

	Control				PKU								
Feature	n	Median	Min	Max	n	Median	Min	Max	FC[1]	U	*p*-value	*p*-value adjusted	Significant
10.71_163.0762m/z	20	0.130	[1.17E-01	- 1.78E-01]	35	1.273	[6.40E-01	- 1.99E+00]	9.83	0	3.96E-15	2.21E-14	*
12.05_143.1075m/z	20	0.338	[2.53E-01	- 4.41E-01]	35	9.605	[7.39E+00	- 1.72E+01]	30.09	0	3.96E-15	2.21E-14	*
15.91_339.2332m/z	20	0.012	[6.01E-03	- 2.80E-02]	35	17.284	[7.05E+00	- 2.97E+01]	1267.34	0	3.96E-15	2.21E-14	*
3.25_147.0448m/z	20	0.885	[7.52E-01	- 1.19E+00]	35	5.011	[2.20E+00	- 1.09E+01]	5.82	0	3.96E-15	2.21E-14	*
3.25_329.1505m/z	20	0.427	[2.87E-01	- 6.47E-01]	35	4.682	[1.71E+00	- 1.22E+01]	11.93	0	3.96E-15	2.21E-14	*
3.26_712.2200m/z	20	1.836	[1.33E+00	- 3.01E+00]	35	9.599	[4.02E+00	- 2.27E+01]	5.46	0	3.96E-15	2.21E-14	*
4.68_174.0592m/z	20	0.004	[3.74E-03	- 5.40E-03]	35	0.921	[4.77E-02	- 2.43E+00]	215.40	0	3.96E-15	2.21E-14	*
Glu-Phe	20	0.082	[4.23E-02	- 1.26E-01]	35	2.861	[7.38E-01	- 8.38E+00]	36.58	0	3.96E-15	2.21E-14	*
Phenylpyruvic acid	20	0.014	[1.12E-02	- 1.64E-02]	35	3.348	[2.02E-01	- 4.52E+01]	455.80	0	3.96E-15	2.21E-14	*
Phenyl lactic acid	20	0.404	[1.76E-01	- 9.67E-01]	35	18.560	[2.06E+00	- 1.13E+02]	58.48	0	3.96E-15	2.21E-14	*
N-lactoyl Phe	20	0.004	[3.60E-04	- 3.17E-02]	35	0.362	[4.82E-02	- 1.28E+00]	79.90	0	3.96E-15	2.21E-14	*
8.80_150.0018m/z	20	0.074	[5.00E-02	- 2.22E-01]	35	5.641	[2.21E+00	- 8.21E+00]	57.25	0	3.96E-15	2.21E-14	*
p-Hydroxyphenylacetic acid	20	0.010	[3.10E-03	- 5.36E-02]	35	2.757	[4.91E-02	- 1.21E+01]	227.92	1	7.92E-15	4.08E-14	*
12.05_204.1360n	20	0.638	[1.86E-01	- 2.02E+00]	35	1.803	[1.26E+00	- 4.18E+00]	2.95	26	4.63E-11	2.21E-10	*
7.40_144.0452m/z	20	0.254	[1.55E-01	- 4.49E-01]	35	0.769	[1.60E-01	- 2.00E+00]	3.16	45	2.01E-09	8.99E-09	*
Gly-Phe	20	0.025	[2.67E-03	- 2.81E-01]	35	0.210	[4.00E-03	- 6.50E-01]	5.19	59	1.93E-08	8.08E-08	*
LPE 20:4	20	1.533	[6.70E-01	- 5.13E+00]	35	0.568	[1.00E-01	- 1.85E+00]	0.35	639	2.60E-08	1.02E-07	*
7.93_91.0551m/z	20	0.086	[1.32E-02	- 3.71E-01]	35	0.440	[4.42E-02	- 1.92E+00]	5.20	64	4.01E-08	1.49E-07	*
N-(1-Deoxy-1-fructosyl)-Phe	20	0.037	[3.14E-02	- 4.58E-02]	35	0.905	[2.30E-02	- 4.70E+00]	36.87	80	3.39E-07	1.19E-06	*
4.49_151.0398m/z	20	0.004	[3.42E-03	- 5.04E-03]	35	0.384	[2.82E-03	- 2.29E+00]	138.75	83	4.90E-07	1.64E-06	*
Panthothenic acid	20	0.186	[1.15E-01	- 6.02E-01]	35	0.399	[1.39E-01	- 8.19E-01]	1.90	84	5.53E-07	1.76E-06	*
Indole-3-carboxaldehyde	20	0.593	[1.88E-01	- 1.55E+00]	35	1.554	[4.52E-01	- 4.94E+00]	2.74	87	7.91E-07	2.41E-06	*
9.35_165.9789m/z	20	0.792	[5.73E-01	- 9.72E-01]	35	1.075	[5.26E-01	- 1.69E+00]	1.32	144	1.96E-04	5.71E-04	*
8.88_201.0229m/z	20	0.444	[2.34E-02	- 3.08E+01]	35	0.067	[7.31E-04	- 7.44E-01]	0.08	555	2.12E-04	5.92E-04	*
15.73_604.3615m/z	20	0.946	[4.09E-01	- 1.95E+00]	35	1.373	[6.40E-01	- 3.34E+00]	1.55	156	4.89E-04	1.31E-03	*
Phenylacetylglutamine	20	3.559	[1.61E+00	- 1.02E+01]	35	6.931	[2.08E+00	- 1.56E+01]	1.81	157	5.27E-04	1.36E-03	*

				Control								PKU								
Feature	n	Median		Min		Max		n	Median		Min		Max		FC[I]	U	*p*-value	*p*-value adjusted	Significant
Ala-Gly	20	0.209	[	7.00E-02	-	6.62E-01	]	35	0.493	[	3.13E-02	-	1.13E+00	]	1.91	164	8.67E-04	2.15E-03	*
15.32_602.3458m/z	20	1.387	[	6.10E-01	-	2.43E+00	]	35	0.818	[	2.20E-01	-	2.04E+00	]	0.67	534	9.95E-04	2.38E-03	*
10.38_385.1688m/z	20	0.551	[	3.71E-02	-	2.00E+00	]	35	1.231	[	7.11E-02	-	4.07E+00	]	2.04	170	1.31E-03	3.02E-03	*
13.74_511.2909m/z	20	0.196	[	1.33E-02	-	6.85E-01	]	35	0.493	[	6.01E-03	-	1.02E+00	]	2.10	172	1.49E-03	3.22E-03	*
N1-Methyl-2-pyridone-5-carboxamide	20	0.120	[	2.61E-03	-	3.23E-01	]	35	0.240	[	2.48E-03	-	6.53E-01	]	1.98	172	1.49E-03	3.22E-03	*
LPE 22:1	20	4.913	[	2.12E+00	-	1.04E+01	]	35	6.296	[	1.80E+00	-	1.02E+01	]	1.30	173	1.59E-03	3.33E-03	*
LPE 20:0 [iso 2]	20	6.638	[	3.75E+00	-	1.25E+01	]	35	8.380	[	2.77E+00	-	1.39E+01	]	1.31	175	1.81E-03	3.68E-03	*
9.87_209.0818m/z	20	0.224	[	4.44E-03	-	2.14E+01	]	35	0.040	[	3.11E-04	-	7.14E+00	]	0.16	522	2.20E-03	4.33E-03	*
LPE 22:6	20	0.616	[	1.78E-01	-	1.43E+00	]	35	0.358	[	1.38E-02	-	8.96E-01	]	0.55	518	2.83E-03	5.41E-03	*
LPE 20:0 [iso 1]	20	0.715	[	3.54E-01	-	2.04E+00	]	35	1.137	[	1.83E-01	-	2.91E+00	]	1.47	187	3.83E-03	7.13E-03	*
13.20_369.1740m/z	20	2.908	[	6.29E-01	-	7.70E+00	]	35	5.066	[	3.59E-01	-	2.42E+01	]	2.03	199	7.64E-03	1.38E-02	*
11.16_383.1533m/z	20	1.828	[	4.67E-01	-	4.74E+00	]	35	3.192	[	7.08E-01	-	9.07E+00	]	1.71	200	8.07E-03	1.42E-02	*
12.26_187.1336m/z	20	0.371	[	8.87E-02	-	1.24E+00	]	35	0.598	[	2.90E-02	-	1.56E+00	]	1.53	202	9.00E-03	1.55E-02	*
11.13_539.2494m/z	20	0.595	[	7.14E-02	-	1.47E+00	]	35	0.841	[	4.21E-02	-	2.15E+00	]	1.48	204	1.00E-02	1.68E-02	*
FA 18:3	20	0.845	[	5.77E-02	-	5.03E+00	]	35	1.777	[	2.54E-01	-	8.17E+00	]	1.67	216	1.86E-02	3.03E-02	*
10.24_385.1687m/z	20	0.832	[	2.20E-01	-	1.77E+00	]	35	1.137	[	2.81E-01	-	3.05E+00	]	1.42	219	2.15E-02	3.35E-02	*
8.16_199.0061m/z	20	1.564	[	2.29E-03	-	1.51E+01	]	35	0.357	[	3.40E-04	-	5.78E+00	]	0.33	481	2.15E-02	3.35E-02	*
12.31_165.0917m/z	20	0.238	[	2.62E-02	-	4.78E-01	]	35	0.301	[	3.40E-02	-	1.19E+00	]	1.60	229	3.42E-02	5.20E-02	
14.03_369.1742m/z	20	11.090	[	2.31E+00	-	3.34E+01	]	35	16.926	[	1.16E+00	-	6.85E+01	]	1.44	247	7.27E-02	1.08E-01	
9.86_159.1025m/z	20	0.568	[	3.13E-01	-	1.17E+00	]	35	0.734	[	1.26E-01	-	1.60E+00	]	1.26	249	7.86E-02	1.14E-01	
12.81_367.1589m/z	20	34.660	[	9.32E+00	-	5.47E+01	]	35	42.166	[	1.57E+01	-	1.05E+02	]	1.28	256	1.02E-01	1.46E-01	
0.60_298.1151m/z	20	1.191	[	7.46E-01	-	1.67E+00	]	35	0.765	[	2.96E-01	-	2.32E+00	]	0.83	440	1.18E-01	1.61E-01	
LPE 22:2	20	2.955	[	1.83E+00	-	4.57E+00	]	35	2.485	[	7.62E-01	-	6.41E+00	]	0.87	440	1.18E-01	1.61E-01	
6.28_159.0659m/z	20	0.098	[	6.37E-02	-	1.95E-01	]	35	0.087	[	2.03E-03	-	3.74E-01	]	0.85	437	1.31E-01	1.76E-01	
8.75_429.1587m/z	20	0.115	[	5.07E-03	-	8.23E-01	]	35	0.007	[	4.21E-03	-	9.51E-01	]	0.99	434	1.45E-01	1.91E-01	
FA 12:3	20	0.348	[	1.94E-02	-	8.61E-01	]	35	0.470	[	8.64E-02	-	1.16E+00	]	1.34	278	2.13E-01	2.74E-01	
0.60_180.0640n	20	2.512	[	2.12E+00	-	3.73E+00	]	35	2.078	[	1.34E+00	-	4.15E+00	]	0.95	420	2.26E-01	2.86E-01	

		Control									PKU										
Feature	n	Median		Min		Max		n	Median		Min		Max		FC[I]	U	*p*-value	*p*-value adjusted	Significant
Cortolone-3-glucuronide	20	0.491	[	8.00E-03	-	1.27E+00	]	35	0.416	[	1.24E-02	-	2.00E+00	]	1.34	284	2.54E-01	3.15E-01	
13.56_444.9728m/z	20	31.786	[	1.73E+01	-	4.81E+01	]	35	1.478	[	1.06E+00	-	8.00E+01	]	0.76	415	2.62E-01	3.19E-01	
Trp-Lys	20	0.319	[	2.81E-02	-	2.76E+00	]	35	0.242	[	1.96E-04	-	6.92E+00	]	1.30	414	2.69E-01	3.22E-01	
N-Palmitoyl Phe	20	0.603	[	6.56E-03	-	3.30E+00	]	35	0.733	[	5.79E-03	-	4.60E+00	]	1.41	289	2.93E-01	3.44E-01	
4.84_211.0585m/z	20	0.287	[	1.66E-01	-	4.56E-01	]	35	0.220	[	4.63E-02	-	5.83E-01	]	0.97	409	3.09E-01	3.57E-01	
13.42_169.1230m/z	20	0.490	[	1.29E-01	-	1.31E+00	]	35	0.589	[	6.65E-02	-	1.39E+00	]	1.17	292	3.17E-01	3.60E-01	
Asp-Phe	20	0.442	[	6.18E-03	-	2.22E+00	]	35	0.287	[	7.60E-03	-	1.18E+00	]	0.73	392	4.71E-01	5.26E-01	
Methylxanthine	20	0.382	[	4.75E-03	-	1.78E+00	]	35	0.423	[	1.46E-02	-	3.04E+00	]	1.31	326	6.84E-01	7.51E-01	
1.02_90.0279m/z	20	3.985	[	2.72E+00	-	5.30E+00	]	35	4.868	[	1.72E+00	-	7.20E+00	]	1.06	330	7.35E-01	7.95E-01	
5.11_179.0573m/z	20	0.332	[	1.87E-03	-	1.80E+00	]	35	0.380	[	9.19E-06	-	2.14E+00	]	1.14	336	8.15E-01	8.67E-01	
7.70_748.3751n	20	0.642	[	1.68E-02	-	2.41E+00	]	35	0.663	[	1.29E-02	-	3.35E+00	]	1.05	363	8.28E-01	8.67E-01	
Paraxanthine	20	1.293	[	2.63E-03	-	6.19E+00	]	35	1.296	[	1.33E-01	-	6.65E+00	]	1.04	345	9.38E-01	9.52E-01	
9.50_149.0606m/z	20	0.828	[	6.54E-03	-	4.13E+00	]	35	1.073	[	3.50E-03	-	6.94E+00	]	1.09	355	9.38E-01	9.52E-01	
7.02_151.0397m/z	20	0.171	[	1.11E-03	-	3.11E+00	]	35	0.044	[	9.28E-04	-	9.92E+00	]	1.29	351	9.93E-01	9.93E-01	

[I] The foldchange was calculated as $\bar{x}_{pku}/\bar{x}_{control}$.

Table S13. Results from the Wilcoxon Rank Sum Test comparing control and PKU groups within the ESI(+) metabolomics dataset.

	Control				PKU								
Feature	*n*	Median	Min	Max	*n*	Median	Min	Max	FC[1]	U	*p*-value	*p*-value adjusted	Significant
Phe	20	0.288	[2.07E-01	- 4.30E-01]	35	2.440	[8.09E-01	- 4.51E+00]	8.696	0	3.96E-15	1.01E-13	*
Glu-Phe	20	0.019	[9.39E-04	- 4.20E-02]	35	0.849	[2.36E-01	- 2.41E+00]	44.410	0	3.96E-15	1.01E-13	*
Leu-Phe	20	0.001	[6.87E-04	- 3.21E-02]	35	0.042	[2.64E-03	- 7.71E-01]	22.014	17	4.80E-12	8.16E-11	*
Indolelactic acid	20	0.058	[2.93E-02	- 1.34E-01]	35	0.281	[4.73E-02	- 1.01E+00]	5.553	30	1.12E-10	1.43E-09	*
1.03_186.1124m/z	20	0.210	[1.50E-01	- 4.77E-01]	35	0.050	[1.06E-04	- 7.31E-01]	0.461	599	3.78E-06	3.86E-05	*
1.94_282.1195m/z	20	0.120	[9.97E-02	- 1.34E-01]	35	0.133	[1.05E-01	- 2.00E-01]	1.149	104	5.18E-06	4.41E-05	*
Ile-Val/Leu-Val	20	0.063	[3.10E-02	- 2.92E-01]	35	0.141	[4.46E-02	- 9.97E-01]	2.214	129	5.62E-05	3.91E-04	*
Ile-Ile/Leu-Leu/Ile-Leu	20	0.104	[4.22E-02	- 1.96E-01]	35	0.163	[7.59E-02	- 4.11E-01]	1.621	130	6.13E-05	3.91E-04	*
Trp-His	20	0.046	[3.53E-03	- 1.10E-01]	35	0.087	[3.45E-02	- 2.55E-01]	2.032	140	1.42E-04	8.06E-04	*
Ornithine	20	0.372	[1.88E-01	- 6.47E-01]	35	0.272	[1.93E-01	- 5.56E-01]	0.746	557	1.81E-04	9.24E-04	*
LPC 20:3	20	0.306	[8.25E-02	- 1.52E+00]	35	0.679	[1.52E-01	- 6.32E+00]	2.161	157	5.27E-04	2.44E-03	*
N1-Methyl-2-pyridone-5-carboxamide	20	0.697	[2.53E-01	- 1.48E+00]	35	1.030	[2.13E-01	- 2.38E+00]	1.468	199	7.64E-03	3.25E-02	*
Pro-Hyp	20	0.122	[5.16E-02	- 2.56E-01]	35	0.184	[1.08E-01	- 7.10E-01]	1.462	206	1.12E-02	4.37E-02	*
Indole-3-propionic acid	20	0.097	[1.21E-02	- 4.92E-01]	35	0.212	[3.34E-03	- 2.19E+00]	2.568	210	1.37E-02	5.00E-02	
LPC 22:6	20	0.254	[1.01E-01	- 1.44E+00]	35	0.469	[6.28E-02	- 4.61E+00]	1.733	212	1.52E-02	5.09E-02	
N1-Methyl-4-pyridone-3-carboxamide	20	0.091	[1.07E-02	- 2.12E-01]	35	0.148	[8.96E-03	- 4.43E-01]	1.663	213	1.60E-02	5.09E-02	
0.73_204.1231m/z	20	1.772	[9.37E-01	- 2.83E+00]	35	2.134	[9.22E-01	- 3.46E+00]	1.231	218	2.05E-02	6.14E-02	
1.17_244.0695n	20	0.246	[1.90E-01	- 3.32E-01]	35	0.290	[1.60E-01	- 5.32E-01]	1.201	223	2.60E-02	7.35E-02	
LPC 20:5	20	0.057	[9.76E-05	- 3.92E-01]	35	0.105	[4.53E-04	- 8.12E-01]	1.608	231	3.73E-02	1.00E-01	
9.59_291.2360m/z	20	2.480	[2.21E+00	- 2.67E+00]	35	2.737	[2.10E+00	- 3.17E+00]	1.096	242	5.96E-02	1.52E-01	
LPC 16:0 [iso 2]	20	4.496	[1.97E+00	- 2.14E+01]	35	6.167	[1.17E+00	- 3.97E+01]	1.318	246	6.99E-02	1.70E-01	
LPC 16:0 [iso1]	20	0.196	[7.55E-02	- 1.22E+00]	35	0.294	[4.48E-02	- 2.80E+00]	1.435	248	7.56E-02	1.75E-01	
Creatine	20	2.685	[1.04E+00	- 5.78E+00]	35	2.199	[7.51E-01	- 7.24E+00]	0.863	443	1.06E-01	2.25E-01	
0.71_186.0505n	20	0.702	[2.94E-01	- 9.85E-01]	35	0.610	[1.75E-01	- 1.20E+00]	0.876	444	1.02E-01	2.25E-01	
LPE 20:4	20	0.034	[1.81E-02	- 1.40E-01]	35	0.047	[1.31E-02	- 1.71E-01]	1.195	262	1.27E-01	2.58E-01	
6.02_197.1283m/z	20	0.158	[5.91E-02	- 5.25E-01]	35	0.124	[2.66E-02	- 3.71E-01]	0.782	435	1.40E-01	2.75E-01	
3.92_232.1543m/z	20	0.168	[6.63E-02	- 2.01E+00]	35	0.190	[8.91E-02	- 6.65E-01]	0.832	272	1.77E-01	3.34E-01	
12.07_181.0857m/z	20	0.086	[4.17E-02	- 2.99E-01]	35	0.078	[1.08E-04	- 3.20E-01]	0.722	416	2.54E-01	4.47E-01	
15.53_329.2574n	20	13.000	[1.13E+01	- 1.39E+01]	35	10.163	[8.14E+00	- 1.73E+01]	0.914	416	2.54E-01	4.47E-01	
Decanoylcarnitine (C10:0)	20	0.413	[1.12E-01	- 1.60E+00]	35	0.529	[1.33E-01	- 1.70E+00]	1.100	289	2.93E-01	4.97E-01	
13.63_279.1021m/z	20	10.377	[1.00E+01	- 1.13E+01]	35	8.715	[8.16E+00	- 1.37E+01]	0.939	407	3.26E-01	5.36E-01	

				Control							PKU								
Feature	*n*	Median		Min		Max		*n*	Median		Min		Max		FC[I]	U	*p*-value	*p*-value adjusted	Significant
0.61_161.1074n	20	9.388	[	8.01E+00	-	1.11E+01	]	35	10.171	[	6.65E+00	-	1.24E+01	]	1.033	298	3.71E-01	5.64E-01	
15.68_329.2575n	20	16.616	[	1.33E+01	-	1.78E+01	]	35	11.615	[	7.47E+00	-	2.35E+01	]	0.895	400	3.90E-01	5.64E-01	
15.90_411.3356n	20	0.396	[	3.57E-01	-	4.25E-01	]	35	0.585	[	1.59E-01	-	6.51E-01	]	1.072	300	3.90E-01	5.64E-01	
Hippuric acid	20	0.571	[	3.25E-01	-	1.39E+00	]	35	0.463	[	3.91E-02	-	2.69E+00	]	1.103	398	4.09E-01	5.64E-01	
6.29_195.0877m/z	20	1.447	[	1.32E-01	-	1.03E+01	]	35	1.927	[	1.56E-01	-	1.42E+01	]	1.060	300	3.90E-01	5.64E-01	
Octanoylcarnitine (C8:0)	20	0.356	[	1.22E-01	-	1.63E+00	]	35	0.491	[	1.44E-01	-	1.58E+00	]	1.043	301	3.99E-01	5.64E-01	
12.11_210.1620n	20	1.330	[	6.49E-01	-	2.69E+00	]	35	1.524	[	6.53E-01	-	3.90E+00	]	1.193	304	4.29E-01	5.76E-01	
LPC 18:2 [iso 2]	20	5.681	[	2.36E+00	-	1.91E+01	]	35	6.654	[	1.40E+00	-	3.57E+01	]	1.108	312	5.15E-01	6.74E-01	
12.11_267.1813n	20	0.933	[	4.67E-01	-	1.69E+00	]	35	0.963	[	4.37E-01	-	2.40E+00	]	1.156	318	5.85E-01	6.93E-01	
2.61_218.1388m/z	20	0.646	[	1.80E-01	-	1.54E+00	]	35	0.674	[	2.75E-01	-	1.32E+00	]	1.050	318	5.85E-01	6.93E-01	
Paraxanthine	20	1.020	[	1.85E-02	-	5.41E+00	]	35	0.837	[	6.19E-02	-	5.32E+00	]	0.877	384	5.61E-01	6.93E-01	
5-Octenoylcarnitine (C8:1)	20	0.332	[	9.03E-02	-	9.50E-01	]	35	0.362	[	8.16E-02	-	1.34E+00	]	1.192	316	5.61E-01	6.93E-01	
0.98_72.0809m/z	20	10.602	[	8.87E+00	-	1.53E+01	]	35	12.662	[	6.23E+00	-	2.39E+01	]	1.084	323	6.46E-01	7.46E-01	
1.13_204.0211n	20	0.871	[	5.60E-01	-	1.11E+00	]	35	0.929	[	2.53E-01	-	1.67E+00	]	1.039	324	6.58E-01	7.46E-01	
LPC 20:4 [iso 2]	20	1.423	[	6.47E-01	-	6.36E+00	]	35	1.495	[	2.20E-01	-	9.16E+00	]	1.046	326	6.84E-01	7.58E-01	
Serotonin or cotinine	20	0.003	[	3.13E-03	-	1.87E+00	]	35	0.007	[	5.07E-05	-	1.11E+00	]	0.359	371	7.22E-01	7.84E-01	
LPC 18:2 [iso 1]	20	0.491	[	2.64E-01	-	2.11E+00	]	35	0.546	[	1.18E-01	-	4.73E+00	]	1.096	343	9.10E-01	9.28E-01	
2.23_86.0966m/z	20	12.174	[	9.90E+00	-	1.83E+01	]	35	13.315	[	6.46E+00	-	2.70E+01	]	1.025	343	9.10E-01	9.28E-01	
5.22_181.0720m/z	20	1.292	[	8.18E-03	-	5.55E+00	]	35	1.540	[	7.22E-02	-	6.33E+00	]	1.105	341	8.83E-01	9.28E-01	
5.40_180.0648n	20	0.344	[	1.50E-02	-	1.75E+00	]	35	0.345	[	4.20E-02	-	1.74E+00	]	1.022	350	1.00E+00	1.00E+00	

[I] The foldchange was calculated as $\bar{x}_{pku}/\bar{x}_{control}$.

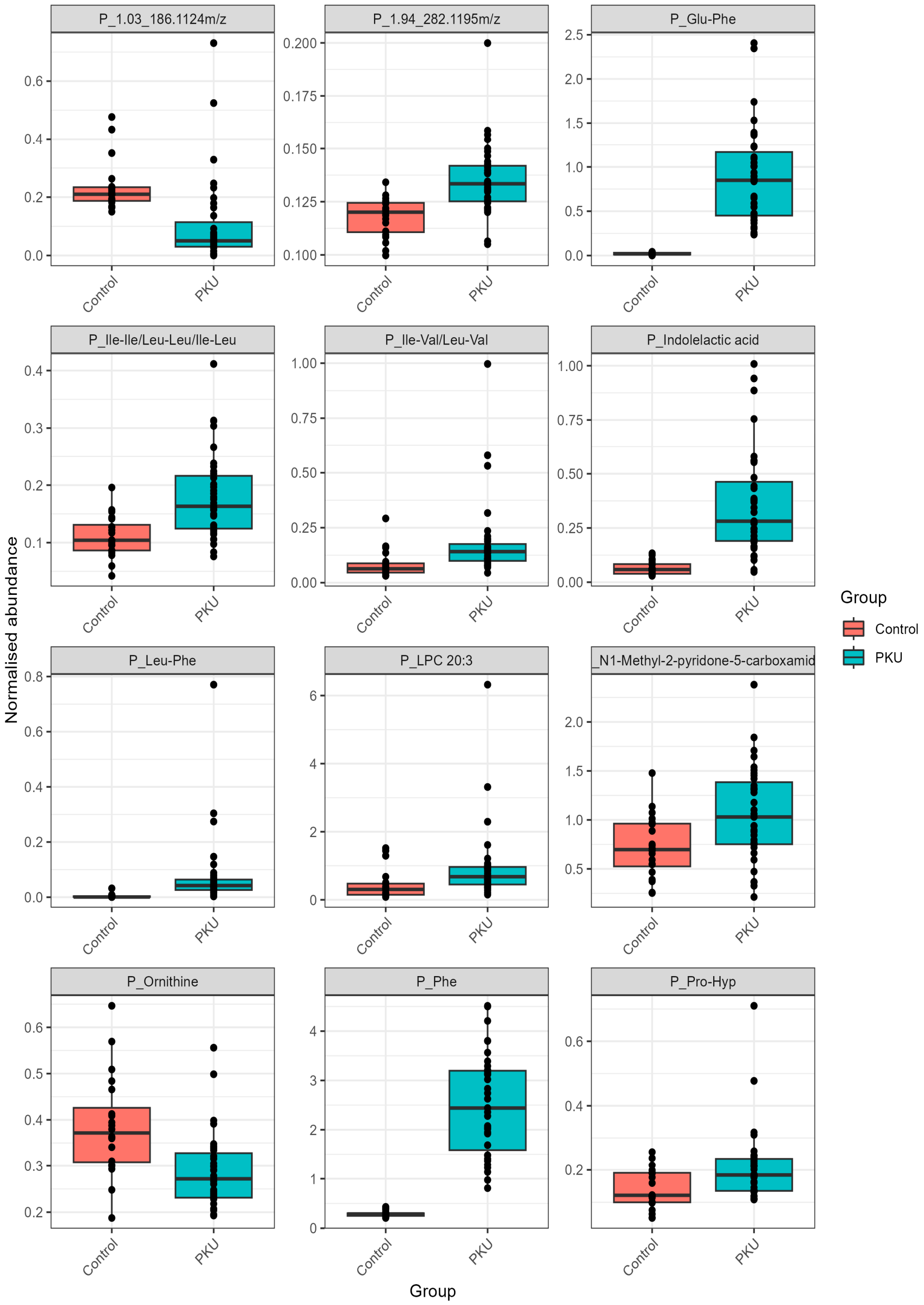

Figure S15. Statistically significant metabolite features in ESI(+), at p<0.05.

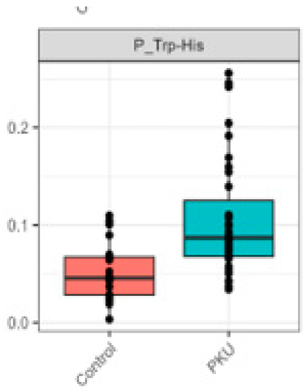

Figure S15 continued. Statistically significant metabolite features in ESI(+), at p<0.05.

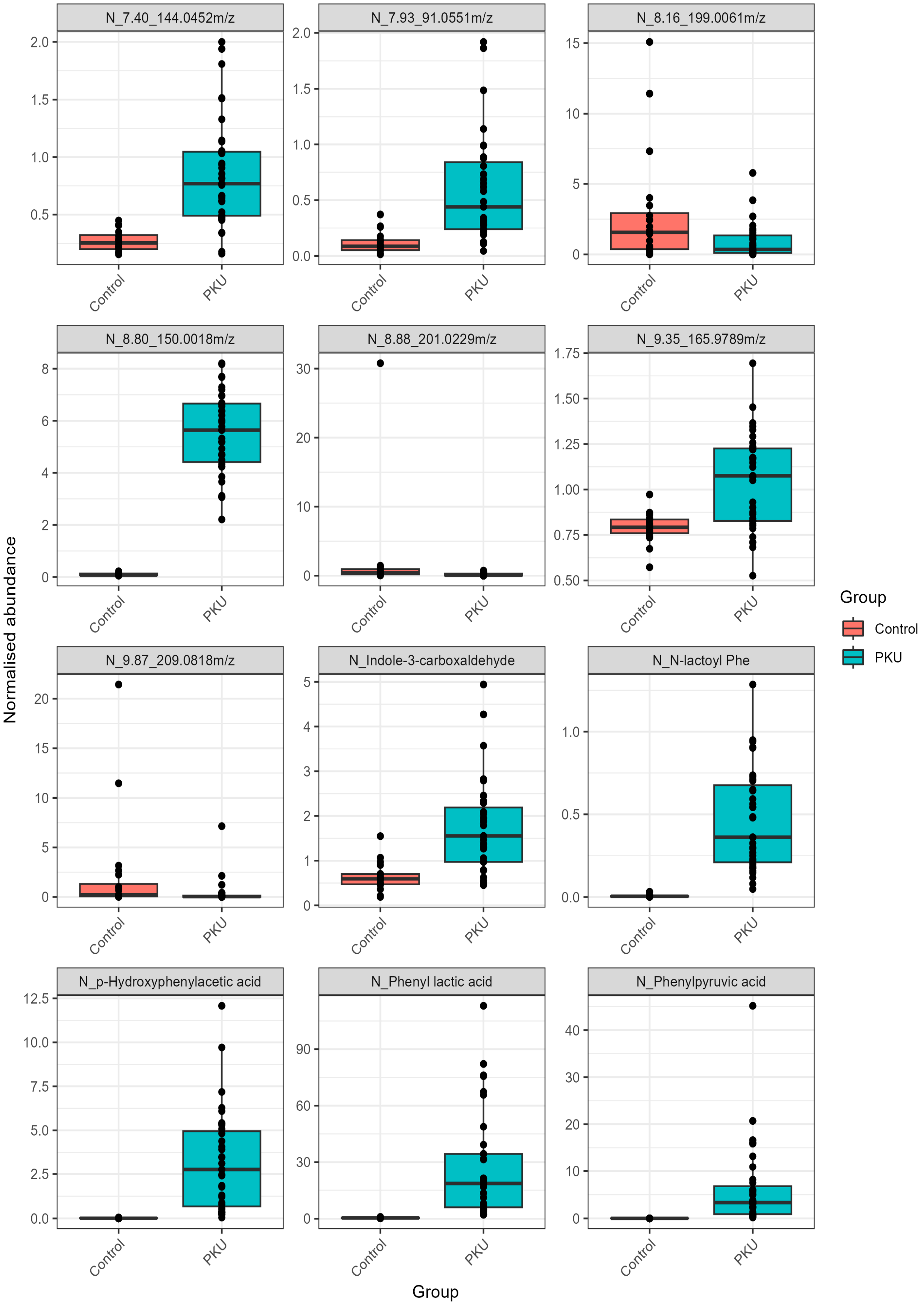

Figure S16. Statistically significant metabolite features in ESI(-), at p<0.05.

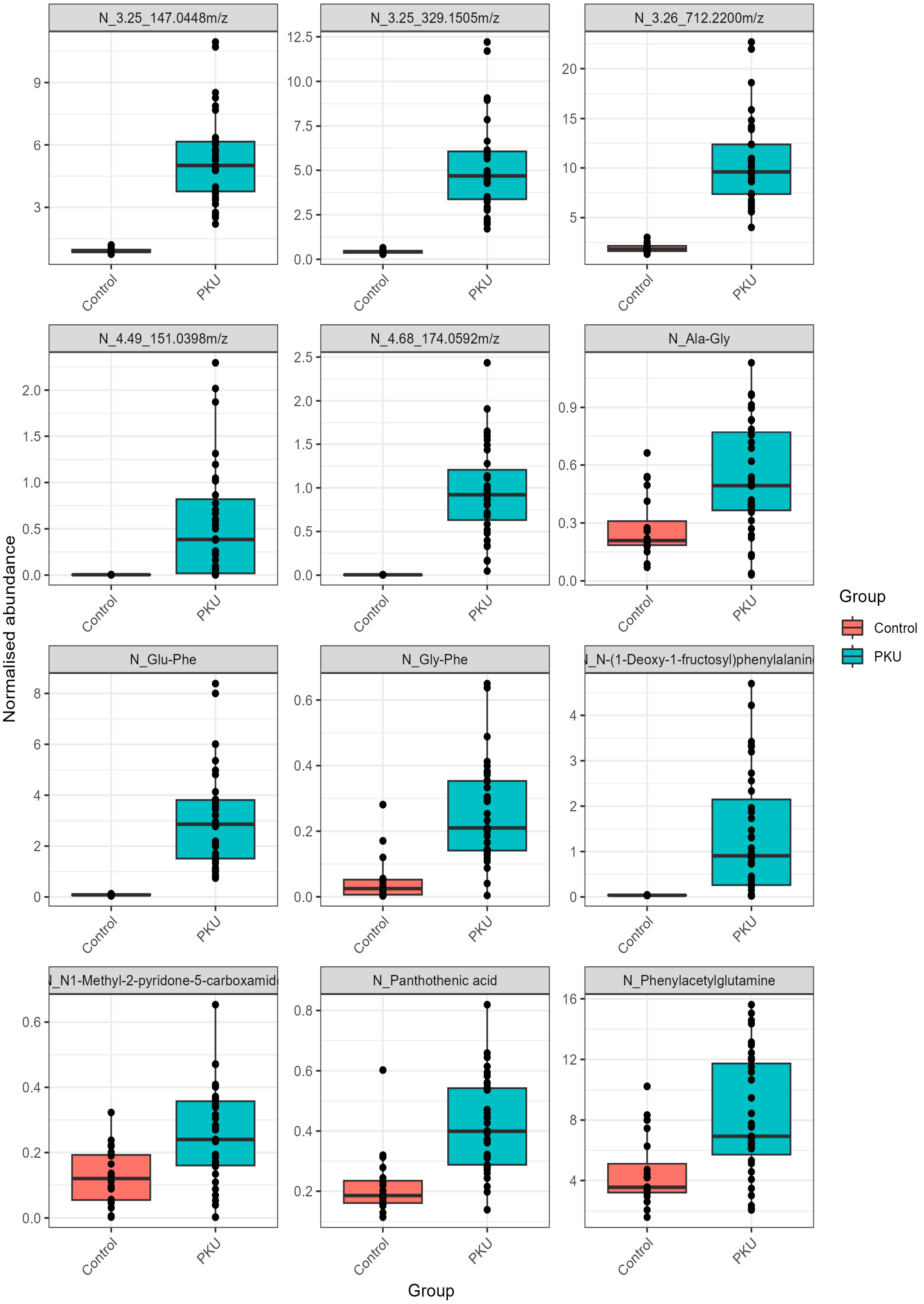

Figure S16. Statistically significant metabolite features in ESI(-) at $p<0.05$.

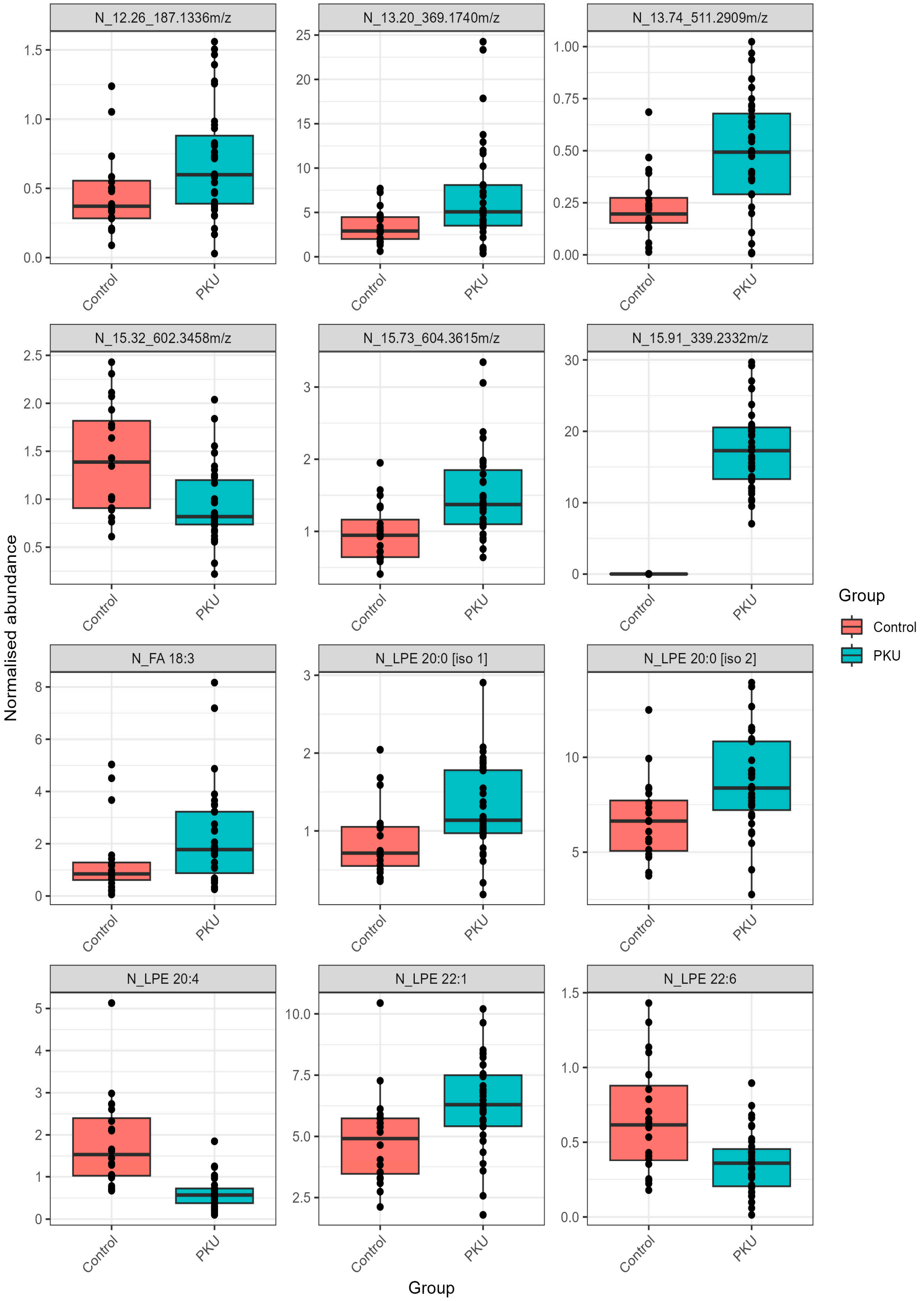

Figure S16. Statistically significant metabolite features in ESI(-), at $p<0.05$.

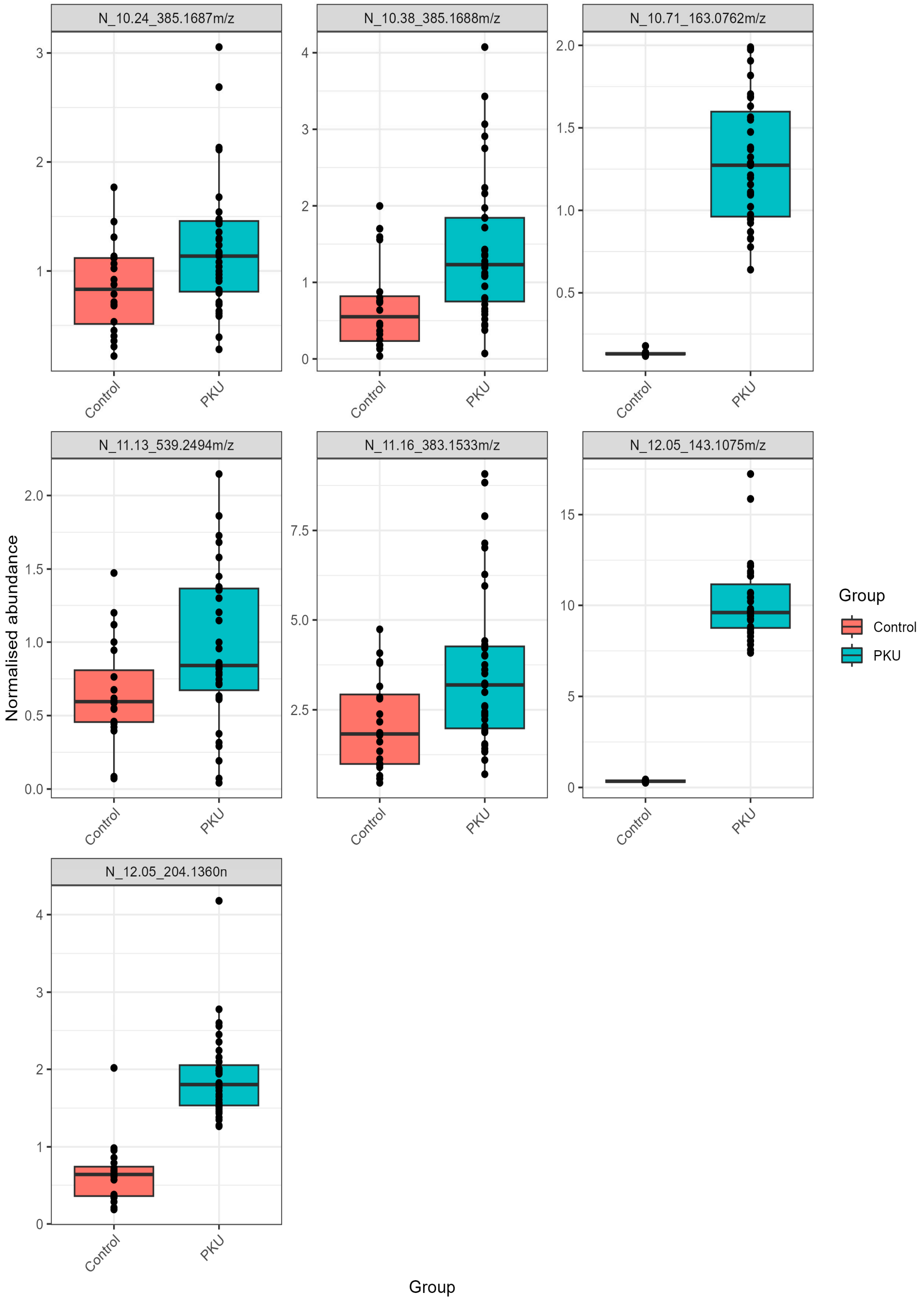

Figure S16. Statistically significant metabolite features in ESI(-) at $p<0.05$.

Table S14. Results Spearman correlations.

Type	Variabel	Correlation coefficient	p-value
Metabolomics	1.94_282.1195m/z	0.37	2.82E-02
Metabolomics	1.03_186.1124m/z	-0.20	2.59E-01
Metabolomics	Glu-Phe	0.95	7.91E-18
Metabolomics	Indolelactic.acid	0.81	5.37E-09
Metabolomics	Ile-Ile/Leu-Leu/Ile-Leu	0.27	1.13E-01
Metabolomics	Ile-Val/Leu-Val	0.12	5.09E-01
Metabolomics	Leu-Phe	0.06	7.46E-01
Metabolomics	LPC.20:3	0.23	1.76E-01
Metabolomics	Phe	0.88	2.45E-12
Metabolomics	N1-Methyl-2-pyridone-5-carboxamide	-0.04	8.34E-01
Metabolomics	Ornithine	-0.12	5.08E-01
Metabolomics	Pro-Hyp	-0.06	7.20E-01
Metabolomics	Trp-His	0.38	2.48E-02
Metabolomics	9.87_209.0818m/z	-0.09	6.14E-01
Metabolomics	9.35_165.9789m/z	0.28	1.06E-01
Metabolomics	8.88_201.0229m/z	-0.28	9.99E-02
Metabolomics	8.80_150.0018m/z	-0.04	8.22E-01
Metabolomics	8.16_199.0061m/z	0.06	7.50E-01
Metabolomics	7.93_91.0551m/z	0.83	8.73E-10
Metabolomics	N-lactoyl.Phe	0.73	8.15E-07
Metabolomics	Indole-3-carboxaldehyde	0.76	9.47E-08
Metabolomics	7.40_144.0452m/z	0.77	7.75E-08
Metabolomics	Phenyl.lactic.acid	0.88	2.40E-12
Metabolomics	Hydroxyphenylacetic.acid	0.87	7.93E-12
Metabolomics	Phenylpyruvic.acid	0.93	1.51E-15
Metabolomics	Phenylacetylglutamine	0.65	2.49E-05
Metabolomics	Ala-Gly	0.63	5.55E-05
Metabolomics	Glu-Phe	0.95	1.44E-18
Metabolomics	4.68_174.0592m/z	0.35	3.64E-02
Metabolomics	4.49_151.0398m/z	0.85	7.08E-11
Metabolomics	Panthothenic.acid	-0.22	2.11E-01
Metabolomics	Gly-Phe	0.33	5.53E-02
Metabolomics	N-(1-Deoxy-1-fructosyl)phenylalanine	0.32	6.09E-02
Metabolomics	3.26_712.2200m/z	0.50	2.20E-03
Metabolomics	3.25_329.1505m/z	0.85	7.51E-11
Metabolomics	3.25_147.0448m/z	0.89	8.66E-13
Metabolomics	N1-Methyl-2-pyridone-5-carboxamide	-0.10	5.52E-01
Metabolomics	FA.18:3	0.10	5.64E-01
Metabolomics	15.91_339.2332m/z	0.18	2.88E-01
Metabolomics	LPE.22:1	-0.01	9.77E-01
Metabolomics	15.73_604.3615m/z	0.21	2.35E-01
Metabolomics	LPE.20:0.[iso.2]	0.05	7.69E-01
Metabolomics	LPE.20:0.[iso.1]	0.02	9.22E-01
Metabolomics	LPE.22:6	0.10	5.62E-01
Metabolomics	LPE.20:4	0.07	6.99E-01

Metabolomics	15.32_602.3458m/z	0.09	6.26E-01
Metabolomics	13.74_511.2909m/z	0.00	9.96E-01
Metabolomics	13.20_369.1740m/z	0.34	4.84E-02
Metabolomics	12.26_187.1336m/z	0.09	6.05E-01
Metabolomics	12.05_204.1360n	0.07	6.94E-01
Metabolomics	12.05_143.1075m/z	0.34	4.63E-02
Metabolomics	11.16_383.1533m/z	-0.03	8.86E-01
Metabolomics	11.13_539.2494m/z	0.24	1.59E-01
Metabolomics	10.71_163.0762m/z	0.45	6.19E-03
Metabolomics	10.38_385.1688m/z	0.05	7.72E-01
Metabolomics	10.24_385.1687m/z	0.23	1.79E-01
Lipidomics	CE.18:2	0.08	7.41E-01
Lipidomics	CerPE.38:2;O2.[iso.2]	-0.06	7.99E-01
Lipidomics	PC.32:0	-0.20	3.80E-01
Lipidomics	PC.32:1	-0.41	6.59E-02
Lipidomics	PC.34:1	-0.09	6.83E-01
Lipidomics	PC.38:3	-0.23	3.13E-01
Lipidomics	PC.38:7.[iso.2]	-0.18	4.27E-01
Lipidomics	PC.40:6	-0.21	3.58E-01
Lipidomics	PC.42:6	-0.28	2.21E-01
Lipidomics	PC.O-34:2.or.PC.P-34:1	0.22	3.36E-01
Lipidomics	PC.O-44:4	-0.16	4.77E-01
Lipidomics	PI.40:3	-0.23	3.22E-01
Lipidomics	TG.45:5	-0.06	8.06E-01
Lipidomics	TG.50:1	0.28	2.27E-01
Lipidomics	TG.50:2	0.16	4.87E-01
Lipidomics	TG.56:7	0.04	8.80E-01
Lipidomics	TG.58:7	0.08	7.22E-01
Lipidomics	1.28_375.2526n	-0.18	4.33E-01
Lipidomics	3.23_384.1917n	0.07	7.67E-01
Lipidomics	11.67_360.1796n	-0.25	2.70E-01
Lipidomics	13.22_646.4526n	0.03	8.91E-01
Lipidomics	10.76_805.5592n	-0.30	1.84E-01
Lipidomics	11.62_833.6073n	-0.27	2.37E-01
Lipidomics	13.08_752.5923m/z	0.16	4.75E-01
Lipidomics	PI.36:4	-0.01	9.64E-01
Lipidomics	PI.38:3	-0.25	2.78E-01
Lipidomics	LPC.17:0	0.17	4.49E-01
Lipidomics	LPC.20:3	-0.22	3.43E-01
Lipidomics	PC.34:1	-0.08	7.20E-01
Lipidomics	PC.36:1	0.06	8.01E-01
Lipidomics	PC.36:5	-0.37	9.72E-02
Lipidomics	PC.38:3	-0.06	7.97E-01
